# Factors associated with youth gang membership in low‐ and middle‐income countries: a systematic review

**DOI:** 10.4073/csr.2018.11

**Published:** 2018-11-29

**Authors:** Angela Higginson, Kathryn Benier, Yulia Shenderovich, Laura Bedford, Lorraine Mazerolle, Joseph Murray

## Abstract

This Campbell systematic review examines the predictors of youth gang membership in low‐ and middle‐income countries. The review summarises findings from eight reports from five countries and the Caribbean region.

The lack of available evidence limits the extent to which clear conclusions can be drawn about the factors associated with youth gang membership. The review is based on a very small number of studies, and has significant limitations in coverage. The limited evidence of the correlates of youth gang membership suggests factors that may drive gang membership and suggests areas where interventions may prove promising in the family, school, and community domains, as well as provide a starting point for future studies.

**Plain language summary:**

**Evidence shows which factors predict gang membership in low‐ and middle‐income countries, but more studies needed**

Youth gang membership is associated with delinquency, violent crime and trafficking. A range of individual, peer, family, school and community factors can predict the likelihood of youths getting involved with gangs. Knowledge of these factors can be helpful for reducing gang membership.

**What is this review about?**

Youth gang membership is associated with delinquency, violent crime and trafficking – and gang members are themselves frequently the victims of these offences. Yet youth gangs can also provide a form of social capital, a sense of belonging and purpose to disenfranchised youth.

This review identifies the factors associated with young people joining gangs, and the differences between gang‐involved and non‐gang‐involved youth. Understanding these associations is essential to reduce the levels of gang membership and the incidence of related violence.

**What is the aim of this review?**

This Campbell systematic review examines the predictors of youth gang membership in low‐ and middle‐income countries. The review summarises findings from eight reports from five countries and the Caribbean region.

**What studies are included?**

Studies of youth gangs in in low‐ and middle‐income countries were included, with participants aged 10‐29 years. The studies had to assess an individual predictor or correlate of youth gang membership, where the predictor or correlate is a single characteristic, not a conglomeration of multiple constructs. Included studies had designs including data on both gang‐involved and non‐gang‐involved youth, recruited with strategies that were eligible.

Nine studies met the eligibility criteria and were included in the review. One of these studies did not report all the required data and so was not included in the analyses. The studies were conducted in Turkey, Trinidad and Tobago, the Caribbean, El Salvador, China and Brazil.

**Factors associated with gang membership**

**Table:** 

**Domain**	**Significantly associated with gang membership**	**No significant association with gang membership**
Individual	Delinquency Alcohol and soft drug use Male gender Risky sexual behaviours Employment Psychological risk factors (low self‐control, impulsivity) and lack of psychological protective factors (empathy, future orientation, belief in moral order) Victimisation	Age Minority ethnicity Protective behaviours surrounding sexual behaviour or alcohol and soft drug use.
Peer	Socialising with delinquent peers	Socialising with pro‐social peers
Family	Negative family environments Lack of parental monitoring Middle‐income families had greater odds of reporting youth gang membership than those from either high or low‐income families (finding from one study)	Parental education Parental attitudes to antisocial behaviour
School	Low school attachment Exposure to violence at school Educational difficulties Opportunities for prosocial involvement	Level of education School type School performance
Community	Exposed to neighbourhood violence	Neighbourhood environment risk or protective factors, or geography

**What are the main results in this review?**

The eight studies analysed in the review address the associations between life events and circumstances, and the likelihood of being a youth gang member across five domains: individual, peers, family, school and community. Significant associations were found with factors in each domain.

**What do the findings of this review mean?**

The lack of available evidence limits the extent to which clear conclusions can be drawn about the factors associated with youth gang membership. The review is based on a very small number of studies, and has significant limitations in coverage. The limited evidence of the correlates of youth gang membership suggests factors that may drive gang membership and suggests areas where interventions may prove promising in the family, school, and community domains, as well as provide a starting point for future studies.

**How up‐to‐date is this review?**

The review authors searched for studies published up to September 2013. This Campbell systematic review was published in December 2018.

**Summary:**

**Background**

Youth gang membership is well documented throughout low‐ and middle‐income countries, and youth gang members are increasingly associated with delinquency, violent crime and trafficking. They are also frequently the victims of these offences, often in disproportionate numbers compared to non‐gang youth. Yet youth gangs can also provide a form of social capital, a sense of belonging and purpose to disenfranchised youth.

Extensive research, primarily from high‐income countries, has categorized five domains of risk and protective factors for youth gang involvement, drawn from the realm of developmental psychology. These domains are: Individual, Peer, Family, School, and Community. Youth gang membership is seen as the culmination of interrelated structural and process factors, which in combination with negative life events may increase the attractiveness of gang membership.

This review aimed to identify the factors associated with young people joining gangs, and to identify and quantify the differences between gang‐involved and non‐gang‐involved youth. Understanding these associations is essential to reduce the levels of gang membership and the incidence of related violence.

**Objectives**

This review addresses two key objectives: (1) to synthesize the published and unpublished empirical evidence on the factors associated with membership of youth gangs in low‐ and middle‐income countries;(2) to assess the relative strength of the different factors across the domains of individual, family, school, peer group and community.

**Search methods**

The search was conducted in English, French, Chinese, Arabic, Russian, Spanish and Portuguese. We searched 55 locations including academic databases, journals, and grey literature locations, and located over 54,000 documents to screen.

**Selection criteria**

We included studies meeting the following criteria:

- Reports on youth gangs
- All participants aged 10‐29 years
- Located in low‐ or middle‐income country
- Assesses an individual predictor or correlate of youth gang membership
- Predictor or correlate is a single characteristic
- Predictor or correlate is not a conglomeration of multiple constructs
- Eligible recruitment strategy for respondents
- Study design included data on both gang involved and non‐gang involved youth.

**Data collection and analysis**

We conducted a broad abstract screening of over 54,000 titles and abstracts, followed by a close abstract screening of 1509 abstracts. We screened the full‐text of 98 documents. Nine studies met the eligibility criteria and were included in the review. One of these studies did not report sufficient data to allow the calculation of a standardized effect size, and so was not included in the analyses. A total of 85 independent effect sizes were extracted from the eight studies with sufficient data to create a standardized effect size. We calculated Cohen's *d* from continuous data and the Log Odds Ratio from dichotomous data. All effects were categorized into the five predictor domains, and further classified into conceptually similar group and risk or protective factors. We synthesized the data using multiple random effects meta‐analyses with inverse variance weighting.

**Results**

The eight studies analysed in the review address the associations between life events and circumstances, and the likelihood of being a youth gang member. All studies were based on cross‐sectional survey data, using different statistical methods to identify correlations between youth characteristics and the likelihood of being a member of a youth gang. No longitudinal, prospective or retrospective studies were located. The studies were conducted in Turkey, Trinidad and Tobago, the Caribbean, El Salvador, China and Brazil. We organized the analyses according to five domains of factors that may be associated with youth gang involvement and summarize the main findings below:

*Individual factors*

- Delinquency, alcohol and soft drug use, male gender, risky sexual behaviours, employment, psychological risk factors (low self‐control, impulsivity), and victimisation were each associated with significantly higher odds of youth gang membership.
- Psychological protective factors (empathy, future orientation, belief in moral order) were associated with lower odds.
- No association between youth gang membership and age, minority ethnicity, or protective behaviours surrounding sexual behaviour or alcohol and soft drug use.

*Peer factors*

- There is a small amount of evidence that socialising with delinquent peers is associated with increased odds of youth gang membership, but there is no significant relationship demonstrated between socialising with pro‐social peers and youth gang membership.

*Family factors*

- Negative family environments are associated with more youth gang membership, while both a positive family environment and parental monitoring were associated with lower odds.
- One study showed that youth from middle‐income families had greater odds of reporting youth gang membership than those from either high or low‐income families.
- No significant relationship was seen between youth gang membership and parental education or parental attitudes to antisocial behaviour.

*School factors*

- Low school attachment, exposure to violence at school, educational difficulties and opportunities for prosocial involvement all show significant association with more youth gang membership.
- No significant relationship was seen between youth gang membership and level of education, school type, or school performance.

*Community factors*

- There were significantly higher odds of youth gang membership amongst those who reported that they were exposed to violence in their neighbourhood.
- No demonstrated association between youth gang membership and neighbourhood environment risk or protective factors, or geography.

Gang membership is typically viewed as a culmination of interrelated structural and process factors. Understanding the factors associated with youth gang membership can help inform prevention strategies to reduce the levels of youth gang membership and the incidence of youth gang violence. Unfortunately the small number of studies contributing to any analysis limits the conclusions that can be drawn from this study. Moreover, many of the individual and peer associations identified in this review (such as delinquency, drug use, and sexual risk factors) may be as a result of gang membership rather than its cause. However, our results suggest certain family, school, and community level factors associated with gang membership that could be addressed through targeted preventive interventions, particularly family environment, parental monitoring, school attachment, educational difficulties, and exposure to violence in the home, at school, or in the community.

**Authors’ conclusions**

The lack of available evidence limits the extent to which we can draw any clear conclusions about the factors associated with youth gang membership. This current review is based on a very small number of studies, and has significant limitations in coverage; however it provides some limited evidence of the correlates of youth gang membership. Specifically, this review suggests factors that may drive gang membership and suggests areas where interventions may prove promising in the family, school, and community domains, as well as provide a starting point for future studies.

## Background

### The issue

Gang violence remains an issue in low‐ and middle‐income countries in Africa and Asia, and the prevalence of gangs is particularly well documented throughout Central and South America (Decker and Pyrooz, 2010; Gatti *et al*., 2011). Official estimates of gang membership in Central America estimate approximately 69,000 members, while academic estimates believe this figure to be closer to 200,000 (UNODC, 2007). Some estimates are as high as 500,000 gang members in the region including South America and the Caribbean, and gangs have been identified as “the primary threat to regional stability and security” (Muggah and Aguirre, 2013). While reporting and recording issues make it difficult to estimate rates of gang violence, the homicide rate in Colombia, Brazil, El Salvador and Guatemala are substantially higher than those of European and North American countries (Decker and Pyrooz, 20 UNODC, 2007). Gangs are also active in South Africa, with an estimate of 100,000 members in Western Cape alone (Reckson and Becker, cited in Decker and Pyrooz, 2010); however, to date, there is limited research examining gangs in Africa and Asia.

Youth gangs are internationally associated with increased rates of delinquency and violent crime (Howell, 1997; Klein, 2002; White, 2002), including trafficking in arms, drugs and (increasingly) humans (Organisation of American States [OAS], 2007). The victims of gang crime are not only non‐gang‐affiliated individuals and rival gang members, but also include members of the same gang. Gang members are disproportionately involved with serious and violent offences compared to non‐gang delinquent youth (Howell, 1998). This suggests that something about gang membership encourages violence over and above the correlation between having delinquent friends and a previous delinquent history (Battin *et al*., 1998; Haviland *et al*., 2008).

Researchers often contest a uniform definition of a youth gang, as it varies by time and place (Howell, Egley, and O'Donnell, n.d.). Notwithstanding these debates, the literature typically describes a gang as comprising between 15 to 100 members, generally aged 12 to 24; members share an identity linked to name, symbols, colours or physical or economic territory; members and outsiders view the group as a gang; there is some permanence and degree of organisation; and there is involvement in an elevated level of criminal activity (Decker and Curry, 2003; see also Esbensen *et al*., 2001; Howell *et al*., n.d.; Huff, 1993; Miller, 1992; Rodgers, 1999; Spergel, 1995; Theriot and Parker, 2008). There have been significant efforts amongst academics and policy makers to reach agreement on the definition of a youth gang. The “Eurogang Working Group” (see The Eurogang Project, 2012) consensus definition is as follows: “A street gang (or troublesome youth group corresponding to a street gang elsewhere) is any durable, street‐oriented youth group whose involvement in illegal activity is part of its group identity” (Weerman et. al., 2009, p.20). A youth gang is differentiated from an adult gang if the majority of the gang members are aged between 12 and 25 (Weerman et. al., 2009).

Although associated with criminal activity, gangs can offer a sense of belonging and purpose to disenfranchised youth (Howell, 2012; Tobin, 2008). Self‐reported reasons for gang membership can include social reasons, protection, and instrumental or financial reasons (Howell and Egley, 2005). For young men living in environments of deprivation, exclusion and violence, having family members in gangs may lead to them learning to ‘do masculinity’ in a context of “exposure and socialisation into armed groups”, particularly where pro‐social opportunities are limited (Baird, 2012, p.186). Humiliating levels of deprivation may lead to the search for an extreme public masculinity that provides the gang member with power or ‘respect’ (Adams, 2012). Gang membership can be viewed as a means to overcome “extreme poverty, exclusion, and a lack of opportunities” (Organization of American States (OAS), 2007, p.5).

*“Youth gangs represent a spontaneous effort by children and young people to create, where it does not exist, an urban space in society that is adapted to their needs, where they can exercise the rights that their families, government, and communities do not offer them. Arising out of extreme poverty, exclusion, and a lack of opportunities, gangs try to gain their rights and meet their needs by organizing themselves without supervision and developing their own rules, and by securing for themselves a territory and a set of symbols that gives meaning to their membership in the group. This endeavour to exercise their citizenship is, in many cases, a violation of their own and others’ rights, and frequently generates violence and crime in a vicious circle that perpetuates their original exclusion. This is why they cannot reverse the situation that they were born into. Since it is primarily a male phenomenon, female gang members suffer more intensively from gender discrimination and the inequalities inherent in the dominant culture.”* (OAS, 2007, p.5)

In low‐ and middle‐income countries in particular, gang membership has been identified as offering a unique social framework for excluded youth to meet particular social and cultural needs (OAS, 2007); a process that has been described as “filling a social vacuum” (Adams, 2012, p.31).

### Factors associated with youth gang membership

Extensive research (primarily conducted in high‐income countries) has focused on identifying risk and protective factors which may alter the likelihood of youth becoming involved in violent activity. These have been categorized into individual, peer group, family, school, and community factors (Decker *et al*., 2013; Hawkins *et al*., 2000; Howell, 2012; Howell and Egley, 2005; Katz and Fox, 2010; Klein and Maxson, 2006; O'Brien *et al*., 2013; Tobin, 2008). These five domains are drawn from developmental psychology, where they are identified as the key domains of influence affecting a young person's behaviour (Howell and Egley, 2005).

We recognize that in some instances, these factors may be either a predictor of gang membership or a consequence of having joined a gang. In this review we use the broader term “factors” rather than “predictors” as the causal associations are at many times unclear or unsupported, and distinguish between predictors and correlates of gang membership according to the methodology used in the primary research (for more detail see the ‘Study design’ subsection of the ‘Criteria for inclusion and exclusion of studies’).

Individual factors include biological and psychological characteristics identifiable in children from young ages that may increase vulnerability to negative social and environmental influences (Herrenkohl *et al*., 2000). Peer group factors that may influence youth gang involvement include peer attitudes, delinquency and gang involvement (Dahlberg, 1998; Katz and Fox, 2010; Moser and Holland, 1997; Olate *et al*., 2012). Family factors refer to both the structural characteristics of families, such as poverty, single‐headed households, as well as the way in which children are socialized within families (Blum *et al*., 2003; Howell and Egley, 2005; Moser and Holland, 1997; Thale and Falkenburger, 2006). School factors include such aspects as children's academic achievement and experiences at school, including exposure to violence (Herrenkohl *et al*., 2000; Howell and Egley, 2005; Olate *et al*., 2012). Community factors are the structural and social characteristics of the local environment, including neighbourhood levels of crime, firearms and drugs in a neighbourhood (Katz and Fox, 2010; Moser and Holland, 1997; Sanders *et al*., 2009; Thale and Falkenburger, 2006; Tobin, 2008) as well as factors such as community social disorganisation (Howell, 2012; Howell and Egley, 2005). A summary of factors associated with gang membership is shown in Table 1.

**Table 1:** Summary of factors associated with youth gang membership

Domain	Risk factors	Protective factors
Individual	Prior delinquency Deviant attitudes Street smartness; toughness Defiant and individualist character Fatalistic view of the world Aggression Proclivity for excitement and trouble *Locura* (acting in a daring, courageous, and especially crazy fashion in the face of adversity) Higher level of normlessness in the context of family, peer group, and school Social disabilities Illegal gun ownership Early or precocious sexual activity, especially among females Alcohol and drug use Drug trafficking Desire for group rewards such as status, identity, self‐esteem, companionship, and protection Problem behaviours, hyperactivity, externalizing behaviours, drinking, and lack of refusal skills Victimization	High level of personal resources Sense of coherence Positive, culturally relevant identity
Peer group	High commitment to delinquent peers Low commitment to positive peers Street socialization Gang members in class Friends who use drugs or who are gang members Friends who are drug distributors Interaction with delinquent peers	Mixed peer network of gang and non‐gang members Intimate partner attachment to non‐gang affiliate
Family	Family disorganization, including broken homes and parental drug or alcohol abuse Troubled families, including incest, family violence, and drug addiction Family members in a gang Lack of adult male role models Lack of parental role models Low socio‐economic status Extreme economic deprivation, family management problems, parents with violent attitudes, sibling anti‐social behaviour	Family involvement Consistent parental discipline Open family communication
School	Academic failure Low educational aspirations, especially among females Negative labelling by teachers Trouble at school Few teacher role models Educational frustration Low commitment to school, low school attachment, high levels of anti‐social behaviour in school, low achievement test scores, identification as being learning‐disabled	Psychosocial support for teachers Parental involvement in schools
Community	Social disorganization, including poverty and residential mobility Organized lower‐class communities Underclass communities Presence of gangs in the neighbourhood Availability of drugs in the neighbourhood Availability of firearms Barriers to and lack of social and economic opportunities Lack of social capital Cultural norms supporting gang behaviour Feeling unsafe in neighbourhood; high crime Conflict with social control institutions	Short or no history of gang presence Strict formal and informal control of firearms Limited neighbourhood congregation sites of unsupervised youth Absence of drug markets

Source: adapted from Small Arms Survey, 2010, pp.236‐237.

Previous research conducted within high‐income countries provides evidence of the importance of individual, peer and family domains as factors associated with youth gang involvement, whilst relatively weaker evidence exists for the value of school and community factors (O'Brien *et al*., 2013). The present review seeks to examine whether the relative weight of influence across these domains also applies to youth gang involvement in low‐ and middle‐income countries.

### How the factors may affect gang membership

Research indicates that each of the five domains associated with youth gang involvement (individual, peer, family, school and community) are most influential at particular times in a child or young person's life, and that a developmental model is useful to identify the key steps towards offending behaviour (Howell and Egley, 2005). Research in high‐income countries demonstrates that the factors associated with gang involvement cut across all five domains, that youth with multiple risk factors have a proportionately higher risk of gang involvement, and that those youth with risk factors in multiple domains have further increased likelihood of gang involvement (Decker *et al*., 2013; Howell and Egley, 2005).

**Figure 1:**
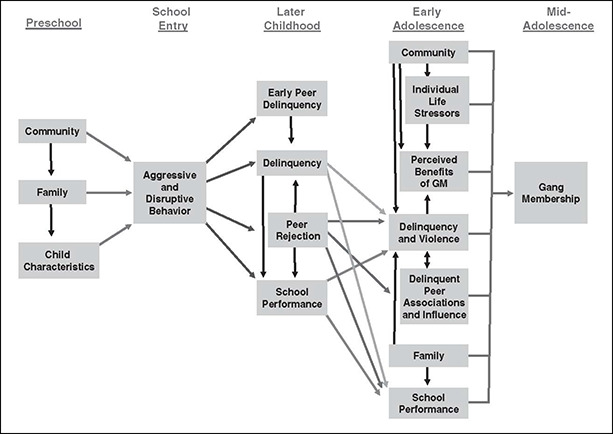
Logic model of predictors of gang membership (Source: Howell and Egley, 2005)

Building on Thornberry and colleagues’ developmental framework of gang membership (Thornberry *et al*., 2003), Howell and Egley (2005) propose a developmental perspective that incorporates these factors from early childhood through to adolescence. The model is illustrated in Figure 1.

Howell and Egley (2005) argue that the pathway to gang membership for youth at the highest risk begins as early as three or four years of age with conduct problems, school failure in elementary school, followed by delinquency at 12 years of age, gang membership in early adolescence and more serious delinquency from mid‐adolescence. We describe Howell and Egley's (2005) developmental model in the remainder of this section.

Howell and Egley's logic model of gang membership (2005) begins with preschool factors, where structural disadvantage and lack of social capital at the community level, combined with family factors such as low human capital, family conflict and poor parenting, and child level risk factors such as aggressive and impulsive temperament, are theorized to lead to conduct disorders at the pre‐school stage. These aggressive and disruptive behaviours may lead to rejection by pro‐social peers, which may increase the likelihood of early delinquent behaviour and decreased school performance. In later childhood, it is suggested that peer factors become even more important. Early rejection by pro‐social peers may increase the likelihood of association with aggressive or delinquent peers, and therefore the likelihood of further delinquent behaviour and the weakening of social bonds. School level factors such as poor grades, low‐quality schooling or school policies such as suspension or expulsion, may also increase the likelihood of gang membership due to the weakening of school‐student bonds and the potential for increased time without adult supervision.

In early adolescence it is argued that the influence of community level predictors increases. Community factors such as high crime rates, drug use, and concentrated disadvantage may lead to decreased informal social control and decreased community attachment. This may lead to negative life stressors, delinquency, and the perception that gang membership offers benefits to the young person. Negative family characteristics (both structural and social process factors) are theorized to continue to affect young people by decreasing family bonds, increasing delinquency and reducing school performance. School risk factors such as poor academic performance, low aspirations, negative labelling by teachers and feeling unsafe at school may reduce attachment and increase the risk of gang membership. The model suggests that delinquent beliefs and delinquent peers in early adolescence, and individual predictors including substance use, delinquency and life stressors such as violent victimisation further increase the likelihood of delinquency and violence, a key precursor of youth gang membership.

Gang membership is seen as a culmination of interrelated structural and process factors. It is argued that individual, community and structural family characteristics influence early pro‐social behaviours and pro‐social bonds. In an interactive feedback relationship, antisocial behaviours may decrease pro‐social friendships and in turn increase the impact of negative peer attachments and the risk of delinquent behaviours. These social and structural factors, in combination with negative life events, negative school experiences and a lack of school attachment, may increase the attractiveness of gang membership.

### Why it is important to do the review

Understanding the factors associated with youth gang membership is essential to designing empirically‐based prevention strategies to reduce the levels of youth gang membership and the incidence of youth gang violence. The proposed systematic review aims to synthesize the research evidence that identifies the pathways to youth gang membership in low‐ and middle‐income countries.

The Campbell Collaboration has previously published three systematic reviews that examine the involvement of young people in gangs (Fisher *et al*., 2008a, 2008b; Higginson *et al*., 2015). The focus of the two reviews by Fisher *et al*. (2008a, 2008b) was on preventing youth gang involvement through cognitive‐behavioural and opportunities provision interventions, and these two systematic reviews found no studies that met their inclusion criteria. Another review of interventions designed to reduce gang‐related crime was conducted by the Evidence for Policy and Practice Information and Co‐ordinating Centre (EPPI‐Centre, 2009). Finally, a review by Higginson *et al*. (2015) focused on preventive gang interventions implemented in low‐ and middle‐income countries. The authors did not find any studies meeting the inclusion criteria, confirming the lack of empirical evidence on the effects of interventions to prevent youth gangs and violence.

These reviews have not considered the factors associated with youth gang membership, and three of the four reviews have focused on interventions implemented in high‐income countries. Klein and Maxson (2006) conducted a systematic review of the published evidence on risk factors for youth gang membership; however this review again focused on surveys conducted in the United States, Canada and Europe.

We suggest that there are differences in the motivations for participation in gangs between youth in high‐income countries and those in low‐ and middle‐income countries. This is evidenced in Olate *et al*.,'s(2011) cross‐cultural study, which identifies significant differences in the factors associated with youth gang membership between San Salvador and Boston, particularly with regards to early delinquency and violence. Many low‐ and middle‐income countries have experienced in recent decades or are experiencing some form of war or conflict, creating societies that foster youth gang membership. Issues such as a culture of violence, low sense of citizen security, distrust of authorities, poor economic outlook, high accessibility to firearms and drugs, and migration enable the creation and maintenance of gangs in such countries (Cruz, 2007; Davies and MacPherson, 2011; Thale and Falkenburger, 2006). We therefore focus our review on the factors associated with youth gang membership in low‐ and middle‐income countries, as defined by the World Bank (World Bank, 2013).

This review aims to inform not only the academic literature on the factors associated with youth gang membership, but aim to provide a valuable resource for both policy makers and practitioners to assist in designing appropriate preventive interventions for implementation.

Preventive gang interventions in low‐ and middle‐income countries are funded and implemented by NGOs, government agencies, international aid agencies, and community organisations. This systematic review has been funded by the United States Agency for International Development (USAID), with the aim of informing best practice in youth gang interventions. USAID supports a variety of preventive anti‐gang programs in Latin America and the Caribbean, including both primary and secondary prevention programs, and argues that evaluation is important to improve programs and build support for crime prevention programs (USAID, 2010b).

By identifying the most important factors associated with youth gang involvement and disseminating that information to those working in the field, we aim to ensure that policy makers and implementing agencies have access to high quality research when designing their interventions. The Campbell Collaboration systematic review of the impact of preventive interventions on youth gang involvement in low‐ and middle‐income countries highlights the dearth of robust quantitative evaluations of such interventions (Higginson *et al*., 2015). Despite a comprehensive search strategy, this systematic review found no impact evaluations of preventive gang interventions. Given the lack of evidence on the impact of interventions to prevent youth gang involvement in low‐ and middle‐income countries, it is important to synthesize the available evidence on factors associated with youth gang membership to inform the development of preventive interventions.

## Objectives

This review focuses on the factors associated with membership in youth gangs in low‐ and middle‐income countries and identifies multiple factors of interest.

This review has two key objectives: (1) to synthesize the published and unpublished empirical evidence on the factors associated with membership of youth gangs in low‐ and middle‐income countries;(2) to assess the relative strength of the different factors across the domains of individual, family, school, peer group and community.

## Methods

Unless otherwise stated, this review follows the methods outlined in its published protocol (Higginson et al., 2014).

### Criteria for considering studies for this review

This systematic review aims to determine the association between a characteristic of a young person or their environment and their gang membership status. This review focuses on observational studies rather than experimental or quasi‐experimental studies, as youth gang membership is not a characteristic that can be experimentally manipulated. Consequently, this review is interested in the factors associated with youth gang membership, and these factors may be categorized as either predictors or correlates. In order to describe the relationship as a predictive relationship, the “predictor” must occur prior to the onset of gang membership or be a time‐invariant characteristic. Ideally studies that examine predictors would be longitudinal; however there are few longitudinal studies examining gang membership and most studies in this field are cross‐sectional (Thornberry, 1999). We utilize cross‐sectional studies, but classify time‐variant factors as “correlates” in this instance, as it can be difficult to determine if a time‐variant characteristic is a true antecedent of the outcome if the study is not longitudinal (Murray *et al*. 2009).

#### Types of participants

There is a general agreement amongst researchers that most members of youth gangs are aged between 12 and 24 years of age (Howell *et al*., n.d.; Huff, 1993; Rodgers, 1999; Seelke, 2013). However, we extended the age range to include studies where the participants are aged between 10 and 29, in part because formal definitions of youth vary across countries, and in part to ensure that the age range is broad enough to ensure that studies that retrospectively examine youth gang membership within a short timeframe are not excluded.

We adopted a broad definition of youth gang membership. We acknowledge that there is no clear international consensus definition of youth gangs. As such, we accepted youth gangs as defined by the Eurogang definition: “a street gang (or troublesome youth group corresponding to a street gang elsewhere) is any durable, street‐oriented youth group whose involvement in illegal activity is part of its group identity” (Weerman *et al*., 2009, p.20). Likewise we accepted author definitions of youth gangs. We excluded groups described as organised crime gangs, terrorist gangs and piracy gangs.

This review is focused on the factors associated with youth gang membership in low‐ and middle‐income countries; therefore, we only included studies that take place in countries that have been classified by the World Bank as low‐ and middle‐income countries for at least 50 per cent of the time since 1987, when recordings start (World Bank, 2013).

#### Types of factors

For a factor to be considered a true predictor, it needs to be present prior to the outcome occurring, making longitudinal designs the optimal study method for identifying predictive factors (Farrington and Loeber, 2000). However, many studies of gang‐involved youth use a cross‐sectional study design, in which some factors are retrospectively reported or are clearly in existence prior to gang involvement (for example, sex, ethnicity), whilst some factors are only measured once the young person is already in a gang (for example, family conflict, expulsion from school). We recognize that measuring the factor at the same time as measuring the outcome has the potential to conflate the causes of gang membership with the results of gang membership (Klein and Maxson, 2006).

We classify *predictors* as those factors that are either:

1. estimated from prospective longitudinal studies at a time prior to the onset of gang membership, or
2. estimated from cross‐sectional studies and the factor is time‐invariant (eg. sex), or
3. estimated from longitudinal or cross‐sectional studies and the factor has been reported retrospectively to a time prior to onset of gang membership (e.g. number of family members who were gang members when the respondent was aged 10, parent's marital status when the respondent was aged 5).
4. estimated from a case‐control study where predictive factors are assessed retrospectively for samples of gang members (cases) and non‐gang members (controls).

We classify *correlates* as those factors that are either:

1. estimated from longitudinal studies at a time after the onset of gang membership, or
2. estimated from cross‐sectional studies without retrospective reporting to a time prior to the onset of gang membership.

We follow Klein and Maxson (2006) in including these cross‐sectional studies in order to retain more sources of evidence in our review; however, we synthesized the effect sizes for predictors and correlates separately.

We excluded factors that are conglomerations of multiple constructs across different domains, such as Raine *et al*.'s (1996) measure of biosocial risk, which combines measures of marital conflict, maternal rejection, family instability, parental crime, neurological problems, and slow motor development.

#### Types of outcome measures

The outcome of interest is membership in youth gangs. We coded outcomes related to individual youth participation in gangs, including self‐reported, peer‐reported, family‐reported, practitioner‐reported, or police‐reported measures of youth gang membership. We planned to perform moderator analysis to identify heterogeneity due to different methods of recording gang membership; however all eligible studies used self‐reported gang membership, so these analyses were not performed.

#### Study design

For inclusion in the review, studies must have used a sample where there was variability in the levels of gang membership, including youth who are not gang‐affiliated. For example, the sample may have included young people who were gang members, young people who were not gang members, and young people who were ex‐ gang members. We included observational longitudinal studies, cross‐sectional studies, case‐control studies, and epidemiological studies, as long as they included a subsample of young people who were not gang members. Studies must have provided a bivariate or multivariate assessment of the relationship between a factor and gang membership.

We did not include studies that reported only on the characteristics of a youth gang sample with no reference to a comparison group. In such studies there is no way to demonstrate that gang‐involved and non‐gang‐involved youth differ on these measures. While single case studies and ethnographies capture details of the lived experience and individual pathways, they are not appropriate for inclusion in this review as there is no comparison group to determine what is unique about gang members when compared to non‐gang members.

In the studies, participants must have been recruited through random, stratified probability or total sampling. A study was eligible if it included participants recruited in an institutionalized or specialized setting (e.g. detention centre) if there was also a comparison group recruited from the community through random, stratified probability, or total sampling within both groups.

To be eligible for inclusion in a meta‐analysis, the study must have reported an effect size, or provided sufficient detail such that an effect size could be calculated.

#### Exclusion criteria

We excluded studies from countries that have not been categorized as low‐ or middle‐income by the World Bank for at least 50 per cent of the time since 1987.

### Search methods for identification of studies

#### Search terms

The search for eligible studies was conducted as part of a broader project systematically reviewing literature on conduct problems and youth crime in low‐ and middle‐income countries (Murray *et al*., 2013; Shenderovich *et al*., 2015) and alongside a systematic review on preventive interventions targeting youth gang violence in low‐ and middle‐income countries (Higginson *et al*., 2015). The search terms were broad enough to capture both the corpus of intervention studies and the corpus of studies for this present review, with further refinement occurring at the abstract and title screening stage for each review.

The search strategy was developed using the Cochrane Collaboration's Effective Practice and Organisation of Care Group search strategy for low‐ and middle‐income countries, combined with selected MeSH/DeCS terms and free text terms relating to conduct problems, crime and violence. To maximize sensitivity, no methodological filters were used. The full search strategy is listed in Appendix A.

The search strategy included published and unpublished literature with no date constraints. We did not place any language restrictions on the eligibility of documents; however our search was conducted in English, French, Chinese, Arabic, Russian, Spanish and Portuguese. The geographic location of studies was limited to countries located in a LMIC, defined according to the World Bank as low‐ or middle‐income at least 50 per cent of the time since 1987, when the recordings start (http://data.worldbank.org/about/country‐classifications/country‐and‐lending‐groups). The eligible countries included as low‐ and middle‐income are shown in Table 2.

**Table 2:** Eligible countries

**Existing states**	Afghanistan; Albania; Algeria; American Samoa; Angola; Antigua and Barbuda; Argentina; Armenia; Azerbaijan; Bangladesh; Barbados; Belarus; Belize; Benin; Bhutan; Bolivia; Bosnia and Herzegovina; Botswana; Brazil; Bulgaria; Burkina Faso; Burundi; Cambodia; Cameroon; Cape Verde; Central African Republic; Chad; Chile; China; Colombia; Comoros; Congo, Dem. Rep.; Congo, Rep.; Costa Rica; Côte d'Ivoire; Croatia; Cuba; Czech Republic; Djibouti; Dominica; Dominican Republic; Ecuador; Egypt, Arab Rep.; El Salvador; Equatorial Guinea; Eritrea; Estonia; Ethiopia; Fiji; Gabon; Gambia, The; Georgia; Ghana; Grenada; Guatemala; Guinea; Guinea‐Bissau; Guyana; Haiti; Honduras; Hungary; India; Indonesia; Iran, Islamic Rep.; Iraq; Jamaica; Jordan; Kazakhstan; Kenya; Kiribati; Korea, Dem. Rep.; Kosovo; Kyrgyz Republic; Lao PDR; Latvia; Lebanon; Lesotho; Liberia; Libya; Lithuania; Macedonia, FYR; Madagascar; Malawi; Malaysia; Maldives; Mali; Malta; Marshall Islands; Mauritania; Mauritius; Mexico; Micronesia; Moldova; Mongolia; Montenegro; Morocco; Mozambique; Myanmar; Namibia; Nepal; Nicaragua; Niger; Nigeria; Oman; Pakistan; Palau; Panama; Papua New Guinea; Paraguay; Peru; Philippines; Poland; Puerto Rico; Romania; Russian Federation; Rwanda; Samoa; São Tomé and Principe; Saudi Arabia; Senegal; Serbia; Seychelles; Sierra Leone; Slovak Republic; Solomon Islands; Somalia; South Africa; South Sudan; Sri Lanka; St. Kitts and Nevis; St. Lucia; St. Vincent and the Grenadines; Sudan; Suriname; Swaziland; Syrian Arab Republic; Tajikistan; Tanzania; Thailand; Timor‐Leste; Togo; Tonga; Trinidad and Tobago; Tunisia; Turkey; Turkmenistan; Tuvalu; Uganda; Ukraine; Uruguay; Uzbekistan; Vanuatu; Venezuela, RB; Vietnam; West Bank and Gaza; Yemen, Rep.; Zambia; Zimbabwe
**Former states**	Czechoslovakia; Gibraltar; Mayotte; Serbia and Montenegro; USSR; Yugoslavia

#### Search locations

We searched a wide range of electronic academic databases, international organisation databases, the websites of NGOs and other organisations. All locations were searched electronically. The searches were conducted in August and September 2013. The search locations are listed in Table 3.

**Table 3:** Search locations used in the English language systematic search (hosting platforms in parentheses)

Search locations
3ie Impact Evaluation Database (http://www.3ieimpact.org/evidence/impact‐evaluations/)
African Journal of Criminology and Justice Studies
Applied Social Sciences Index and Abstracts (ProQuest)
Asian Journal of Criminology
CINAHL (EBSCOhost)
Criminal Justice Abstracts (EBSCOHost)
Don M. Gottfredson Library of Criminal Justice Gray Literature Database
EconLit (EBSCOhost)
EMBASE (Ovid) 1974 to 2013 Week 35
ERIC (ProQuest)
IDEAS
Indian Journal of Criminology
International Bibliography of the Social Sciences (IBSS) (ProQuest)
International Juvenile Justice Observatory (IJJO) Documentation Center
JOLIS (IMF, World Bank and International Finance Corporation)
Journal of Gang Research
J‐PAL Evaluations Database (www.povertyactionlab.org/evaluations)
LILACS (Note: included Spanish and Portuguese search terms)
National Criminal Justice Reference Service Abstracts Database
NBER
Open Grey
Ovid MEDLINE(R) In‐Process and Other Non‐Indexed Citations and Ovid MEDLINE(R) 1946 to Present
Pakistani Journal of Criminology
ProQuest dissertations
PsycINFO (Ovid) 1967 to 2013
Russian Academy of Sciences Bibliographies (EBSCOHost)
SciELO (Note: included Spanish and Portuguese search terms)
Sociological Abstracts + Social Services Abstracts (ProQuest)
South African Crime Quarterly
South African Journal of Criminal Justice
Turkish Journal of Criminology
United Nations Development Programme website
Web of Science
WHO Collaborating Centre for Violence Prevention website (www.preventviolence.info)
World Bank

Table 4 shows the locations searched in languages other than English. Due to the nature of database interfaces, the searches in these databases were less complex. The outcome search terms were used and, where possible, the search terms for child and youth age groups.

**Table 4:** Search locations used in the non‐English language systematic search

**Language**	**Search locations**
Arabic	Index Medicus for the Eastern Mediterranean Region King Saud University Repository YU‐DSpace Repository Google Scholar
Chinese	China National Knowledge Infrastructure (CNKI) Wanfang Data Chongqinq VIP Information Company (CQVIP) BabelMeSH – National Institutes of Health Google Scholar
French	African Index Medicus (WHO) Afrolib (WHO) Global Health Library Revue de Médicine tropicale Refdoc Google Scholar
Russian	Elibrary.ru Google Scholar
Spanish and Portuguese	LILACS SciELO Google Scholar

Where possible we examined the full set of results from each search; however, in cases where the search produced an unmanageable number of results that could not be downloaded en masse, we screened the results online by page until the titles appeared irrelevant, based on the searcher's subjective judgement.

The non‐English language searches were conducted by a team of six researchers (four who spoke the search language as their first language, and two who spoke the search language fluently).

If dissertations were located that were potentially eligible for inclusion we contacted the author or their institution for a copy of the document. We conducted citation searches of eligible papers and citation harvesting from the references of included studies. We contacted members of the Advisory Group^1^ as well as other prominent scholars in the field to locate further studies that may not yet be published or located in our search. Any new literature of interest was obtained and assessed for eligibility.

### Data collection and analysis

#### Selection of studies

*Title and abstract screening*

The results of each search were imported into EndNote reference management software where the initial title and abstract screening took place.

A team of six trained research assistants used preliminary eligibility criteria to assess, on the basis of titles and abstracts, whether the studies returned from the systematic search were potentially eligible for inclusion in the systematic review. Due to the large number of studies identified in the wider English language search, and the specialized language skills required to screen the studies in the non‐English language search, each title and abstract was screened by only one author. One research assistant with native (or near‐native) language fluency screened all of the studies from their allocated language. One of the review authors (YS) screened all of the English language studies.

The initial title and abstract screening inclusion criteria were broad to take in all studies potentially eligible for reviews examining the predictors of youth conduct issues in LMICs.

The initial screening inclusion criteria were:

1. all participants are 10‐29 years old
2. located in a LMIC, defined according to the World Bank as lower or middle income at least 50 per cent of the time since 1987, when the recordings start
3. all participants recruited through random, stratified probability, or total sampling
4. included a community comparison group if the sample was selected from within prison or juvenile detention centres
5. assessed the association at the level of an individual between at least one specific predictor or correlate and a relevant outcome (including gang membership)
6. predictor or correlate is a single characteristic and does not include conglomerations of multiple constructs
7. longitudinal study, cross‐sectional study, or case‐control study: comparison of a group with the outcome (gang membership) and those without the outcome

Documents were excluded if the answer to any one of the criteria was unambiguously “No”, and were classified as potentially eligible otherwise. We erred on the side of inclusivity and only excluded studies where it was clear that these criteria were not met.

#### Full text eligibility screening

Once the initial title and abstract screening had taken place in EndNote, the group of studies that were potentially eligible was imported into SysReview, a Microsoft Access database designed for screening and coding of documents for systematic reviews. In order to narrow down the results of the initial search to the subset of studies that specifically focus on the predictors of involvement in youth gangs, different criteria were included at this second title and abstract screening stage.

A team of trained research assistants used a set of inclusion criteria to assess, on the basis of titles and abstracts, whether the studies returned from the systematic search were potentially eligible for inclusion in the systematic review. After training to ensure that each author adopted the same approach to screening, each document was screened by only one author. The training included a comprehensive briefing by the review manager, which included reading and discussing the protocol, after which each author independently screened a set of 20 studies. The results of the initial screening of the training corpus were then mediated by the review manager, in consultation with the full review team. Once the review team reached an agreement rate of above 95 per cent, the subsequent screening of each document was conducted by only one author. Any issues or questions that arose during coding were discussed amongst the review team and the review manager, and the review manager randomly checked screening decisions to ensure consistency.

The second title and abstract screening criteria were:

1. does not assess individual predictor or correlate of gang membership
2. not a duplicate source.

The full text eligibility screening criteria were:

1. reports on youth gangs
2. all participants are 10‐29 years old
3. located in a LMIC, defined according to the World Bank as lower or middle income at least 50 per cent of the time since 1987, when the recordings start
4. not a duplicate source
5. assessed the association at the level of an individual between at least one specific predictor or correlate and gang membership
6. predictor or correlate is a single characteristic
7. predictor or correlate is not a conglomerations of multiple constructs
8. eligible recruitment strategy
9. eligible study design.

The full‐text screening was done in two stages. If criteria 1–4 were all screened as “Yes” then the document proceeded to be screened on criteria 5–9. If any of the responses to criteria 1–4 were “No”, the document was immediately deemed ineligible and the responses to criteria 5–9 were not recorded.

Documents were eligible for detailed coding and inclusion in the meta‐analysis if and only if they were screened as “Yes” across all criteria (1–9), and were not considered eligible if they were screened as “No” for any of the criteria.

#### Data extraction

Two authors (KB and AH) used the SysReview database, along with a detailed coding companion document, to code in detail the documents that were eligible for inclusion in the meta‐analysis. The coding fields are shown in Appendix B, and included information on study information, sample characteristics, study quality, outcomes reported, and effect size data.

All coding conducted during training was checked by the review manager to ensure accuracy and consistency of information capture. For the final coding, all coding and effect size data were checked by a second author who was not blinded to the initial coding. Coding discrepancies were resolved by discussion between authors, in consultation with the review manager if required. For data from between‐groups studies, relevant data were imported into Stata to calculate standardized effect sizes and their standard errors.

We coded all predictors identified in the primary studies, and categorized them according to the framework of individual, peer group, family, school, and community factors, following the conceptualisation shown in Table 1.

Following Lipsey and Derzon (1999) and in line with the developmental framework of Howell and Egley (2005) and Thornberry and colleagues (2003) we planned to categorize factors according to the age of the respondent at the time of measurement, as different factors may have stronger influence during particular developmental periods; for example, if the absence of a male role model is a predictor of interest, it may have a stronger impact if measured at the age of 12 than it does at the age of 3. However, due to the low number of studies identified, this moderator analysis was not performed.

#### Assessment of risk of bias in included studies

We assessed risk of bias using a series of questions listed in the coding fields shown in Appendix B under Risk of Bias. The quality of each study was assessed by two authors, and the results of the two assessments mediated by the review manager, who was not blind to the original quality assessment. Coding discrepancies were resolved by discussion between authors, in consultation with the review manager. These items assessed the quality of the sampling, the measurement of items, and the timing of the measurements to identify whether the factor was indeed in existence before gang membership. When assessing risk of bias we did not allocate a score or index, as extreme failure in one area can be more serious than minor breaches of quality across multiple arenas. We did not exclude studies on the basis of risk of bias assessment, but planned to conduct moderator analysis to determine whether inclusion of studies with higher risk of bias impacts on the summary effect size. We presented the results of the assessments in a “traffic light” format (see de Vibe *et al*., 2012).

#### Effect size metric and calculations

For continuous predictors we used Cohen's *d* as the measure of effect size, and for binary predictors, we calculated a log odds ratio as the measure of effect size. We used Stata to calculate the effect sizes and convert between effect size types, to ensure that a common metric was used. Following Hawkins and colleagues (2000) we converted all effect sizes to the log odds ratio as a common effect size for synthesis and present results as the odds ratio, as it represents the amount of increased or decreased risk in an intuitive metric. Although converting different effect sizes to a common metric is imperfect, it is preferable to conducting multiple separate meta‐analyses (Borenstein *et al*., 2009).

The following formulae were used to generate effect sizes and their standard errors:

1. $\text{Log odds ratio=ln}\;(\frac{ad}{bc})$ where
  *a* = the number of youth gang members in the group *with* the characteristic of interest,
  *b* = the number of *non*‐youth gang members *with* the characteristic of interest,
  *c* = the number of youth gang members in the group *without* the characteristic of interest, and
  *d* = the number of *non*‐youth gang members *without* the characteristic of interest.
2. $Standard\;error\;(Log\;odds\;ratio\text{)=}\sqrt{\frac{1}{a}+\frac{1}{b}+\frac{1}{c}+\frac{1}{d}}$ where
  *a*, *b*, *c* and *d* are defined as in Equation 1.
3. $Cohen's\;d=\frac{\left(MeanT-MeanC\right)}{\sqrt{\frac{\left(nT-1)*(SDT^{2})+(nC-1)*(SDC^{2}\right)}{\left(nT+nC-2\right)}}}$ where
  - *MeanT* = the mean value of the characteristic among youth gang members (group *T*),
  - *MeanC* = the mean value of the characteristic among *non‐* youth gang members (group *C)*,
  - *nT* = the number of participants in group *T*,
  - *nC* = the number of participants in group *C*,
  - *SDT* = the standard deviation group *T*, and
  - *SDC* = the standard deviation of group *C*.

A further note on the calculation of Cohen's *d*: The majority of studies that reported data suitable for calculation of *d* reported the mean value of the characteristic for youth gang members and non‐youth gang members. Once converted to the log odds ratio, however, the resulting effect size was the equivalent of having compared the likelihood of youth gang membership between groups with and without the correlate, as the log odds ratio is symmetrical across conditions.

4. $Standard\;error\;d\text{=}\sqrt{\frac{nT+nC}{nT*nC}+\frac{d^{2}}{2*(nT+nC)}}$ where

*nT, nC* and *d* are defined as in equation 3.

The following formula were used to convert from Cohen's *d* to the log odds ratio:

5. $Log\;odds\;ratio\text{=}\frac{\pi d}{\sqrt{3}}$
6. $Standard\;error\;(Log\;odds\;ratio)=\sqrt{\frac{\pi^{2}*SEd^{2}}{3}}$

The following formula was used to calculate the standard error of the log odds ratio where a study reported an odds ratio and its confidence intervals only:

7. $SE\;(Log\;odds\;ratio)=\frac{\left(\ln(UCL)-\ln(OR)\right)}{1.96}$

#### Criteria for determination of independent findings

There were two issues of independence that needed to be addressed in this review. The first was that documents may have reported on multiple studies, which may in turn have reported on multiple predictors or outcomes. Documents were allowed to contribute multiple effect sizes; however, if more than one effect size was provided for a conceptually equivalent factor/outcome relationship, the effects were first synthesized using a random effects model with inverse variance weighting, and the pooled estimate was included in the analyses. In this way, the average effect of the conceptually equivalent factors was reported and included in the meta‐analyses. There was one study which was treated differently (Ohene, 2005), as it reported results for males and females separately, resulting in two independent effect sizes for each measure. These effects were not pooled prior to synthesis, as they were two independent sub‐samples, and were therefore treated as independent estimates.

The second issue of independence was that multiple documents might have reported on the same data. In these instances, we planned to identify which documents were related, and assess all sources in order to select an effect size, based on the completeness of the data and the risk of bias assessment of the studies. Two papers by Olate and colleagues (2011, 2012) both analysed the same dataset. The two papers largely reported on different factors; however, both papers reported on a conceptually equivalent factor/outcome relationship for violent delinquency and school attachment. For the analyses of these two relationships, the effect sizes from the two papers were pooled using a random effects model with inverse variance weighting, and this pooled estimate was included in the analyses.

#### Missing data

One eligible study (Moravcova, 2012) did not provide sufficient data to compute effect sizes, and so we have attempted to contact the author. This study will be included in future updates if effect size data becomes available.

#### Method of synthesis

We conducted a random‐effects meta‐analysis with inverse variance weighting to calculate an overall weighted mean effect estimate for each factor‐outcome association. We presented the results of the meta‐analyses in forest‐plots with 95 per cent confidence intervals.

We categorized each factor into the domains of individual, peer, family, school and community, and performed a meta‐analysis for each of these domains, using the summary effect sizes from each individual factor. We used forest plots with 95 per cent confidence intervals to present the results. We synthesized outcomes expected to be risk factors separately to those that we expected to be protective factors.

#### Assessment and investigation of heterogeneity

We tested for heterogeneity in the meta‐analyses using I^2^ and τ^2^ following Borenstein *et al*. (2009).

As all gang membership measures were self‐report, and all samples fell broadly into the 14 years and over age category, we did not perform moderator analyses on these factors. There were too few studies identified to reasonably perform moderator analysis by region and sampling strategy. All studies were peer reviewed publications. We performed moderator analyses on the domains of the predictors to identify the relative strength of the domains.

#### Sensitivity analyses

We had planned to conduct subgroup analyses in order to assess the impact of study quality and study design, including analyses of the effect of risk of bias, publication status, publication year, the use of partial regression coefficients versus bivariate correlation coefficients, and geographic level of analysis. Due to the low number of included studies in each analysis, these sensitivity analyses were not conducted.

#### Assessment of publication bias

We had planned to test and adjust for publication bias; however these tests were not conducted, due to the low number of included studies in each analysis (a maximum of six independent effect sizes in a meta‐analysis) and the fact that all the studies that we located were published studies. Consequently, we cannot assure the reader that the results are free of publication bias, particularly as only published studies were located.

#### Treatment of qualitative research

We did not use qualitative research to evaluate the factors associated with youth gang membership.

## Results

### Description of studies

#### Search and screening process

**Figure 2:**
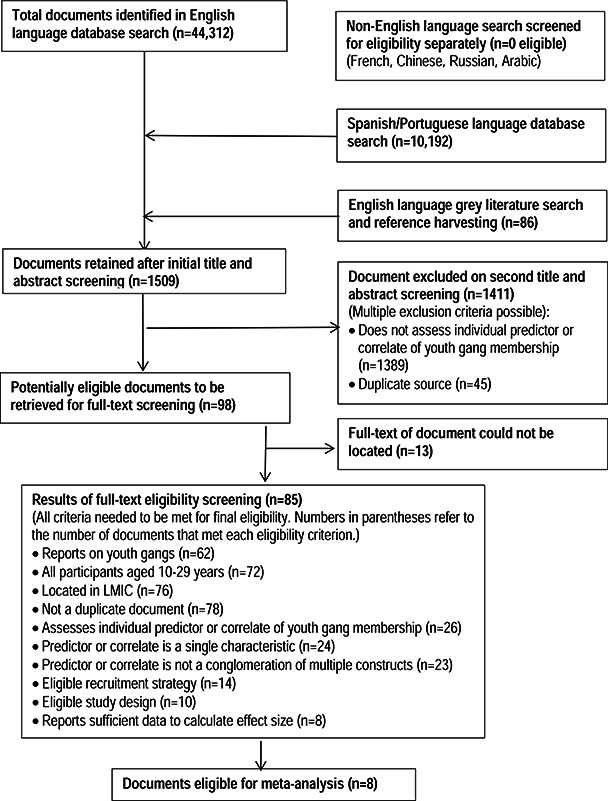
Flowchart of search and screening process

The results of the search and screening process are shown in Figure 2. The systematic search of English language sources yielded a total of 44,312 records, the Spanish and Portuguese search of databases yielded a further 10,192 records. The grey literature search and reference harvesting provided a further 86 documents, bringing the total set of documents to 54,590.

The searches in French, Russian, Arabic and Chinese did not generally allow easy export of results. Some databases allowed an export to Excel, whilst in others no direct export was possible and the search and screening processes were conducted simultaneously, in the manner of a grey literature search.

The titles and/or abstracts of documents were screened by a native speaker of the relevant language. The English language title and abstracts were screened by one author (YS). None of the records located in the French, Russian, Arabic or Chinese searches were deemed potentially eligible at the title and abstract screening stage. This first stage of title and abstract screening was to identify studies that would be eligible for a broader set of reviews, therefore the key screening criteria at this first stage were broader than those required to identify the subset of studies that examined the factors associated with youth gang membership.

After title and abstract screening, a set of 1509 studies was identified that were potentially eligible for inclusion in this review and each title and abstract was screened a second time. At this stage 1411 studies were excluded for one or more of the following criteria:does not assess individual factor associated with gang membership (n=1389); duplicate source (n=45).

The full text documents of the remaining 98 potentially eligible studies were searched for, and we succeeded in locating 85 documents. After full‐text screening, 89 studies were excluded on one or more criteria, and nine documents were deemed eligible for inclusion. One study did not report sufficient detail to allow the calculation of effect sizes, leaving eight documents included in the analyses.

#### Included studies

Eight studies were screened as eligible for inclusion in the analyses. These studies were:

- Abramovay, M., Jacob Waiselfisz, J., Coelho Andrade, C. and das Gracas Rua, M. (1999) *Gangs, crews, buddies and rappers: Youth violence and citizenship around the outskirts of Brasilia*. Brazil: UNESCO Brazil.
- Celbiş, O., Karaoğlu, L., Eğri, M., and Özdemir, B. (2012). Violence among high school students in Malatya: A prevalence study. *Turkish Journal of Medical Science*, 42(2), 343‐50.
- Katz, C. M., and Fox, A. M. (2010). Risk and protective factors associated with gang‐involved youth in Trinidad and Tobago. *Revista Panamericana de Salud Pública*, 27(3), 187‐202.
- Ohene, S. A. (2005). The clustering of risk behaviors among Caribbean youth. *Maternal and Child Health Journal*, 9(1), 91‐100.
- Olate, R., Salas‐Wright, C., and Vaughn, M.G. (2011). A cross‐national comparison of externalizing behaviors among high‐risk youth and youth gang members in Metropolitan Boston, Massachusetts, and San Salvador, El Salvador. *Victims and Offenders: An International Journal of Evidence‐based Research, Policy, and Practice*, 6 (4), pp. 356‐369. DOI: 10.1080/15564886.2011.607396.
- Olate, R., Salas‐Wright, C., and Vaughn, M. G. (2012). Predictors of violence and delinquency among high risk youth and youth gang members in San Salvador, El Salvador. *International Social Work*, 55(3), 383‐401.
- Pyrooz, D. C., and Decker, S. H. (2013). Delinquent behavior, violence, and gang involvement in China. *Journal of Quantitative Criminology*, 29:251‐272.
- Webb VJ, Ren L, Zhao J, He N, Marshall IH (2011) A comparative study of youth gangs in China and the United States: definition, offending and victimization. *Int Crim Justice Rev* 21:225–242

As noted in Section 3.4.1, two papers used the same data for their analyses, yet largely reported different measures (Olate *et al*., 2011; Olate *et al*., 2012). Effect sizes from these papers were pooled before inclusion in any meta‐analyses. One further eligible study is currently waiting for information from the author that may allow effect size calculations, and is therefore not included in the analyses at this time:

- Moravcova, E. (2012). Methodological aspects of gang membership: The case of the Czech Republic. *Acta Universitatis Carolinae Philosophica et Historica*, s. 69–83. ISSN 0567‐8293.

#### Excluded studies

The majority of studies screened at the full‐text stage did report on youth gangs (n=62), with the correct age of participants (n=72), and were located in LMICs (n=76). However, only 26 studies assessed the individual factors associated with youth gang membership, and only 10 included an eligible study design. There were seven duplicate documents identified at the full‐text screening stage. A list of the excluded studies and their reason/s for exclusion is included in Section 7.

During the final peer‐review process, two further studies were suggested by a reviewer. These documents were not readily available to the review team, but have been ordered and will be translated and assessed for eligibility in the next update.

### Characteristics of included studies

A brief summary of the eight included studies is reported below, and in Table 5.

**Table 5:** Characteristics of included studies

Study	Study objectives	Country	Methods of data collection	Data analysis	Correlates assessed
Abramovay et al. (1999) Chapter 3	Examine the prevalence, characteristics and behaviours of youth gangs.	Ceilandia, Planaltina and Samambaia, Brazil	A self‐report survey of 809 youth aged 15‐24 in selected residential blocks in 3 cities (data used in this review) Focus groups, interviews and case studies (qualitative data not used in this review)	For the purposes of this review we focus on the proportional differences between gang‐involved and non‐gang‐involved youth across the characteristics of interest. The authors used a mixed methods analysis, but no statistical analyses were reported. (Gang involved = present and past gang members)	Age Sex Work Education Live with both parents Violence in the family Engaged in violent situations Drug use
Celbis *et al*. (2012)	“To determine the prevalence of violence‐related behaviours on school property and to identify the predictors of youth violence among high school adolescents” (p. 343).	Malatya, Turkey	Self‐report questionnaire with 1175 students (747 males, 428 females). Cross‐sectional design, using stratified random sampling 6 urban high schools and 1 non‐urban high school in Malatya.	Authors reported the prevalence of violent behaviours (including gang membership) by key characteristics. A logistic regression model for violent behaviour was also reported but did not allow effect size calculation.	Sex Grade Family income School type, location Mother's education Success in school Exposure to violence (home, school, neighbourhood)
Katz and Fox (2010)	Explore prevalence of gang involvement and identify risk and protective factors associated with youth gang involvement.	Trinidad and Tobago	Data is drawn from the Trinidad and Tobago Youth Survey (TTYS), a self‐report survey completed by 2,206 students across 22 “high‐risk urban public schools” (892 males, 1,314 females).	Authors reported the prevalence of gang involvement by key characteristics, and a multinomial analysis of risk and protective factors for gang involvement (categories = never in gang, gang associate, former member, current member).	Gender, age, ethnicity Mobility, availability of handguns Commitment to school Parental attitudes Antisocial behaviour/peers Drug use/peer drug use Rewards and opportunities for prosocial behaviour Social skills, belief in moral order
Moravcová (2012)^2^	Assess the associations between different definitions of youth gangs and individual, social, and behavioural factors.	Czech Republic	The International Self‐Report Delinquency survey (waves 2 & 3) captured data from students in Grades 7‐9 at private and public schools (ages 12‐16; N = 6,707).	Authors performed a multinomial regression to predict an individual being categorised as a member of: a non‐gang group, a Eurogang defined gang, a self‐identified gang member, or a gang member identified on the Mokken scale. Authors reported the prevalence of 11 forms of delinquency across groups.	11 forms of delinquency Gender Age Self‐control index Personal morality index Family structure Family bonds index Truancy Risk behaviour index
Ohene *et al*. (2005)	Identify associations between risky behaviours and initiation of sexual activity among youth between 10‐18 years of age.	Antigua, Bahamas, Barbados, British Virgin Islands, Dominica, Grenada, Guyana, Jamaica and St Lucia (Caribbean)	Data is drawn from the Caribbean Youth Health Survey (random sampling), a self‐report survey completed by 15,695 school attending adolescents aged 10 to 18 (39% males, 61% females).	Relationships were assessed using odds ratios, stratified by gender and age group (full results were only reported stratified by gender). Statistical significance was reported. Survival analysis of factors associated with sexual initiation was also employed but did not allow effect size calculation.	Cigarette, alcohol, marijuana use Weapon carrying Runaway behaviour Skipping school Early sexual initiation
Olate *et al*., (2011)	A cross‐cultural comparison of high‐risk youth which examines the differences on externalizing risk behaviours in domains of school, work, sexual behaviours, substance abuse, and violence and delinquency.	Boston, USA and San Salvador, El Salvador	A cross‐sectional survey of members of two youth organisations in Boston (*N*= 374; 115 gang members) and one organisation in San Salvador covering four municipalities (*N*=208; 135 gang‐involved youth including 12 females). Mean age of El Salvadorian respondents was 20. (El Salvador data used in this review)	Authors reported the means and proportional differences between youth gang members and non‐members across five domains of externalising behaviours. T‐tests and chi‐square tests of significance were also reported.	School attendance measures Employed/employed FT Sexual behaviour Drug and alcohol use Measures of violence and delinquency (self‐report and official data)
Olate *et al*. (2012)	Examine the association between several risk factors and violence and delinquency in youth gang members and high‐risk youth.	San Salvador, El Salvador	Cross‐sectional survey using a non‐probability sample (*N*= 174) drawn from 10 urban and semi‐urban neighbourhoods within four Greater San Salvador Metropolitan municipalities. Administered by interviewers in individual or group format. Sample included 58 high‐risk non‐gang youth (13‐23 years; 36 male, 22 female) and 116 gang‐involved youth (13‐24 years; 106 male, 10 female). Appears to use the same data as Olate *et al*., 2011, so was treated as dependent.	Authors reported the means and proportional differences between youth gang members and non‐members across selected characteristics. T‐tests, chi‐square tests, and a correlation matrix were also reported. A logistic regression model predicting violence and delinquency was presented but did not allow effect size calculation.	Age, gender, parenthood status Measures of violence and delinquency Impulsivity, hope, empathy Unstable home, difficulty at home Expelled from school Delinquent peers Neighbourhood disorder Social support
Pyrooz and Decker (2013)	Examine the association between youth gang involvement and delinquent behaviour	Changzhi, China	Self‐report data collected from a school‐based convenience sample of 2,245 youth across six schools (mean age: 17.47; 1298 males, 865 females).	Authors reported the means and proportional differences between youth gang members and non‐members across selected characteristics. Two logistic regression models predicting offending were presented but did not allow effect size calculation.	Age, gender, minority status Broken home, parents’ education, rural Delinquency/delinquent peers Self‐control Family attachment School performance Parental monitoring Household strains
Webb *et al*. (2011)	A cross‐cultural comparison of the prevalence of gang involvement and the correlates of involvement for school‐age youth in China.	Hangzhou, China and a representative sample of five towns/cities in the United States.	The International Self‐Report Delinquency survey captured data from students in Grades 7‐9 at private and public schools (ages 12‐15; China *N* = 1,043; US N = 2,401).	Authors reported the proportional differences between youth gang members and non‐members across lifetime and last‐year prevalence of offending and victimisation. Chi‐square tests of significance were conducted.	Lifetime and last‐year prevalence of offending behaviour (drug, delinquent, criminal) Lifetime and last‐year prevalence of victimisation (robbery/extortion, assault, theft, bullying).

2 Data was not available to calculate effect sizes, therefore this study was not included in the meta‐analyses.

#### Population

The included studies were conducted in Turkey, Trinidad and Tobago, the Caribbean, El Salvador, China, Czech Republic, and Brazil. Two of the papers compared the samples taken in the low‐ or middle‐income country (El Salvador, China) with a sample taken from the USA; however the US sample is not used in this review.

The majority of the studies drew samples from schools (Celbis *et al*., 2012; Katz and Fox, 2010; Moravcová, 2012; Ohene *et al*., 2005; Pyrooz and Decker, 2013; Webb *et al*., 2011). The remaining studies drew samples from randomly selected residences (Abramovay *et al*., 1999) and a youth development organisation that dealt with high risk non‐gang youth as well as gang‐involved youth (Olate *et al*., 2011, 2012). School sampling means that the majority of the young people surveyed were under 18 years of age, with only the two non‐school samples (Abramovay *et al*., 1999; Olate *et al*., 2011, 2012) representing youth in their early twenties.

#### Gang membership

In each study, gang membership or involvement was self‐identified by the respondents. The precise categorisation of gang members differed across the studies. With the exception of Webb *et al*. (2011), the authors do not report whether they defined the term ‘gang’ to the participants prior to or whilst asking about gang membership

Three studies reported current gang membership (Celbis *et al*., 2012; Katz and Fox, 2010; Webb *et al*., 2011). Celbis *et al*. (2012) used the category of ‘gang member’ but did not report how they applied this category. Webb *et al*. (2011) used a three question index to categorize gang members: “Some people have a certain group of friends that they spend time with, doing things together, or just hanging out. Do you have a group of friends like that?”; “Do people in your group actually do illegal things together?”; and “‘Do you consider your group of friends a gang?”. If the young person responded yes to all items, they were categorized as a gang member. Katz and Fox (2010) used two questions (“Have you ever belonged to a gang;” and “Think of your four best friends. In the past year, how many of your best friends have been a member of a gang?”) to categorize young people into one of four groups: current gang members”; “former gang members”; “gang associates”; and “non‐gang members.” Both current and former gang members were asked further questions about the organizational structure of the gang as validation. For this review we only use the category of current gang members.

Five studies reported a combined measure of gang involvement as either current or former gang membership (Abramovay *et al*., 1999; Ohene *et al*., 2005; Olate *et al*., 2011, 2012; Pyrooz and Decker, 2013). Three studies used one question to categorize gang‐involved youth: Ohene *et al*. (2005) defined gang involvement as an affirmative answer to the question “Have you ever belonged to a gang?”, and Olate *et al*. (2011, 2012) used a similarly worded question “Have you ever participated in a gang?”. Abramovay *et al*. (1999) categorize both current and former gang members as ‘gang involved’ but did not report the categorisation process. Finally, Pyrooz and Decker (2013) used two questions to differentiate between non‐gang members, current gang members and former gang members: “Do you consider your group of friends to be a gang?” and “If you are not now, have you ever been in such a gang?”. The data available for inclusion in this review aggregates the current and former gang members as ‘gang involved’.

One study (Moravcová, 2012) used the International Self‐Report Delinquency study to test three different definitions of youth gang membership: the Eurogang definition, self‐identification, and the Mokken scale. A respondent was identified as a gang member using the Eurogang definition if they answered yes to these four questions: 1. Does this group spend a lot of time together in public places like the park, the street, shopping areas, or the neighborhood? 2. How long has this group existed? 3. Is doing illegal things (against the law) accepted by or okay for your group? 4. Do people in your group actually do illegal things (against the law) together?”A respondent was identified as a self‐identified gang member if they responded yes to the question “Do you consider your group of friends to be a gang?”Finally, a respondent was identified as a gang member using the Mokken scale if they answered yes to each of the Eurogang questions and the self‐identification question.

#### Study design and analysis

Only five studies aimed to identify associations between gang membership and risk or protective factors (Abramovay *et al*., 1999; Katz and Fox, 2010; Moravcová, 2012; Pyrooz and Decker, 2013; Webb *et al*., 2011). The remainder of the studies focused on identifying the factors associated with youth violence or other externalising behaviours (Celbis *et al*., 2012; Olate *et al*., 2011, 2012), or early sexual activity (Ohene *et al*., 2005), and used gang membership as a correlate. Therefore, these latter four studies did report sufficient data to allow effect sizes to be calculated for the associations with youth gang membership.

The majority of studies reported data as either the mean or proportional differences between gang‐involved and non‐gang‐involved youth across the characteristics of interest (Abramovay *et al*., 1999; Celbis *et al*., 2012; Katz and Fox, 2010; Moravcová, 2012; Olate *et al*., 2011, 2012; Pyrooz and Decker, 2013; Webb *et al*., 2011). Ohene *et al*. (2005) reported odds ratios and Olate *et al*. (2012) reported a correlation matrix. Moravcová (2012) conducted a multinomial regression model to assess the associations of individual, social, and behavioural factors with the different gang definitions.

This is not to say that the overall analyses in the studies were of low quality; rather, that the statistical analyses conducted in the papers were not always focused on explaining gang membership. Therefore the data that were available to extract were largely bivariate relationships reported in descriptive statistics. The studies also conducted qualitative analyses (Abramovay *et al*., 1999), logistic regression analyses (Celbis *et al*., 2012; Olate *et al*., 2012; Pyrooz and Decker, 2013), multinomial regression (Katz and Fox, 2010), survival analyses (Ohene *et al*., 2005), t‐tests and chi‐square tests of significance (Olate *et al*., 2011, 2012; Webb *et al*., 2011).

### Risk of bias in included studies

Each of the included studies was assessed for study quality using the eight questions reported in Table 6 below. Each item is answered Yes (Y), No (N) or Unclear (UC).

**Table 6:** Risk of bias in included studies

**Study Name**	**Abramovay *et al*., 1999**	**Celbis *et al*., 2012**	Katz and Fox, 2010	**Moravcová, 2012**	**Ohene, 2005**	**Olate *et al*., 2011**	**Olate *et al*., 2012**	Pyrooz and Decker, 2013	**Webb *et al*., 2011**
Study population criteria: Does the document describe the source population in replicable detail?	Y	Y	Y	Y	N	Y	Y	Y	Y
Study population criteria: Does the document list all inclusion and exclusion criteria for participation?	Y	N	Y	Y	N	N	N	Y	N
Prospective study: Was the study prospective (ie the sample was selected prior to the onset of gang membership)?	N	N	N	N	N	N	N	N	N
Outcome descriptor: Were the gang membership criteria described in replicable detail?	N	N	Y	Y	Y	Y	Y	Y	Y
Predictor/correlate description: Were all predictors/correlates described in replicable detail?	N	N	Y	N	Y	N	Y	Y	Y
Predictor/correlate validity: Were all measures of the predictors/correlates based on a validated measure?	N	N	Y	N	N	N	UC	N	N
Predictor/correlate timing: Were all predictors/correlates either measured before the onset of gang membership or measured retrospectively to a time prior to gang membership?	N	N	N	N	N	N	N	N	N
Selective analysis reporting: was the study free from analysis reporting bias?	UC	UC	UC	UC	UC	UC	UC	UC	UC

The source populations were described in replicable detail in eight out of nine studies; however the population inclusion and exclusion criteria were not fully listed in five of the nine studies. There is also an issue with regards to sampling. As discussed in section 4.2, the majority of the studies were based on school samples (Celbis *et al*., 2012; Katz and Fox, 2010; Moravcová, 2012; Ohene *et al*., 2005; Pyrooz and Decker, 2013; Webb *et al*., 2011). Each of the samples included both gang‐involved and non‐gang‐involved youth; however these sampling approaches may limit the generalisability of the results. Sampling from schools ensures that only those young people who are still engaged in school will be identified. School sampling also limits the age range under consideration, restricting participants to those under 18, and preventing generalizability to young adults.

None of the studies used a prospective sample. Combined with the fact that none of the studies used a retrospective approach to questioning, this means that none of the studies can truly speak to the predictors of youth gang membership. Rather, the included studies can only speak to the correlates of gang membership, as each study was cross‐sectional in design. This introduces a large risk of bias, and it is not at all possible to make causal attributions with these data, except for in the case of generally time‐invariant variables such as sex.

As described in section 4.2.2, while the majority of studies were clear in how they categorized gang membership or gang involvement, two studies gave no detail (Abramovay *et al*., 1999; Celbis *et al*., 2012). The main issue with how gang membership was classified in these studies is that the term ‘gang’ was overwhelmingly undefined (with the exception of Webb *et al*. (2011), Katz and Fox (2010) and Moravcová (2012) who asked follow‐up questions to validate gang status). One concern is that a lack of a guiding definition may mean that, in some instances, the investigators and the participants may have a different understanding of what a gang is, particularly when the research is conducted cross‐culturally. Whilst, Pyrooz and Decker (2013) specifically examined this question and concluded that self‐nomination was feasible in the Chinese context, the lack of specificity may pose an issue in other cultural contexts. In addition, the studies were split between those that examined the correlates of current youth gang membership, and those that examined the correlates of both current and past gang membership. As the number of studies was very small, we have chosen to collapse these categories in our syntheses; however, we acknowledge that combining these categories may introduce bias.

Five of the studies described the correlates in replicable detail, however four of the studies did not adequately define the correlate. This introduces difficulties for both synthesis and interpretation. The majority of correlates were not based on validated measures, but for those that were well described there was face validity. Given the types of correlates reported, the lack of validated measures is not problematic for this review; however, future studies that examine psychological correlates in particular would benefit from using validated measures.

Selective analysis may have been an issue within these studies as it was not clear if the analyses reported were designed a priori or post hoc. Without further information we cannot evaluate the impact that this may have on the results.

As discussed in section 4.2.3, not all of the included studies specifically aimed to assess the correlates or predictors of youth gang membership. Consequently, the data for this review have been mostly drawn from bivariate relationships reported as descriptive statistics. Whilst this simplifies the interpretation of the associations, it introduces bias, as the non‐gang samples are not matched to the gang samples, so the associations seen may be due to some other selection effects.

In sum, whilst these studies were each of good quality, there are some key considerations that may introduce bias and limit the generalizability of the results of this review. We therefore urge caution in interpretation.

### Synthesis of results

The synthesis is based on the eight eligible documents where we were able to extract the necessary data to calculate effect sizes. Moravcová (2012) was not able to be included due to a lack of detail. There were a total of 189 outcomes identified, classified as either a predictor or correlate, across five domains. Table 7 shows the distribution of the 189 outcomes by domain and classification of the factor.

**Table 7:** Distribution of factors by domain (*=classified as predictor; all other factors are classified as correlates)

	**Individual**	**Peer**	**Family**	**School**	**Community**	**Total**
Age	4	0	0	0	0	4
Alcohol/soft drugs	15	0	0	0	0	15
Delinquency	7	0	0	0	0	7
Delinquent peers	0	5	0	0	0	5
Employment	5	0	0	0	0	5
Ethnicity*	1	0	0	0	0	1
Exposure to violence	0	0	5	5	5	15
Family income	0	0	3	0	0	3
Gender*	5	0	0	0	0	5
Geography	0	0	0	2	1	3
Hard drugs	9	0	0	0	0	9
Home environment	1	0	10	0	1	12
Level of education	0	0	0	8	0	8
Neighbourhood environment	0	0	0	0	4	4
Non‐violent delinquency	20	0	0	0	0	20
Parental attitudes	0	0	2	0	0	2
Parental education	0	0	5	0	0	5
Prosocial peers	0	2	0	0	0	2
Psychological	5	1	0	0	0	6
School attachment	0	0	0	8	0	8
School environment	0	0	0	9	0	9
School performance	0	0	0	7	0	7
Sexual behaviour	5	0	0	0	0	5
Victimisation	4	0	0	0	0	4
Violent delinquency	25	0	0	0	0	25
Total	106	8	25	39	11	189

Due to the presence of zero counts in some cross‐tabulations of data, effect sizes could not be extracted for 26 of the factor‐outcome pairs. The remaining 163 outcomes cannot all be considered independent, as in many cases one study contributes multiple effect sizes that can be applied to the one analysis. As explained in section 3.4.2, in these instances the non‐independent effects are first synthesized before being included in the final analyses. This process resulted in 85 independent effect sizes that were synthesized across 37 separate analyses. Whilst 85 effect sizes may appear to be a large number, due to the large number of dependent effect sizes and separate analyses, there is only a maximum of six independent effect sizes contributing to any one analysis. Forty of the 85 independent effect sizes relate to the individual domain, 17 to the family domain, 17 to the school domain, seven to the community domain, and four to the peer domain.

The classification of a factor as either a predictor or a correlate was made according to the method described in section 3.1.1.3, whereby only factors that were time‐invariant or very clearly measured prior to the onset of gang membership, were classified as predictors. All other factors, including those where the timing of the measure was ambiguous, were classified as correlates. Only gender and ethnicity are classified as predictive factors, and all other factors are classified as correlates.

#### Notes for interpreting forest plots

Where there is more than one effect size reporting on a conceptually similar outcome, the results are presented as a forest plot, showing odds ratios and 95 per cent confidence intervals for each of the studies, as well as for the overall summary (shown as a diamond). Where only one study contributes effect sizes, the results are discussed in text, but no forest plot is presented.

Where the summary confidence intervals do not overlap one (the vertical line on the forest plot), there is a statistically significant association between the factor of interest and youth gang membership.

The horizontal axis is marked “less gang” for values less than one, and “more gang” for values greater than one, an abbreviation for “reduced odds of gang membership” and “increased odds of gang membership” respectively.

If the study confidence intervals are much wider than is practical to show on the forest plot, the confidence interval is truncated and marked with an arrow.

Where a study provides more than one conceptually similar effect size, as discussed in Section 3.4.2, those effects are first synthesized and the summary data is used in any subsequent meta‐analysis. Where this is the case, the number of effect sizes that have undergone preliminary synthesis is indicated against that study on the forest plot.

#### Individual correlates

The included studies provided effect sizes to synthesize the associations between gang membership and 11 individual factors. Apart from gender and ethnicity, which were classified as predictors, none of these factors were time‐invariant or measured prior to onset of gang membership, so it must be cautioned that these associations are not causal.

##### Age

Four studies report on the association between age and gang membership. The studies measure the current age of the participant, not age at onset of gang membership, and consequently age is classified as a correlate.

Two studies (Abramovay et al., 1999; Webb et al., 2011) found a negative but not statistically significant relationship between age and gang membership, whilst the other two studies (Pyrooz and Decker, 2013; Olate et al., 2012) found a positive but not statistically significant relationship. Three of the studies measure age in years, either presenting the mean and standard deviation (Pyrooz and Decker, 2013; Olate et al., 2011) or a frequency table of age by gang membership with categories from 12 years to 16+ years (Webb et al., 2011). One study (Abramovay et al., 1999) measured age with two categories, comparing ages 18 to 24 to the proportion who are aged 15 to 17 years. The pooled estimate suggest a non‐significant relationship (OR: 1.06, LCL: 0.74, UCL: 1.50). There is no significant heterogeneity amongst the studies (I2: 55%, p=0.083; τ2=0.068). Although the results of Moravcová (2012) could not be included in the analyses, their study also showed no clear association between age and gang membership.

**Figure 3:**
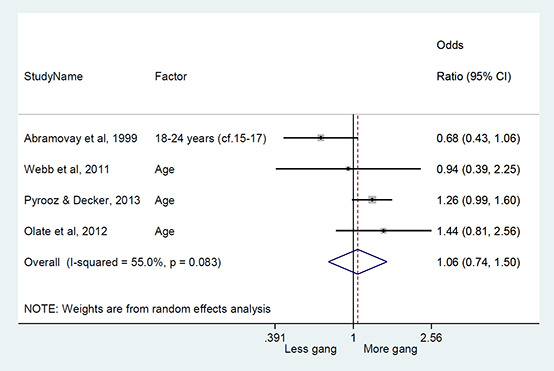
Age

##### Gender

Five studies examined the relationship between gender and gang membership. As gender is generally considered to be a time‐invariant factor, it is classified as a predictor for the purposes of this review, and male gender is categorized as a risk factor.

One study (Webb et al., 2011) found a non‐significant positive association between gang membership and being male, whilst the other four studies found a significant positive association (Pyrooz and Decker, 2013; Celbis et al., 2012; Olate et al., 2012; Abamovay et al., 1999). There is however significant heterogeneity of effects, with a much stronger effect (a six‐fold increase in odds) found in Olate et al., 2012 than in the other studies, where the increase in the odds of gang membership for males ranges from 33 per cent to 91 per cent (I2: 62.2%, p=0.032; τ2=0.124). The pooled estimate suggests an overall positive association between the male gender and gang membership, significant at the 95 per cent confidence level (OR: 2.04, LCL: 1.35, UCL: 3.08). Overall, males have twice the odds of reporting gang membership than females. Although the results of Moravcová (2012) could not be included in the analyses, their study showed no clear association between the measures of gang membership and gender.

**Figure 4:**
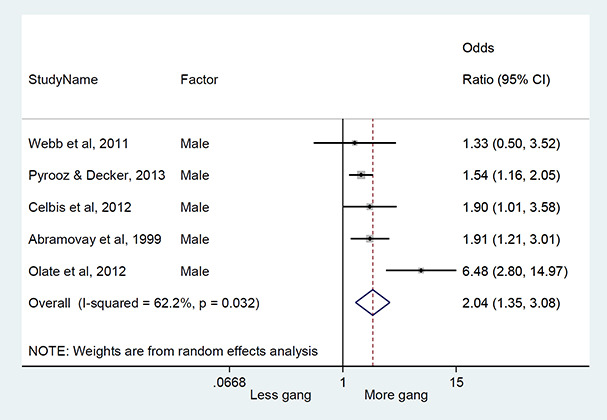
Gender

##### Ethnicity

One study examined the association between minority ethnicity and youth gang membership in a Chinese sample (Pyrooz and Decker, 2013). As this is a time‐invariant factor, minority ethnicity is also classified as a predictor. There was no significant association between Han ethnicity and youth gang membership in this study (OR: 2.05, LCL: 0.76, UCL: 5.55).

Employment

Two studies contributed multiple measures of employment (Abramovay et al., 1999; Olate et al., 2011). As these estimates come from the same sample, they have been pooled before including in the final synthesis across studies. Abramovay et al. (1999) measured employment in three ways, none of which were individually statistically significant: formal work documentation (negative relationship); work experience (positive relationship); and presently employed (positive relationship). The pooled estimate of the relationship between employment and gang membership for these three measures is positive, but not statistically significant. Olate et al. (2011) measured employment in two ways, both of which were individually positive and statistically significant relationships: employed; and employed full time.

The overall estimate for the two studies’ pooled measures of employment and gang membership is positive and statistically significant (OR: 1.97, LCL: 1.07, UCL: 3.63). There is no significant heterogeneity in the association between employment and gang membership (I2: 9.5%, p<0.293; τ2=0.019). Overall, these indices of employment almost double the odds of youth gang membership, and therefore, perhaps counterintuitively, appear to be risk factors for gang membership.

Employment, of course, need not be legitimate (as Abramovay's negative relationship between formal work documentation and gang membership illustrates). An alternative explanation is that employment, and full‐time employment in particular, is more likely to be available to those who are no longer in full‐time education. The relationship between employment and gang membership may therefore be indirect and mediated by poor school attendance or attachment, which is a significant correlate of gang membership (see Section 4.3.6.5).

**Figure 5:**
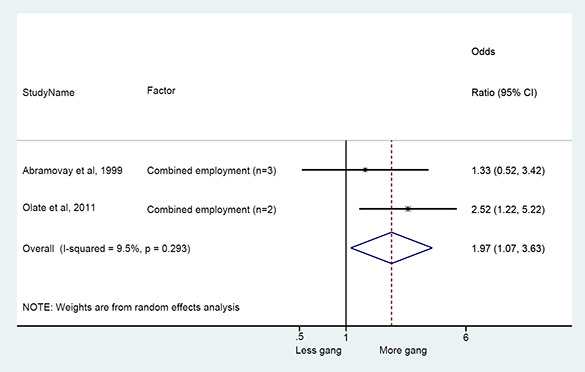
Employment

##### Delinquency

Three studies examined a total of six measures of general delinquency (Katz and Fox, 2010; Olate et al., 2011; Pyrooz and Decker, 2013). Delinquency is treated as a correlate, as it is generally measured concurrently with gang membership. In the one estimate of the association between early initiation of antisocial behaviour and youth gang membership (Katz and Fox, 2010), the study asks the age of initiation, but does not specify whether this early initiation occurs prior to, or concurrently with, the onset of gang membership. Therefore this effect is also treated as a correlate.

Katz and Fox (2010) measure early intitiation of antisocial behaviour, and Pyrooz and Decker (2013) report a delinquency index. Olate et al. (2011) report four measures of delinquent behaviour: arrest, number of arrests, legal problems, and a delinquency index. All six individual estimates suggest a positive and statistially significant relationship.

The overall estimate suggests delinquency is associated with more than three and a half times the odds of reporting youth gang membership (OR: 3.65, LCL: 1.89, UCL: 7.04). There is significant heterogeneity between studies (I2: 91.3%, p<0.001; τ2=0.306) with early initiation of antisocial behaviour showing a weaker association with gang membership than the other measures of delinquency.

Although the results of Moravcová (2012) could not be included in the analyses as effect sizes could not be calculated, non‐gang involved youth had lower prevalence than gang‐involved youth for each of 11 forms of delinquency (shoplifting, vandalism, group fighting, carrying weapon, drug dealing, assault, robbery/extortion, car break, burglary, car theft, and bicycle theft). Gang‐involved youth also showed higher levels of leisure time risky behavior than non‐gang involved groups, regardless of gang definition. There was no clear effect of gang membership on truancy behaviours, with truancy only associated with Eurogang definition but not self‐identification or Mokken scale.

**Figure 6:**
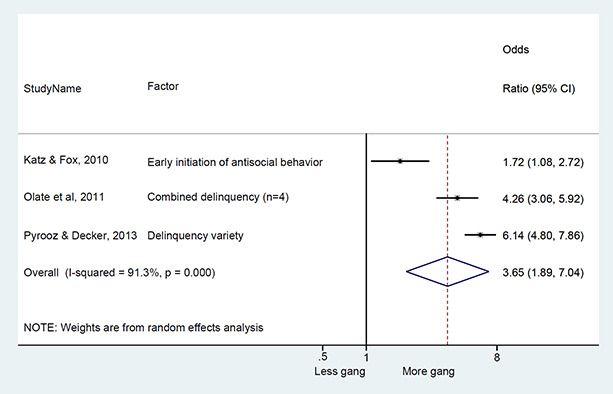
Delinquency

##### Violent delinquency

Six documents provided a total of 17 estimates of the association between various forms of violent delinquency and gang membership (Abramovay et al., 1999; Ohene, 2005; Olate et al., 2011, 2012; Pyrooz and Decker, 2013; Webb et al., 2011). Abramovay (1999) reported seven measures that were categorized as involvement in violence: sexual aggression, fights in traffic jams, robberies/mugging, fights over lovers, frisk, physical aggression, and other problems with police. All measures showed a positive relationship with gang membership, and all but the first three listed were statistically significant. Ohene (2005) reported two measures of violence: weapon carrying for males, and separately for females. Both were statistically significant positive effects. Olate et al. reported two measures in their 2011 paper: a physical aggression index and violence, and three measures in their 2012 paper: carrying weapons, attack with a weapon, and hitting someone. All were statistically significant positive effects. Pyrooz and Decker (2013) reported one measure of violent delinquency, which was a significant positive effect. Each relationship was positive and all but three of these effects were individually significant. Webb et al. (2011) reported two measures: lifetime prevalence of group fighting (positive but not significant); and lifetime prevalence of weapon carrying (positive and significant). The estimates from each study other than Ohene (2005) were synthesized prior to being included in the final meta‐analysis.

The overall estimate demonstrates that self‐reported violent delinquency is associated with an almost six‐fold increased odds of youth gang membership (OR: 5.83, LCL: 5.12, UCL: 6.63), and there is no significant heterogeneity amongst the effects (I2: 48.1%, p=0.086; τ2=0.010).

**Figure 7:**
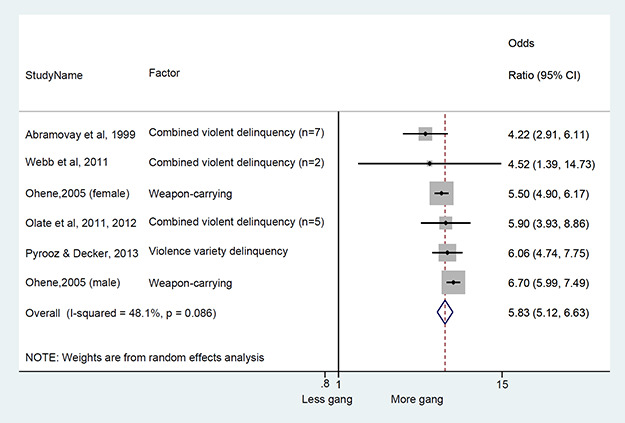
Violent delinquency

##### Non‐violent delinquency

Three studies provide estimates for nine measures of non‐violent delinquency (Olate et al., 2012; Pyrooz and Decker, 2013; Webb et al., 2011). Olate et al. (2012) report a positive association between gang membersip and three different measures of non‐violent delinquency (selling marijuana; buying or selling stolen items; and stealing a valuable item) Pyrooz and Decker (2013) report a positive association with a general measure of non‐violent delinquency. Finally, Webb et al. (2011) also find a positive association between gang membership and five measures of non‐violent delinquency (lifetime prevalence of shoplifting; vandalism; pick pocketing; and burglary, and one measure of last year prevalence: vandalism). Multiple estimates from one study were synthesized before being combined in an overall meta‐analysis.

The overall estimate suggests that youth who report non‐violent delinquency have more than four and a half times the odds of also reporting youth gang membership. Non‐violent delinquency is significantly associated with gang membership (OR: 4.67, LCL: 3.80, UCL: 5.76), and there is no significant heterogeneity across studies (I2: 0.0%, p=0.502; τ2=0.000).

**Figure 8:**
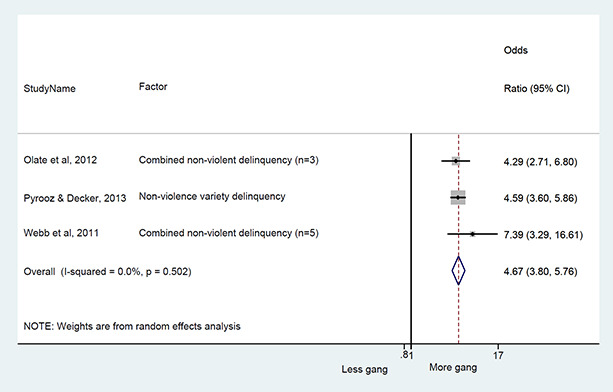
Non‐violent delinquency

##### Psychological factors

Three of the included studies report psychological risk or protective factors that have opposing relationships with gang membership, each of which is internally homogeneous. Three studies contributed estimates for measures of psychological factors (Katz and Fox, 2010; Olate et al., 2012; Pyrooz and Decker, 2013). Katz and Fox (2010) report one measure: belief in a moral order, which has a significant negative association with youth gang membership and is conceptualized as a protective factor. Olate et al. (2012) report three measures: impulsivity; empathy; and future orientation (or hope). Impulsivity shows a significant positive association and is conceptualized as a risk factor. Empathy shows a significant negative association and future orientation show a non‐significant negative association, and both are conceptualized as protective factors. Pyrooz and Decker (2013) report a measure of low self‐control, which shows a significant positive association and is conceptualized as a risk factor. To avoid issues of independence, we first calculated overall estimate from the two protective factors contributed by Olate et al., 2012 (empathy and future orientation) before including in the meta‐analysis.

Youth who report low self‐control or impulsivity have approximately 50 per centgreater odds of also reporting youth gang membership than those without these psychological risk factors (OR: 1.51, LCL: 1.21, UCL: 1.88). There is no significant heterogeneity between these studies (I2: 0.0%, p=0.520; τ2=0.000). Conversely, youth who report empathy, future orientation, or a belief in the moral order have approximately 40 per cent lower odds of reporting youth gang membership than those without these psychological protective factors (OR: 0.57, LCL 0.42, UCL 0.77). Again, there is no significant heterogeneity between these studies (I2: 0.0%, p=0.622; τ2=0.000).

Although the results of Moravcová (2012) could not be included in the analyses, gang‐involved youth showed lower self‐control and weaker personal morality than non‐gang involved youth, across all three definitions of gang involvement.

**Figure 9:**
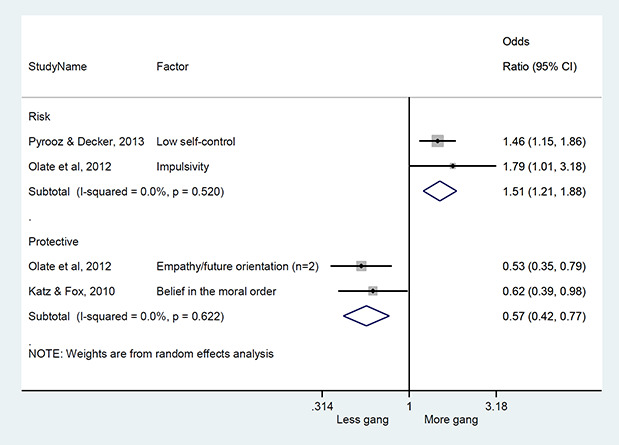
Psychological factors

##### Victimisation

One study (Webb et al., 2011) provided estimates of four different measures of victimization: robbery/extortion; bullying; theft; and assault. Once again, this factor is treated as a correlate as the studies do not explicitly measure victimization prior to onset of youth gang membership. Robbery/extortion victimisation showed a significant positive effect, theft and assault victimization showed a non‐significant positive effect, and bullying victimisation showed no effect. Combining the estimates from this study suggests more than twice the odds of gang membership for those that report victimization, compared to those who do not (OR: 2.39, LCL: 1.00, UCL: 5.71). There was no significant heterogeneity amongst the effects (I2: 31.1%, p=0.226; τ2=0.248).

##### Sexual behaviour

There were two studies that provided a total of five estimates of association between different sexual behaviours and gang membership. We classify these as correlates, as there is no clear reporting of sexual behaviour prior to onset of youth gang membership. Three of these effects can be considered risk factors. Ohene et al. (2005) report two measures of sexual behaviour risk factors: early sexual initiation reported separately for males and females, both of which show a significant positive association with youth gang membership. Olate et al. (2011) report one measure: having engaged in sexual intercourse, which also shows a significant positive association. Overall, the risk factors show a significant relationship with gang membership, with the pooled effect suggesting sexual activity and early sexual initiation is associated with triple the odds of gang membership (OR: 3.29, LCL: 3.00, UCL: 3.62), with no significant heterogeneity between studies (I2: 18.5%, p=0.293; τ2=0.001).

**Figure 10:**
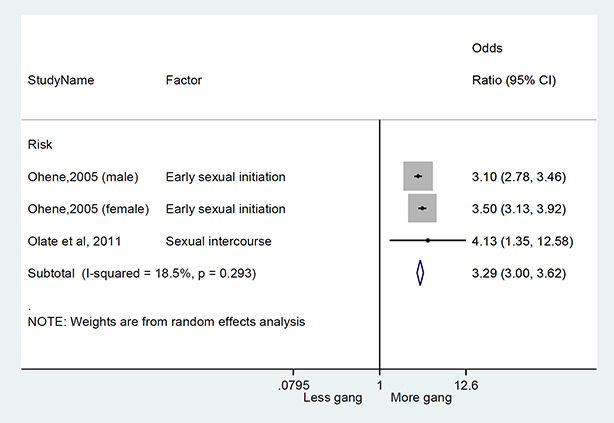
Sexual behaviour risk factors

Two sexual behaviour protective factors were reported by Olate et al. (2011): age at first sexual intercourse; and condom use. These measures are considered protective factors as they may be to be associated with less impulsivity. Age at first intercourse shows a significant negative relationship, whilst condom use shows no association. Combining these two estimates does not show an association with youth gang membership (OR: 0.67, LCL: 0.28, UCL: 1.60), although there is significant heterogeneity between these two measures (I2:80.2%, p=0.025; τ2=0.318).

##### Alcohol and soft drugs

Four studies provided data on the use of alcohol and soft drugs, all of which individually showed a significant positive association between the use of alcohol, marijuana and tobacco, and youth gang membership (Ohene, 2005 (data reported separately for males and females); Olate et al., 2011; Katz and Fox, 2010; Abramovay et al., 1999). The overall pooled effect suggests alcohol and soft drugs are associated with more than triple the odds of gang membership (OR: 3.23, LCL: 2.57, UCL: 4.07). There results of the studies are highly homogeneous (I2: 0.0%, p=0.735; τ2=0.000).

**Figure 11:**
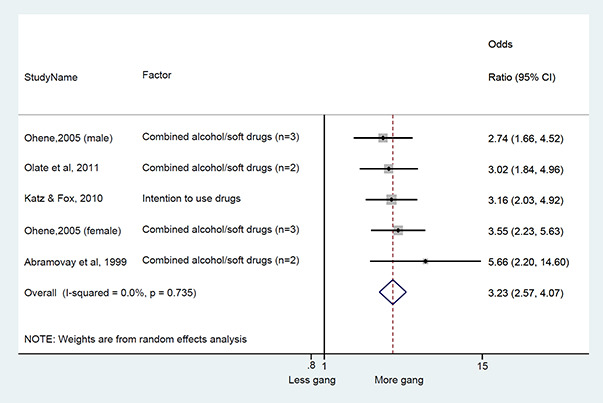
Alcohol and soft drugs

One study (Olate et al., 2011) reported two measures that could be considered protective: the age when the respondent first had five alcoholic drinks, and the age they first used marijuana. Both individual effects are negative, although only alcohol is significantly so, with youth gang members beginning to drink approximately eight months earlier than non‐gang members. The pooled effect is a non‐significant negative relationship (OR: 0.69, LCL: 0.42, UCL: 1.13). There results of the studies are heterogeneous (I2: 45%, p=0.178; τ2=0.057).

##### Hard drugs

One study (Abramovay et al., 1999) reported effect sizes for the relationship between youth gang membership and five types of hard drugs: crack, pills, glue, cocaine, and merla. Each drug had an individually positive relationship with gang membership, and for all except crack this was a significant association. The pooled effect is highly homogeneous (I2: 0.0%, p=0.659; τ2=0.000), showing that there was a consistent association across all surveyed drug types. The pooled effect is also stronger than that seen with alcohol and soft drugs, with hard drugs being associated with almost five times the odds of youth gang membership (OR: 4.80, LCL: 3.06, UCL: 7.52).

##### Peer correlates

Data were available for two constructs: delinquent peers and pro‐social peers. Each of these factors were classified as correlates.

##### Delinquent peers

Three studies measured the delinquency of peers (Katz and Fox, 2010; Olate et al., 2012); Pyrooz and Decker, 2013).Katz and Fox (2010) reported three measures of peer delinquency: peer alcohol use, peer drug use, and peer antisocial behaviour. Peer alcohol use was positively, but not significantly, associated with youth gang membership, whilst peer drug use and antisocial behaviour were both positively and significantly associated with youth gang membership, as was the pooled effect from this study. Olate et al. (2012) and Pyrooz and Decker (2013) both reported general measures of peer delinquency, and both relationships were positive and statistically significant. Overall, association with delinquent peers corresponds to almost four times the odds of reporting youth gang membership (OR: 3.96, LCL: 1.19, UCL: 13.20). These estimates are also highly heterogeneous (I2: 96.3%, p<0.001; τ2=1.073).

**Figure 12:**
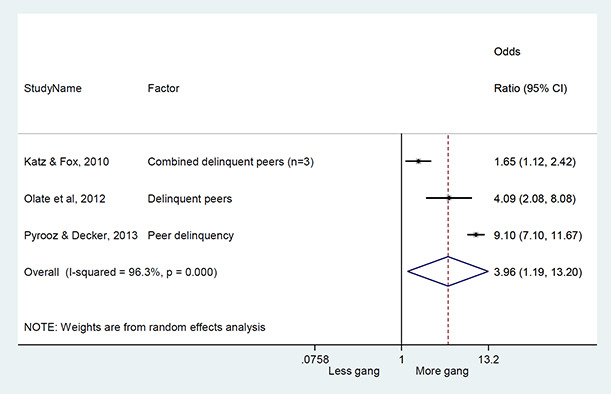
Delinquent peers

##### Prosocial peers

In contrast to the positive association between delinquent peers and youth gang membership, there is no significant protection offered by associating with prosocial peers. Katz and Fox (2010) examined two different measures of prosocial peers: interaction with prosocial peers, and peer reward for prosocial involvement. Neither measure showed an individually significant relationship with gang membership, nor was there any significant heterogeneity between their effects (I2: 0.0%, p=0.476; τ2=0.000). The pooled effect is also not significantly different from an odds ratio of 1 (OR: 1.05, LCL: 0.77, UCL: 1.44).

##### Family correlates

This section includes meta‐analyses on the risk and protective factors relating to the family environment, family income, parental attitudes, parental education, and exposure to violence in the home. Each of these factors is classified as a correlate, as the studies measure the factors concurrently with youth gang membership. Whilst it may feel intuitive to assume that family factors precede a young person's gang membership, without at least retrospective reporting, it is not reasonable to assume that family factors are stable or time invariant. Indeed, it is quite possible that family factors may change as a response to youth gang onset, resulting in seemingly paradoxical effects; for example, a family may actively become more supportive after they notice a child getting in trouble with the law.

##### Family environment

Four studies provided information on the home environment and its association with youth gang membership (Olate et al., 2012; Katz and Fox, 2010; Pyrooz and Decker, 2013; Abramovay et al., 1999). These factors were classified into risk factors (for example, an unstable home, residential mobility, running away from home) and protective factors (living with both parents, opportunities for pro‐social involvement in the family, and family attachment). Both risk and protective factors are associated with youth gang membership in the anticipated direction, although the estimates of the risk factors is more than twice that of the protective factors (Risk OR: 1.92, LCL: 1.33, UCL: 2.79; Protective OR: 0.76, LCL: 0.62, UCL: 0.93).

Five studies reported measures of home environment risk factors. Katz and Fox (2010) measure residential mobility (having moved home in the previous year), which showed no significant difference between youth gang members and non‐gang youth. Pyrooz and Decker (2013) reported two measures of home environment risk: household strain, and a broken home. Both measures were associated with greater youth gang membership, but not significantly so, and the pooled estimate is also positive but not statistically significant. Olate et al. (2012) reported three measures of home environment risk: difficultly at home, an unstable home, and the young person having their own child at home. Whilst none of these three measures were individually significantly related to youth gang membership, the overall pooled estimate from this study was positive and statistically significant. Abramovay et al. (1999) reported one measure of violence in the family, which was significantly associated with increased odds of youth gang membership. Finally, Ohene et al. (2005) reported one measure of home environment risk, reported separately for males and females: the respondent had run away from home. Running away from home was significantly associated with increased odds of youth gang membership. Overall, the results of these five studies showed that a difficult home life was associated with nearly double the odds of youth gang membership (OR: 1.92, LCL: 1.33, UCL: 2.79), although there is significant heterogeneity between studies (I2: 94.5%, p=<0.001; τ2=0.187).

Three studies provided measures of a protective home environment (Abramovay et al., 1999; Katz and Fox, 2010; Pyrooz and Decker, 2013). Abramovay et al. (1999) showed that young people who lived with both parents were approximately half as likely to be youth gang members as those who did not. Katz and Fox (2010) and Pyrooz and Decker (2013) each reported one measure of protective family environment. Both opportunities for pro‐social involvement within the family (Katz and Fox, 2010) and family attachment (Pyrooz and Decker, 2013) were associated with less youth gang membership, but these estimates were not individually statistically significant. Combining the estimates from these three studies showed that a positive family environment was approximately 25% lower odds of youth gang membership (OR: 0.76, LCL: 0.62, UCL: 0.93), and there was no significant heterogeneity amongst the studies (I2: 0.9%, p=0.364; τ2=0.000).

From these five studies, it appears that the association with a negative family environment is stronger than that of a supportive family, as a negative family environment is associated with a 92 per cent increase in the odds of youth gang membership, whilst a positive family environment is only associated with a 25 per cent decrease in the odds. Additionally, there is much more variability in the effects of the family risk factors (I2: 94.5%, p=<0.001; τ2=) while the effects of the family protective factors are highly homogeneous (I2: 0.9%, p=0.364; τ2=0.000).

Although the results of Moravcová (2012) could not be included in the analyses, their study shows no significant relationship between family structure (single parent status) and gang membership. There are somewhat mixed results for the relationship between family bonds and gang‐membership: significantly weaker family bonds were seen in the Eurogang and Mokken groups compared to non‐gang involved youth, but no significant difference was seen between self‐identified gang members and non‐gang involved youth.

**Figure 13:**
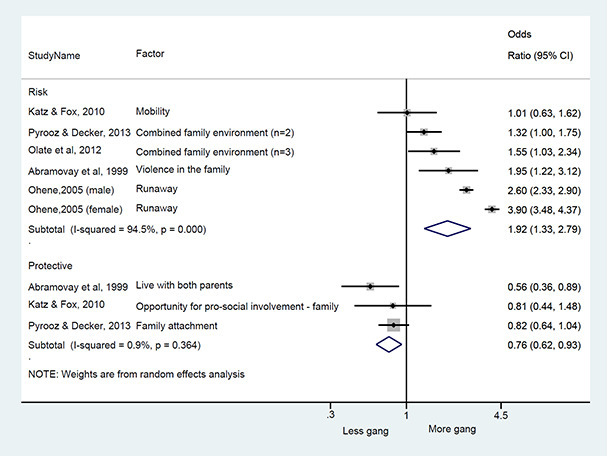
Family environment

##### Family income

Family income was measured concurrently with youth gang membership in one study (Celbis et al., 2012), which measured the frequency of gang membership across three measures of family income; low, medium and high. Family income is therefore treated as a correlate rather than a predictor. Middle income was significantly associated with less youth gang membership than either high family income (OR: 0.23, LCL: 0.12, UCL: 0.47)or low family income (OR: 0.35, LCL: 0.17, UCL: 0.73). There was no significant difference in the occurrence of youth gang membership between low or high family income groups (OR: 0.67, LCL: 0.28, UCL: 1.64).

##### Parental attitudes to antisocial behaviour

Katz and Fox (2010) provided one measure of parental attitudes favourable towards antisocial behaviour: a three item scale gauging youth's perception of their parents’ attitudes towards theft, graffiti and fighting. Higher scores indicated more favourable perceived attitudes, thus this measure was interpreted as a risk factor. There was no significant association between parental attitudes favourable to antisocial behaviour and youth gang membership (OR: 0.95, LCL: 0.59, UCL: 1.51).

##### Parental monitoring

Pyrooz and Decker (2013) provided one measure of parental monitoring: a four‐point scale of the extent to which parents monitored the young person's activities or whereabouts. Higher scores on the four‐point scale indicated higher levels of monitoring, thus this measure was interpreted as a protective factor. Parental monitoring was a significant correlate of youth gang membership (OR: 0.32, LCL: 0.26, UCL: 0.41). An increase of one unit on this scale of parental monitoring was associated with a 68 per cent reduction in the odds of youth gang membership.

##### Parental education

Two studies measured the relationship between parental education and youth gang membership (Celbis et al., 2012; Pyrooz and Decker, 2013). Whilst parental education may intuitively be expected to predate the onset of youth gang membership, this assumption may not hold for parents who go on to increase their educational attainment during their child's adolescence. As a result, this factor was classified as a correlate and was theorized to be a protective factor.

Pyrooz and Decker (2013) measured parental education on an ordinal scale from “grade school” to “graduate or professional school”. Celbis and colleagues (2012) measured mother's education as “incomplete”, “primary” or “secondary”. There was no statistically significant association with gang membership (OR: 1.10, LCL: 0.84, UCL: 1.44), and there was no significant heterogeneity between the studies (I2: 21.7%, p=0.258; τ2=0.011).

**Figure 14:**
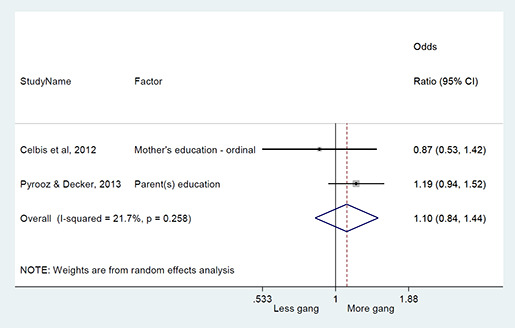
Parental education

##### Exposure to violence in the family

Celbis et al. (2012) measured the association between exposure to five different kinds of violence in the home and youth gang membership: emotional violence, physical violence, property stolen or damaged, threatened or injured with a weapon, and sexual violence. We find a positive association with youth gang membership for all measures; however, there is significant heterogeneity within this set of measures (I2: 62.1%, p=0.032; τ2=0.276).

Neither emotional nor physical violence in the home showed a significant association with youth gang membership, whilst property damage, weapon threats, and sexual violence in the home were each individually significantly associated with youth gang membership. Of the individual measures, the largest association is with exposure to sexual violence (OR: 6.49, LCL: 2.30, UCL: 18.29). Exposure to violence in the home is a significant risk factor overall when combining all measures, associated with a doubling of the odds of youth gang membership (OR: 2.17, LCL: 1.21, UCL: 3.91).

##### School correlates

The included studies contained data on measures across the following categories: level of education, exposure to violence at school, the school environment, individual school performance and attachment. Each of these factors is classified as a correlate as they are either measured concurrently with youth gang membership, or report the respondent's level of education but do not specifically report the link between the age leaving school and the age of onset of youth gang membership. The associations are presented below.

##### Level of education

Four studies reported on the relationship between a young person's current level of education and youth gang membership (Abramovay et al., 1999; Celbis et al., 2012; Olate et al., 2011; Webb et al., 2011). As with parental education, higher levels of youth education were categorized as a protective factor.

Olate et al. (2013) reported high school graduation as a binary variable. Abramovay et al. (1999), Celbis et al. (2012) and Webb et al. (2011) each reported the young person's educational grade, which was then converted to an ordinal effect. Three out of four of the measures were negatively associated with gang membership, but the only individually significant item was high school graduation, which was associated with a reduction in the odds of gang membership. The pooled effect was a non‐significant relationship between higher levels of education and lower odds of youth gang membership, with no significant heterogeneity between studies (OR: 0.73, LCL: 0.46, UCL: 1.18; I2: 57.8%, p=0.068; τ2=0.139).

**Figure 15:**
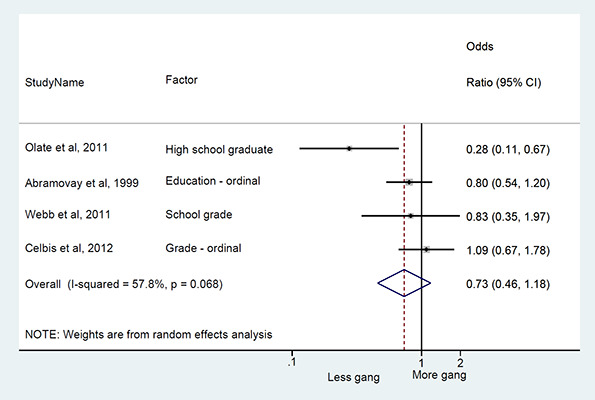
Level of education

##### Exposure to violence at school

As with exposure to violence in the family, these measures by Celbis and colleagues (2012) measured the association between exposure to five different kinds of violence at school and youth gang membership: emotional violence, physical violence, property stolen or damaged, threatened or injured with a weapon, and sexual violence. Each of the individual measures showed a significant positive association with youth gang membership. There is again significant heterogeneity within this set of items (I2: 69.2%, p=0.011; τ2=0.206), with property and emotional violence showing smaller associations than physical, sexual or weapon violence in the school. Overall, exposure to violence at school is a significant risk factor, associated with a trebling of the odds of youth gang membership (OR: 3.29, LCL: 2.04, UCL: 5.32).

##### School environment

Three studies included measures relating to the school environment, including the type of school (public, general, private, vocational or special) (Celbis et al., 2012; Webb et al., 2011) and a measure of opportunities for pro‐social involvement at school (Katz and Fox, 2010). Public and general schools were characterized as a risk factor, whilst special, private and vocational schools were categorized as protective, largely due to their potential for more focused education. School type was dichotomized accordingly for this analysis. The pooled effect shows no significant association between public and general schools on gang membership (OR: 0.93, LCL: 0.59, UCL: 1.47). Overall, there is no measurable heterogeneity amongst between the studies (I2: 0.0%, p=0.832; τ2=0.000).

**Figure 16:**
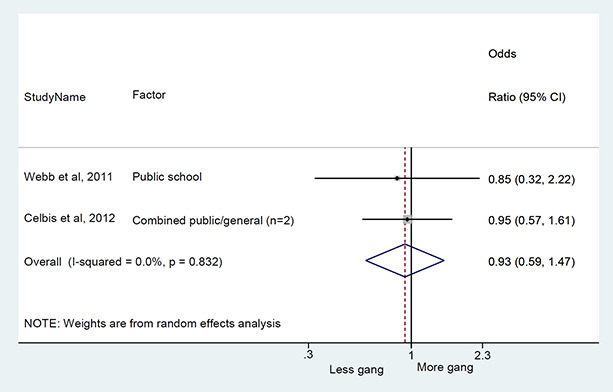
School environment

Katz and Fox (2010) provided a different measure of school environment: opportunity for prosocial involvement at school. Interestingly, and somewhat counterintuitively, this measure shows a significant positive relationship with youth gang membership, whereby young people who reported that their school provided opportunities for prosocial involvement had nearly twice the odds of being in a youth gang (OR: 1.92, LCL: 1.05, UCL: 3.51). This may result from a selection effect, and we hypothesize that proactive schools which note a growing problem with gang membership may be likely to counter with additional opportunities for students.

##### School performance

School performance was measured by three studies (Celbis et al., 2012; Olate et al., 2012; Pyrooz and Decker, 2013). Celbis et al. (2012) reported school performance on a three‐point scale of “poor”, “average”, and “good” overall. Pyrooz and Decker (2013) report school performance as a self‐reported grades in Chinese, Maths and English on a five‐point scale from “poor” to “excellent”. Higher educational performance was categorized as a protective factor. Olate et al. (2012) reported “educational difficulty” as a dichotomous variable, and this was categorized as a risk factor. Educational difficulty was not reverse coded and included in the meta‐analysis, as we hypothesized that a lack of educational difficulty did not necessarily equate to higher school performance.

**Figure 17:**
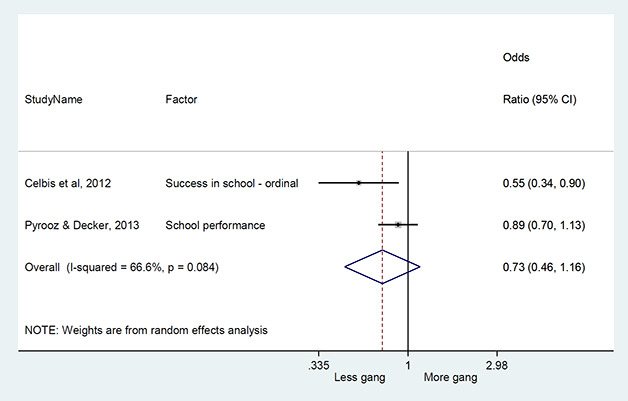
School performance

The pooled estimate of the two measures of success in school was negative but not significant (OR: 0.73, LCL: 0.46, UCL: 1.16) and there was no significant heterogeneity between studies (I2: 66.6%, p= 0.084; τ2=0.078). Conversely, educational difficulty showed a significant positive association with youth gang membership (OR: 2.37, LCL: 1.33, UCL: 4.23). This finding indicates that whilst educational difficulty is a risk factor, success in school is not directly protective.

##### Low school attachment

Five studies reported six independent measures for low school attachment (Abamovay et al., 1999; Katz and Fox, 2010; Ohene et al., 2005; Olate et al, 2011, 2012; Pyrooz and Decker, 2013). Abramovay et al. (1999) reported whether the young person was no longer attending school. Katz and Fox (2010) measured low commitment to school. Ohene et al. (2005) reported whether the young person skipped school, separately for males and females. Olate et al. (2011) reported school dropout and whether the young person was still attending school, and Olate et al. (2012) reported school expulsion. As these two papers analysed the same data, these three estimates were synthesized before they were included in the meta‐analysis. Pyrooz and Decker (2013) reported a measure of school attachment, which was reverse coded for this analysis to represent low school attachment.

Similarly to school performance, the pooled risk factor items showed a positive association with youth gang membership whereby those youth with low attachment to school had just over twice the odds of gang membership as those who did not report low school attachment (OR: 2.05, LCL: 1.67, UCL: 2.53). Unlike school performance, there was significant variability amongst the measures (I2: 80%, p<=0.001; τ2=0.046).

**Figure 18:**
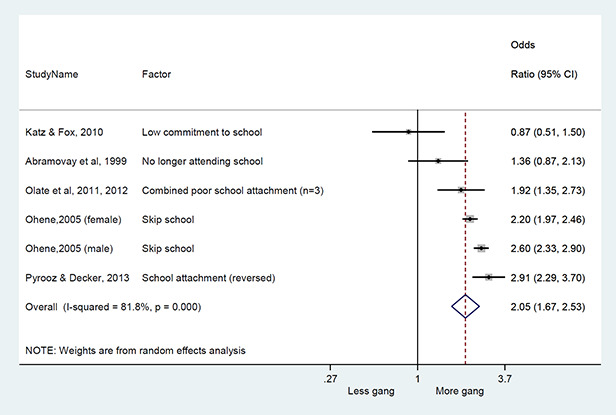
School attachment

##### Community correlates

The included studies provided measures characterising the community youth belong to, including neighbourhood environment, geography, and exposure to crime in the neighbourhood, which are discussed below. Each of these factors were measured concurrently with youth gang membership, and are therefore classified as correlates.

##### Neighbourhood environment

Two studies measured risk factors associated with disordered and disorganized communities, as well as measures associated with pro‐social communities (Olate et al., 2012; Katz and Fox, 2010).

The measures categorized as risk factors were neighbourhood disorder (Olate et al., 2012) and perceived availability of handguns (Katz and Fox, 2010). Of the two risk factors, only perceived handgun availability had a significant relationship with youth gang membership. Young people who reported that they would be able to locate a weapon in their neighbourhood were significantly more likely to be in a youth gang. However, it may be that the gang membership was the cause of perceived gun availability, rather than gun availability being an indicator of a highly disordered community.

The measures categorized as protective factors were neighbourhood social support (Olate et al., 2012) and rewards for pro‐social involvement in the community (Katz and Fox, 2010). Neither measure was individually significant.

**Figure 19:**
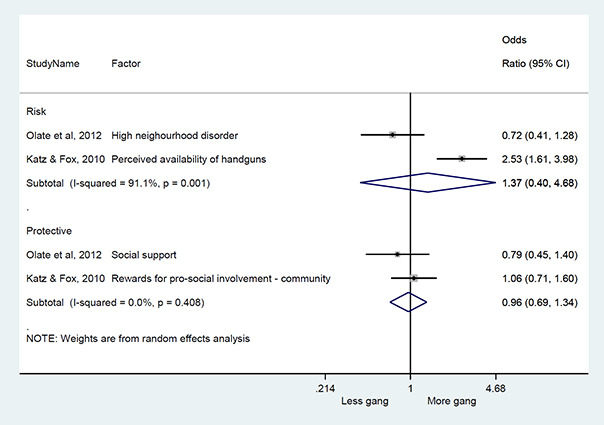
Neighbourhood environment

The overall estimates for both community risk factors and protective factors were not statistically significant (OR: 1.37, LCL: 0.40, UCL: 4.68 and OR: 0.96, LCL: 0.69, UCL: 1.34). For risk factors heterogeneity between studies was high (I2: 91.1%, p=0.001; τ2=0.716), whereas the results from the studies of protective factors were highly homogeneous (I2: 0%, p=4.08; τ2=0.000).

##### Geography

Two studies provided measures of geographic locality (Celbis et al., 2012; Pyrooz and Decker, 2013). These items measured whether the respondent's school was in a suburban (as compared to an urban) area (Celbis et al., 2012), and whether the respondent's residence was in a rural area (Pyrooz and Decker, 2013). These two measures were categorized as protective factors. Whilst these were highly homogeneous measures (I2: 0.0%, p=0.568; τ2=0.000), there was no significant association between the pooled effect of these geographic locations and the odds of youth gang membership (OR: 1.22, LCL: 0.93, UCL: 1.59).

**Figure 20:**
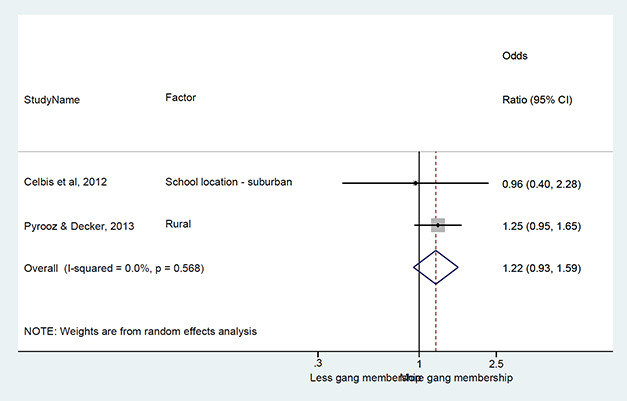
Geography

##### Exposure to violence in the community

Ohene et al. (2012) contributed five measures of exposure to violence in the community: emotional violence, physical violence, property stolen or damaged, threatened or injured with a weapon, and sexual violence. Each measure was individually significantly associated with increased odds of youth gang membership. As with both school and family environments, the pooled estimate of exposure to violence in the community was strongly associated with youth gang membership, with those youth who report exposure to violence in the community having on average over three times the odds of youth gang membership as those who do not(OR: 3.39, LCL: 2.07, UCL: 5.55). Again, these measures are heterogeneous (I2: 67.5%, p=0.015; τ2=0.213), with a smaller association for emotional and property damage, and larger association for sexual violence.

## Discussion

### Summary of main results

#### Overview

Table 8 presents a summary of odds ratios, confidence intervals and the number of independent estimates for each association. This table is sorted in descending order within domains by the amount of evidence that contributes to the overall measure and the size of this estimate. We now present a brief summary of the results, beginning with the areas in which the evidence is strongest.

**Table 8:** Summary of results by domain and association

**Domain**	**Association**	**OR**	**CI**	**N**	**I^2^ %**	**τ^2^**
Individual	Violent delinquency	5.83	5.12 – 6.63	6	48	0.010
	Alcohol and soft drugs (risk)	3.23	2.57 – 4.07	5	0	0
	Gender (male)	2.04	1.35 – 3.08	5	62^*^	0.124
	Age	1.06	0.74 – 1.50	4	55	0.068
	Non‐violent delinquency	4.67	3.80 – 5.76	3	0	0
	Delinquency (general)	3.65	1.89 – 7.04	3	91^*^	0.306
	Sexual behaviour (risk)	3.29	3.00 – 3.62	3	19	0.001
	Employment	1.91	1.07 – 3.63	2	10	0.019
	Psychological (risk)	1.51	1.21 – 1.88	2	0	0
	Psychological (protective)	0.57	0.42 – 0.77	2	0	0
	Hard drugs	4.80	3.06 – 7.52	1	‐	‐
	Victimisation	2.39	1.00 – 5.71	1	‐	‐
	Minority ethnicity (Han Chinese vs other)	2.05	0.76 – 5.55	1	‐	‐
	Alcohol and soft drugs (protective)	0.69	0.42 – 1.13	1	‐	‐
	Sexual behaviour (protective)	0.67	0.28 – 1.60	1	‐	‐
Peer	Delinquent peers	3.96	1.19 – 13.20	3	96^*^	1.073
	Prosocial peers	1.05	0.77 – 1.44	1	‐	‐
Family	Family environment (risk)	1.92	1.33 – 2.79	6	95^*^	0.187
	Family environment (protective)	0.76	0.62 – 0.93	3	1	0
	Parental education	1.10	0.84 – 1.44	2	22	0.011
	Exposure to violence in the home	2.17	1.21 – 3.91	1	‐	‐
	Parental attitudes favourable to antisocial behaviour	0.95	0.59 – 1.51	1	‐	‐
	Family income (low vs high)	0.67	0.28 – 1.64	1	‐	‐
	Family income (middle vs low)	0.35	0.17 – 0.73	1	‐	‐
	Parental monitoring	0.32	0.26 – 0.41	1	‐	‐
	Family income (middle vs high)	0.23	0.12 – 0.47	1	‐	‐
School	Low school attachment	2.05	1.67 – 2.53	6	82^*^	0.046
	Level of education	0.73	0.46 – 1.18	4	58	0.139
	School environment (public/general vs other)	0.93	0.59 – 1.47	2	0	0
	School performance (protective)	0.73	0.46 – 1.16	2	67	0.078
	Exposure to violence at school	3.29	2.04 – 5.32	1	‐	‐
	School performance (educational difficulty)	2.37	1.33 – 4.23	1	‐	‐
	School environment (opportunities for prosocial involvement)	1.92	1.05 – 3.51	1	‐	‐
Community	Neighbourhood environment (risk)	1.37	0.40 – 4.68	2	91^*^	0.716
	Geography	1.22	0.93 – 1.59	2	0	0
	Neighbourhood environment (protective)	0.96	0.69 – 1.34	2	0	0
	Exposure to violence in neighbourhood	3.39	2.07 – 5.55	1	‐	‐

* Significant heterogeneity (p<0.05)

##### Individual associations

This systematic review provided summary estimates for 15 associations between individual factors and youth gang membership. The strongest evidence is for the associations between youth gang membership and delinquency, the use of alcohol and soft drugs, male gender, and sexual behaviours. The results are summarized below, with the number of independent measures reported in parentheses to allow consideration of the amount of evidence contributing to the summary.

Delinquency was assessed across three categories. Youth who reported engaging in violent delinquency had nearly six times the odds of being a youth gang members as those that did not report violent delinquency (n=6) and non‐violent delinquency was associated with over four times the odds of youth gang membership (n=3). Indeed, violent delinquency had both the strongest association with youth gang membership and was based on the largest number of studies among the results. General delinquency was associated with more than three and a half times the odds of youth gang membership (n=3); however, this finding was based on studies with a very high degree of variability in outcomes.

The use of alcohol and soft drugs (cigarettes and marijuana) was associated on average with over three times the odds of reporting youth gang membership (n=5). There was no significant association between later onset of alcohol and soft drug use and youth gang membership (n=1). Whilst hard drugs was associated with almost five times the odds of youth gang membership (n=1), this result is drawn from only one study so is a much less robust finding than that seen for alcohol and soft drugs.

There is relatively strong evidence for the association between male gender and youth gang membership, with males having twice the odds of youth gang membership than females (n=5); however, this finding relies on studies that have a high degree of variability in outcomes. There was no significant relationship seen between age and youth gang membership (n=4).

A very small number of studies reported on the association between sexual behaviours and youth gang membership. Sexual activity was associated with over three times the odds of youth gang membership (n=3); however there was no significant relationship with protective sexual behaviours such as delayed age of first intercourse and condom use (n=1).

There is weak evidence for the counterintuitive finding that employment is associated with nearly double the odds of youth gang membership (n=2), although employment need not be legitimate and this association may be related to increased opportunity for employment following school disengagement.

There is also weak evidence that psychological factors are associated with youth gang membership. Psychological risk factors (impulsivity and low self‐control) are associated with 51 per cent greater odds of youth gang membership (n=2) whilst psychological protective factors are associated with 43 per cent lower odds (n=2).

Finally, youth who report victimisation had more than twice the odds of also reporting youth gang membership (n=1) and there was no significant association seen between youth gang membership and ethnic minority group membership (n=1). Given that both of these analyses relied on only one study each, this evidence is extremely limited.

Apart from gender and ethnicity, none of these relationships was time invariant or measured retrospectively. Therefore, at best, these studies report differences in the activities and behaviours between youth gang members and non‐gang youth. Whilst these results do confirm higher risk taking behaviours and greater victimisation among gang involved youth, they do not provide insight into the individual predictors of youth gang membership.

##### Peer associations

There is a small amount of evidence that socialising with delinquent peers is associated with nearly four times the odds of youth gang membership (n=3); however, this finding was based on studies with a high degree of variability in outcomes. There is no significant relationship between socialising with pro‐social peers and youth gang membership (n=1). Again, it must be noted that these findings are based on very limited evidence.

##### Family associations

Negative family environments are associated with nearly twice the odds of youth gang membership (n=6). Whilst this association is based on one of the largest sets of independent estimates in this review, it is important to note that it is also based on studies with a high degree of variability in outcomes. In contrast, a positive family environment is associated with approximately 25 per cent lower odds of gang membership (n=3), although there is less evidence contributing to this finding.

There was no significant relationship seen between parental education and youth gang membership (n=2), nor between parental attitudes to antisocial behaviour and youth gang membership (n=1); however parental monitoring was associated with reduced odds of youth gang membership (n=1). Finally, and again perhaps counterintuitively, youth from middle income families had greater odds of reporting youth gang membership than those from either high or low income families, which showed no significant differences to one another (n=1). Again, it is importance to recognize the limited evidence supporting these findings.

##### School associations

This review provided evidence for seven associations between school factors and youth gang membership. The strongest evidence is for the associations between youth gang membership and low school attachment. Youth who report lower attachment have twice the odds of also reporting gang membership (n=6); however, this result is based on findings with a high degree of variation in outcomes. There was no significant relationship seen between a young person's current level of education and youth gang membership (n=4).

There was no significant association between youth gang membership and public school environment (n=2), nor with a young person's performance at school (n=2). Limited evidence suggests that exposure to violence at school is associated with triple the odds of youth gang membership (n=1), and that both educational difficulty (n=1) and opportunities for prosocial involvement at school (n=1) are associated with approximately twice the odds of youth gang membership. These results come from one study only and are not the result of a meta‐analytic synthesis of multiple studies.

##### Community associations

Finally, there is very limited evidence in relation to the association between community factors and youth gang membership in low‐ and middle‐income countries. Neither neighbourhood risk factors (n=2), neighbourhood protective factors (n=2), nor geographic location (n=2) are significantly associated with youth gang membership. Youth exposed to violence in the neighbourhood have more than three times the odds of youth gang membership than non‐exposed youth (n=1); however, this finding was based on only one study.

#### Experience across domains

As Table 8 shows, there were 37 total associations calculated from the included studies. The majority of associations were in the individual (15 of 37), family (9 of 37) and school (7 of 37) domains. Community and peer domains contributed only four and two associations respectively. Consequently, while we initially have a very small number of robust studies from which to draw conclusions, what small number of associations there are tend to concentrate in the individual, family and school domains, and there are considerably fewer in the peer and community domains.

This small amount of evidence in each domain is further weakened when we consider the small number of independent estimates that contribute to each analysis of associations. After accounting for dependencies from several measures that come from the same study, there were 85 independent estimates contributing to the final 37 analyses. We find the strongest evidence for the three analyses that synthesize six independent estimates: violent delinquency, school attachment, and negative family environment. Two analyses synthesize five independent measures: alcohol and drug use, and male gender. Two analyses synthesize four independent studies:age, and level of education. At the other extreme, for 16 different outcome variables we only identified one independent measure, nine are supported by only two independent measures, and five are supported by three independent measures. This dearth of evidence is most striking in the community and peer domains, where none of the four analyses of community factors contain more than two independent estimates, and the two analyses of peer factors contain no more than three independent estimates. This very small amount of evidence contributing to the analyses, limits any generalisations from these associations.

### Overall completeness and applicability of evidence

This systematic search was conducted across a very broad range of databases, websites and grey literature sources in multiple languages, and we conducted reference harvesting and contact with key researchers to ensure completeness. The final set of eight eligible studies allowed an analysis of the associations between risk and protective factors across five key domains. The included studies were conducted in Turkey, Trinidad and Tobago, the Caribbean, El Salvador, China, and Brazil. The majority of studies examined individual risk and protective factors, although there was representation from the other key domains. We are confident that this set of studies represents a complete body of work that meets our methodological and substantive criteria.

It is important to recognize that, even though we are confident that we have found a complete reflection of the available evidence, the available body of evidence is very small, and by no means fully addresses the question of which factors predict youth gang membership in low‐ and middle‐income countries. There are significant gaps in the literature, and many of the factors from the developmental model of gang membership are not represented in these studies. Very few studies analysed the same correlates in different geographic and socio‐economic contexts, there is no representation of studies from Africa, and the majority of studies focus on individual factors. One of the major limitations is the lack of longitudinal studies, which could help identify predictive factors, and not just simply associations.

### Quality of the evidence

None of the eight included studies was of poor quality. Indeed, in general, the authors were clear and expansive in their descriptions of their studies, with most facets being described in a manner that would allow some degree of accurate replication. The sample sizes were very large, and pooled to include the experiences of over 23,500 young people in low‐ and middle‐income countries.

The included studies were generally conducted with methodological rigour, but we were largely only able to calculate effect sizes from bivariate associations. As a result, for the majority of estimates there was no statistical matching of the gang sample to the non‐gang sample, so the effect sizes are largely calculated on an unmatched sample. This is not necessarily a reflection on the quality of the studies, as it must be noted that several of the studies were in fact focused on determining the correlates of other youth behaviours, and used appropriately advanced statistical models to do so. However, in order to extract useful data for this review, only the descriptive statistics could be employed, as the more advanced models did not report the necessary data to calculate effect sizes.

The core issue with the review is that none of the studies had the appropriate temporal ordering to enable causal inference. Each of the included studies used a cross‐sectional design, and none applied a retrospective approach to any of the questions in their surveys. Therefore, at best, the review can show associations, but not causation, except in the case of time‐invariant correlates.

### Limitations and potential biases in the review process

As discussed, the main limitations are the small number of studies and the lack of a predictive analytic framework. However, the studies are largely comparable in many of the definitions, with a standardized approach in many instances. Standardisation is not necessarily the equivalent of validation, however, and whilst many items had been drawn from standardized instruments, these were not in the majority.

One definition that is not strictly comparable is the classification of youth into gang members or gang‐involved youth. The definitions of gang members and gang‐involved youth, whilst broadly conforming to the literature, varied in their exact application across the included studies. The term ‘gang’ was generally undefined in the surveys, which may lead to some lack of comparability across studies. Due to a small number of studies, analyses in this review were pooled for gang members and gang‐involved youth, rather than being conducted separately. Whilst we acknowledge that this may introduce bias, the variation in definitions had already prevented a very precise delineation between current and former gang members.

There are some further limitations to consider, particularly in terms of generalizability from this review. The use of school samples in the majority of studies is a considerable limitation to the scope of this review. In the majority of the studies, only school‐engaged adolescents were sampled, and it might reasonably be anticipated that school‐engaged youth gang members may not be representative of youth gang members more broadly. Likewise, young adults in their 20s are only included in two of the study samples, as the school samples limit the upper age of participants to approximately 18 years old.

It is also important to note that by only having one author screen each document, rather than performing double screening, it is possible that some bias may have been introduced to the review.

Finally, whilst the studies were drawn from the Middle East, Latin America, the Caribbean and Asia, it was unfortunate that there was no representation of African nations. Of course, given the huge diversity of culture and context across low‐ and middle‐income countries, such a small number of studies cannot be considered representative; rather, we hope that this review will highlight the lack of evidence and prove a useful starting point for future research.

### Agreements and disagreements with other studies or reviews

There are no systematic reviews on the factors associated with youth gang membership in low‐ and middle‐income countries to date. However, for the purposes of illustration, table 8 contains the summary of factors sourced from the Small Arms Survey (2010) which was presented in the Background section of this review. We have highlighted the similarities and contradictions with this body of work – bold type indicates some broadly defined areas where the present review found quantitative supporting evidence, bold italic indicates disagreement in findings or results that do not demonstrate a relationship. It is important to recognize that the current review was only able to examine a small number of these correlates, due to the limited number of included studies. The factors that are not marked in bold type illustrate the limits of the findings.

**Table 9:** Factors associated with gang membership, cross‐referenced to key findings of this review

**Domain**	**Risk Factors**	**Protective Factors**
Community	***Social disorganization, including poverty and residential mobility*** Organized lower‐class communities Underclass communities Presence of gangs in the neighbourhood Availability of drugs in the neighbourhood **Availability of firearms** Barriers to and lack of social and economic opportunities Lack of social capital Cultural norms supporting gang behaviour **Feeling unsafe in neighbourhood; high crime** Conflict with social control institutions	Short or no history of gang presence Strict formal and informal control of firearms Limited neighbourhood congregation sites of unsupervised youth Absence of drug markets
Family	**Family disorganization, including broken homes and parental drug or alcohol abuse** **Troubled families, including incest, family violence, and drug addiction** Family members in a gang Lack of adult male role models Lack of parental role models ***Low socio‐economic status*** Extreme economic deprivation, family management problems, parents with violent attitudes, sibling anti‐social behaviour	**Family involvement** Consistent parental discipline Open family communication
School	**Academic failure** Low educational aspirations, especially among females Negative labelling by teachers Trouble at school Few teacher role models **Educational frustration** **Low commitment to school, low school attachment, high levels of anti‐social behaviour in school, low achievement test scores, identification as being learning‐disabled**	Psychosocial support for teachers Parental involvement in schools
Peer group	High commitment to delinquent peers ***Low commitment to positive peers*** Street socialization Gang members in class **Friends who use drugs or who are gang members** Friends who are drug distributors **Interaction with delinquent peers**	Mixed peer network of gang and non‐gang members Intimate partner attachment to non‐gang affiliate
Individual	**Prior delinquency** Deviant attitudes Street smartness; toughness Defiant and individualist character Fatalistic view of the world **Aggression** **Proclivity for excitement and trouble** **Locura (acting in a daring, courageous, and especially crazy fashion in the face of adversity)** Higher level of normlessness in the context of family, peer group, and school Social disabilities Illegal gun ownership **Early or precocious sexual activity, especially among females** **Alcohol and drug use** Drug trafficking Desire for group rewards such as status, identity, self‐esteem, companionship, and protection Problem behaviours, hyperactivity, externalizing behaviours, drinking, and lack of refusal skills **Victimization**	High level of personal resources **Sense of coherence** Positive, culturally relevant identity

## Authors’ conclusions

### Implications for practice and policy

In designing preventive interventions it is essential to understand the causal framework of the behaviour that is being exhibited. Whilst this review does not allow us to go this far, it provides some evidence of the importance of key correlates of youth gang membership, drawn from a small number robust empirical studies.

As with all systematic reviews that draw on few studies, we acknowledge that this is a very limited body of work from which to draw conclusions. The findings, however, do support previous narrative syntheses of the literature. Many of the findings are therefore intuitive and unsurprising; for example, from the literature on youth gangs it is to be expected that young gang members will have higher rates of substance use and delinquency, and will be much more likely to associate with delinquent peers. The value of drawing this evidence from methodologically robust studies is that these studies demonstrate that these are qualities that separate youth gang members from their non‐gang peers.

While many of the individual and peer associations identified in this review (such as delinquency, drug use, and sexual risk factors) may be as a result of gang membership rather than its cause, our results suggest certain family, school, and community level factors associated with gang membership that could be addressed through targeted preventive interventions. In particular, we would highlight the associations between youth gang membership and family environment, parental monitoring, school attachment, educational difficulties, and exposure to violence in the home, at school, or in the community.

Policy‐makers and practitioners can use this evidence, drawn from a small number of methodologically robust studies in low‐ and middle‐income countries, to assist with frameworks for prevention. We suggest that the identified themes are starting off points, where the relative strength of the associations can be used to inform primary, secondary or tertiary gang prevention programs. The results from our systematic review on the impact of preventive youth gang interventions demonstrate that there is currently no robust evidence to indicate which interventions work in low‐ and middle‐income countries (Higginson *et al*., 2015). The current study may suggest factors that may drive gang membership, and suggest areas where interventions may prove promising.

### Implications for research

This systematic review highlights the small set of studies that can be used to make robust statements about the characteristics and experiences that differentiate youth gang members from non‐gang members. It is our hope that this research continues to expand. Whilst there are only eight included studies, these studies represent the end result of examining over 50,000 documents in order to learn from the experiences of over 23,500 young people included in the pooled sample of this systematic review.

In terms of substantive focus, it appears that the influence of peers and communities in particular is underrepresented in our data. The greatest proportion of effect sizes in this review were for individual factors, yet there is strong theoretical work that hypothesizes an interconnection of individual, peers, family, school, and community factors. We would encourage research that expands to these other, potentially equally important domains, and ideally also tackles the methodological issues of clustered data that will inevitably be encountered when departing from the study of the individual.

Methodologically, we would particularly encourage researchers to develop longitudinal studies of youth delinquency in low‐ and middle‐income countries so that some causal evidence on the predictors of youth gang membership can be forthcoming. Several of the studies included in this review utilize large survey instruments that would be greatly enhanced by repeated application over time, both for the current cohort and for future cohorts, in order to develop a longitudinal study of a very large sample of young people.

Failing a longitudinal approach, some evidence for predictors could come from studies that framed survey questions retrospectively, so that there was no ambiguity regarding the temporal stability of a correlate, or whether a correlate predates the onset of youth gang membership. Ideally, such future studies would also seek to use standardized measures so as to assist with replication and comparability of results. It is this kind of investment in research effort that will allow a true evaluation of the predictors of youth gang membership.

## Information about this review

### Review authors

**Lead review author:**

**Table:** 

**Name:**	**Angela Higginson**
Title:	Dr
Affiliation:	School of Justice, Queensland University of Technology
Address:	GPO Box 2434
City, State, Province or County:	Brisbane, Queensland
Postal Code:	4001
Country:	Australia
Email:	angela.higginson@qut.edu.au
**Co‐authors:**	
**Name:**	**Joseph Murray**
Title:	Dr
Affiliation:	Department of Psychiatry, University of Cambridge
Address:	Douglas House, 18b Trumpington Road
City, State, Province or County:	Cambridge
Postal Code:	CB2 8AH
Country:	United Kingdom
Phone:	+44 (0)1223 335388
Email:	jm335@cam.ac.uk
**Name:**	**Kathryn Benier**
Title:	Dr
Affiliation:	School of Social Science, The University of Queensland
Address:	Campbell Road, St Lucia
City, State, Province or County:	Brisbane, Queensland
Postal Code:	4072
Country:	Australia
Email:	k.benier@uq.edu.au
**Name:**	**Yulia Shenderovich**
Title:	Ms
Affiliation:	Department of Psychiatry, University of Cambridge
Address:	Douglas House, 18b Trumpington Road
City, State, Province or County:	Cambridge
Postal Code:	CB2 8AH
Country:	United Kingdom
Phone:	+44 (0)1223 746055
Email:	ys416@medschl.cam.ac.uk
**Name:**	**Laura Bedford**
Title:	Ms
Affiliation:	The University of Queensland Institute for Social Science Research and Australian Research Council (ARC) Centre of Excellence in Policing and Security (CEPS)
Address:	Campbell Road, St Lucia
City, State, Province or County:	Brisbane, Queensland
Postal Code:	4072
Country:	Australia
Phone:	+617 3366 7475
Email:	l.bedford@uq.edu.au
**Name:**	**Lorraine Mazerolle**
Title:	Professor
Affiliation:	School of Social Science, The University of Queensland
Address:	Campbell Road, St Lucia
City, State, Province or County:	Brisbane, Queensland
Postal Code:	4072
Country:	Australia
Phone:	+617 3346 7877
Email:	l.mazerolle@uq.edu.au

### Roles and responsibilities

- Content: Angela Higginson, Joseph Murray, Lorraine Mazerolle, Kathryn Benier, Laura Bedford
- Systematic review methods: Angela Higginson, Joseph Murray, Yulia Shenderovich
- Statistical analysis: Angela Higginson
- Information retrieval: Yulia Shenderovich, Kathryn Benier, Laura Bedford

### Sources of support

**Internal funding:**

Support for this study was provided by the Institute for Social Sciences Research, the University of Queensland, and the ARC Centre of Excellence in Policing and Security.

**External funding:**

This review is externally funded by USAID through 3ie (International Initiative for Impact Evaluation, Inc.)(SR5/1117). The views expressed in this article are not necessarily those of USAID or 3ie or its members.

Funding for the broader database searching (Murray et al., 2013) was provided by the Wellcome Trust [089963/Z/09/Z]

### Declarations of interest

None of the authors have any known conflict of interest.

### Plans for updating the review

The authors plan to update the review every five years.

### Author declaration

**Authors’ responsibilities**

By completing this form, you accept responsibility for maintaining the review in light of new evidence, comments and criticisms, and other developments, and updating the review at least once every five years, or, if requested, transferring responsibility for maintaining the review to others as agreed with the Coordinating Group. If an update is not submitted according to agreed plans, or if we are unable to contact you for an extended period, the relevant Coordinating Group has the right to propose the update to alternative authors.

**Publication in the Campbell Library**

The Campbell Collaboration places no restrictions on publication of the findings of a Campbell systematic review in a more abbreviated form as a journal article either before or after the publication of the monograph version in *Campbell Systematic Reviews*. Some journals, however, have restrictions that preclude publication of findings that have been, or will be, reported elsewhere, and authors considering publication in such a journal should be aware of possible conflict with publication of the monograph version in *Campbell Systematic Reviews*. Publication in a journal after publication or in press status in *Campbell Systematic Reviews* should acknowledge the Campbell version and include a citation to it. Note that systematic reviews published in *Campbell Systematic Reviews* and co‐registered with the Cochrane Collaboration may have additional requirements or restrictions for co‐publication. Review authors accept responsibility for meeting any co‐publication requirements.

**I understand the commitment required to update a Campbell review, and agree to publish in the Campbell Library. Signed on behalf of the authors:**

**Form completed by: Angela Higginson**

**Date: 19 June 2015**

## Footnotes

1 The Advisory Group was made up of scholars and policy‐makers in the field. A full list of members can be found in the Title Registration document for this review (Higginson et al., 2013).

## Appendix A: Search strategy structure

**A** AND **B** AND **D**

or

**C** AND **D**

**Table:**

Concept	Search terms
**A**	aggression
	antisocial behaviour
	behavior disorder
	behavior problem
	bullying
	conduct disorder
	conduct problem
	crime
	criminal behavior
	disruptive behaviour disorder
	externalising
	externalizing
	gang
	homicide
	oppositional defiant disorder
	school violence
	social behavior disorders
	violence
	violent crime
	workplace violence
**B**	child
	youth
	infant
	baby
	toddler
	adolescent
	teenager
**C**	juvenile delinquency
	child behavior disorders
	school violence
**D**	Africa or Central Africa or Latin America or Caribbean or West Indies or Eastern Europe or Soviet or South America or Arab or Middle East or Latin America or Central America Afghanistan or Albania or Algeria or Angola or Antigua or Barbuda or Argentina or Armenia or Armenian or Aruba or Azerbaijan or Bahrain or Bangladesh or Barbados or Benin or Byelarus or Byelorussian or Belarus or Belorussian or Belorussia or Belize or Bhutan or Bolivia or Bosnia or Herzegovina or Hercegovina or Botswana or Brasil or Brazil or Bulgaria or Burkina Faso or Burkina Fasso or Upper Volta or Burundi or Urundi or Cambodia or Khmer Republic or Kampuchea or Cameroon or Cameroons or Cameron or Camerons or Cape Verde or Central African Republic or Chad or Chile or China or Colombia or Comoros or Comoro Islands or Comores or Mayotte or Congo or Zaire or Costa Rica or Cote d'Ivoire or Ivory Coast or Croatia or Cuba or Cyprus or Czechoslovakia or Czech Republic or Slovakia or Slovak Republic or Djibouti or French Somaliland or Dominica or Dominican Republic or East Timor or East Timur or Timor Leste or Ecuador or Egypt or United Arab Republic or El Salvador or Eritrea or Estonia or Ethiopia or Fiji or Gabon or Gabonese Republic or Gambia or Gaza or Georgia Republic or Georgian Republic or Ghana or Gold Coast or Greece or Grenada or Guatemala or Guinea or Guam or Guiana or Guyana or Haiti or Honduras or Hungary or India or Maldives or Indonesia or Iran or Iraq or Isle of Man or Jamaica or Jordan or Kazakhstan or Kazakh or Kenya or Kiribati or Korea or Kosovo or Kyrgyzstan or Kirghizia or Kyrgyz Republic or Kirghiz or Kirgizstan or Lao PDR or Laos or Latvia or Lebanon or Lesotho or Basutoland or Liberia or Libya or Lithuania or Macedonia or Madagascar or Malagasy Republic or Malaysia or Malaya or Malay or Sabah or Sarawak or Malawi or Nyasaland or Mali or Malta or Marshall Islands or Mauritania or Mauritius or Agalega Islands or Mexico or Micronesia or Middle East or Moldova or Moldovia or Moldovian or Mongolia or Montenegro or Morocco or Ifni or Mozambique or Myanmar or Myanma or Burma or Namibia or Nepal or Netherlands Antilles or New Caledonia or Nicaragua or Niger or Nigeria or Northern Mariana Islands or Oman or Muscat or Pakistan or Palau or Palestine or Panama or Paraguay or Peru or Philippines or Philipines or Phillipines or Phillippines or Poland or Portugal or Puerto Rico or Romania or Rumania or Roumania or Russia or Russian or Rwanda or Ruanda or Saint Kitts or St Kitts or Nevis or Saint Lucia or St Lucia or Saint Vincent or St Vincent or Grenadines or Samoa or Samoan Islands or Navigator Island or Navigator Islands or Sao Tome or Saudi Arabia or Senegal or Serbia or Montenegro or Seychelles or Sierra Leone or Slovenia or Sri Lanka or Ceylon or Solomon Islands or Somalia or South Africa or Sudan or Suriname or Surinam or Swaziland or Syria or Tajikistan or Tadzhikistan or Tadjikistan or Tadzhik or Tanzania or Thailand or Togo or Togolese Republic or Tonga or Trinidad or Tobago or Tunisia or Turkey or Turkmenistan or Turkmen or Uganda or Ukraine or Uruguay or USSR or Soviet Union or Union of Soviet Socialist Republics or Uzbekistan or Uzbek or Vanuatu or New Hebrides or Venezuela or Vietnam or Viet Nam or West Bank or Yemen or Yugoslavia or Zambia or Zimbabwe or Rhodesia LMICs developing/less developed/under developed/underserved/deprived/poor countries transitional countries
**PsycINFO (Ovid)** 1967 to 2013	developing countries/ (Africa or "Latin America" or Caribbean or "West Indies" or "Eastern Europe" or Soviet or "South America" or "Middle East" or "Latin America" or "Central America").hw,ti,ab. (Afghanistan or Albania or Algeria or Angola or Antigua or Barbuda or Argentina or Armenia or Armenian or Aruba or Azerbaijan or Bahrain or Bangladesh or Barbados or Benin or Byelarus or Byelorussian or Belarus or Belorussian or Belorussia or Belize or Bhutan or Bolivia or Bosnia or Herzegovina or Hercegovina or Botswana or Brasil or Brazil or Bulgaria or Burkina Faso or Burkina Fasso or Upper Volta or Burundi or Urundi or Cambodia or Khmer Republic or Kampuchea or Cameroon or Cameroons or Cameron or Camerons or Cape Verde or Central African Republic or Chad or Chile or China or Colombia or Comoros or Comoro Islands or Comores or Mayotte or Congo or Zaire or Costa Rica or Cote d'Ivoire or Ivory Coast or Croatia or Cuba or Cyprus or Czechoslovakia or Czech Republic or Slovakia or Slovak Republic or Djibouti or French Somaliland or Dominica or Dominican Republic or East Timor or East Timur or Timor Leste or Ecuador or Egypt or United Arab Republic or El Salvador or Eritrea or Estonia or Ethiopia or Fiji or Gabon or Gabonese Republic or Gambia or Gaza or Georgia Republic or Georgian Republic or Ghana or Gold Coast or Greece or Grenada or Guatemala or Guinea or Guam or Guiana or Guyana or Haiti or Honduras or Hungary or India or Maldives or Indonesia or Iran or Iraq or Isle of Man or Jamaica or Jordan or Kazakhstan or Kazakh or Kenya or Kiribati or Korea or Kosovo or Kyrgyzstan or Kirghizia or Kyrgyz Republic or Kirghiz or Kirgizstan or Lao PDR or Laos or Latvia or Lebanon or Lesotho or Basutoland or Liberia or Libya or Lithuania or Macedonia or Madagascar or Malagasy Republic or Malaysia or Malaya or Malay or Sabah or Sarawak or Malawi or Nyasaland or Mali or Malta or Marshall Islands or Mauritania or Mauritius or Agalega Islands or Mexico or Micronesia or Middle East or Moldova or Moldovia or Moldovian or Mongolia or Montenegro or Morocco or Ifni or Mozambique or Myanmar or Myanma or Burma or Namibia or Nepal or Netherlands Antilles or New Caledonia or Nicaragua or Niger or Nigeria or Northern Mariana Islands or Oman or Muscat or Pakistan or Palau or Palestine or Panama or Paraguay or Peru or Philippines or Philipines or Phillipines or Phillippines or Poland or Portugal or Puerto Rico or Romania or Rumania or Roumania or Russia or Russian or Rwanda or Ruanda or Saint Kitts or St Kitts or Nevis or Saint Lucia or St Lucia or Saint Vincent or St Vincent or Grenadines or Samoa or Samoan Islands or Navigator Island or Navigator Islands or Sao Tome or Saudi Arabia or Senegal or Serbia or Montenegro or Seychelles or Sierra Leone or Slovenia or Sri Lanka or Ceylon or Solomon Islands or Somalia or South Africa or Sudan or Suriname or Surinam or Swaziland or Syria or Tajikistan or Tadzhikistan or Tadjikistan or Tadzhik or Tanzania or Thailand or Togo or Togolese Republic or Tonga or Trinidad or Tobago or Tunisia or Turkey or Turkmenistan or Turkmen or Uganda or Ukraine or Uruguay or USSR or Soviet Union or Union of Soviet Socialist Republics or Uzbekistan or Uzbek or Vanuatu or New Hebrides or Venezuela or Vietnam or Viet Nam or West Bank or Yemen or Yugoslavia or Zambia or Zimbabwe or Rhodesia).hw,ti,ab,cp. ((developing or less* developed or under developed or underdeveloped or middle income or low* income or underserved or under served or deprived or poor* or foreign) adj (countr* or nation? or population? or world or region*)).hw,ti,ab. ((developing or less* developed or under de veloped or underdeveloped or middle income or low* income) adj (economy or economies)).hw,ti,ab. (lmic or lmics or third world or lami countr*).hw,ti,ab. transitional countr*.hw,ti,ab.
	OR/1‐8
	antisocial behavior/ OR
	conduct disorder/ OR
	exp behavior problems/ OR
	behavior disorders/ OR
	impulse control disorders/ OR
	adjustment disorders/ OR
	violence/ OR
	exp violent crime/ OR
	workplace violence/ OR
	crime/ OR
	criminal behavior/ OR
	crime.mp. OR
	crimes.mp. OR
	criminal*.mp. OR
	exp homicide/ OR
	homicid*.mp. OR
	exp perpetrators/ OR
	attack behavior/ OR
	acting out/ OR
	exp gangs/ OR
	gang.mp. OR
	gangs.mp.
	exp bullying/ OR
	bully*.mp. OR
	aggress*.mp. OR
	aggressive behavior/ OR
	(conduct adj1 problem*).mp. OR
	(behavio?r adj1 problem*).mp. OR
	(conduct adj1 disorder*).mp. OR
	(behavio?r adj1 disorder*).mp. OR
	(antisocial adj1 behavio?r*).mp. OR
	(anti‐social adj1 behavio?r*).mp. OR
	(oppositional adj1 defiant adj1 disorder*).af. OR
	(disruptive adj1 behavio?r adj1 disorder*).af.
	(externalizing adj1 behavio?r adj1 problem*).mp.
	externalizing.mp.
	externalising.mp.
	externalized.mp.
	externalised.mp.
	externaliz*.mp.
	externalis*.mp.
	(childhood adj1 externalizing adj1 behavio?r).mp.
	(externalizing adj1 behavio?r).mp.
	(externalising adj1 behavio?r).mp.
	11 or 12 or 13 or 14 or 15 or 16 or 17 or 18 or 19 or 20 or 21 or 22
	10 and 23
	exp Childhood Development/
	Adolescent development/
	Child Welfare/
	Child Care/
	baby.ti,ab.
	babies.ti,ab.
	toddler.ti,ab.
	toddlers.ti,ab.
	adolescen*.ti,ab.
	adolescent.ti,ab.
	adolescents.ti,ab.
	adolescence.ti,ab.
	child*.ti,ab.
	child.ti,ab.
	children*.ti,ab.
	childhood*.ti,ab.
	childhood.ti,ab.
	youth*.ti,ab.
	youth.ti,ab.
	youths.ti,ab.
	student*.ti,ab.
	Students.ti,ab.
	Student.ti,ab.
	teen*.ti,ab.
	teenager.ti,ab.
	teenagers.ti,ab.
	boy.ti,ab.
	boys.ti,ab.
	girl.ti,ab.
	girls.ti,ab.
	pupil.ti,ab.
	pupils.ti,ab.
	pupil*.ti,ab.
	youngster*.ti,ab.
	youngster.ti,ab.
	youngsters.ti,ab.
	juvenile*.ti,ab.
	juvenile.ti,ab.
	juveniles.ti,ab.
	Infant*.ti,ab.
	infant.ti,ab.
	infants.ti,ab.
	young adj1 adult*.ti,ab.
	30 or 31 or 32 or 33 or 34 or 35 or 36 or 37 or 38 or 39 or 40 or 41
	28 and 42
	Or/ 47‐
	AND
	exp juvenile delinquency/
	(juvenile adj1 delinquen*).mp.
	school violence/
	or/
**Ovid MEDLINE(R) In‐Process and Other Non‐Indexed Citations and Ovid MEDLINE(R) 1946 to Present** Ovid MEDLINE(R) In‐Process and Other Non‐Indexed Citations and Ovid MEDLINE(R) 1946 to Presen • Ovid MEDLINE(R) In‐Process and Other Non‐Indexed Citations and Ovid MEDLINE(R) 1946 to Prese	Developing Countries.sh. (Africa or Central Africa or Latin America or Caribbean or West Indies or Eastern Europe or Soviet or South America or Arab or Middle East or Latin America or Central America).hw,kf,ti,ab,cp. (Afghanistan or Albania or Algeria or Angola or Antigua or Barbuda or Argentina or Armenia or Armenian or Aruba or Azerbaijan or Bahrain or Bangladesh or Barbados or Benin or Byelarus or Byelorussian or Belarus or Belorussian or Belorussia or Belize or Bhutan or Bolivia or Bosnia or Herzegovina or Hercegovina or Botswana or Brasil or Brazil or Bulgaria or Burkina Faso or Burkina Fasso or Upper Volta or Burundi or Urundi or Cambodia or Khmer Republic or Kampuchea or Cameroon or Cameroons or Cameron or Camerons or Cape Verde or Central African Republic or Chad or Chile or China or Colombia or Comoros or Comoro Islands or Comores or Mayotte or Congo or Zaire or Costa Rica or Cote d'Ivoire or Ivory Coast or Croatia or Cuba or Cyprus or Czechoslovakia or Czech Republic or Slovakia or Slovak Republic or Djibouti or French Somaliland or Dominica or Dominican Republic or East Timor or East Timur or Timor Leste or Ecuador or Egypt or United Arab Republic or El Salvador or Eritrea or Estonia or Ethiopia or Fiji or Gabon or Gabonese Republic or Gambia or Gaza or Georgia Republic or Georgian Republic or Ghana or Gold Coast or Greece or Grenada or Guatemala or Guinea or Guam or Guiana or Guyana or Haiti or Honduras or Hungary or India or Maldives or Indonesia or Iran or Iraq or Isle of Man or Jamaica or Jordan or Kazakhstan or Kazakh or Kenya or Kiribati or Korea or Kosovo or Kyrgyzstan or Kirghizia or Kyrgyz Republic or Kirghiz or Kirgizstan or Lao PDR or Laos or Latvia or Lebanon or Lesotho or Basutoland or Liberia or Libya or Lithuania or Macedonia or Madagascar or Malagasy Republic or Malaysia or Malaya or Malay or Sabah or Sarawak or Malawi or Nyasaland or Mali or Malta or Marshall Islands or Mauritania or Mauritius or Agalega Islands or Mexico or Micronesia or Middle East or Moldova or Moldovia or Moldovian or Mongolia or Montenegro or Morocco or Ifni or Mozambique or Myanmar or Myanma or Burma or Namibia or Nepal or Netherlands Antilles or New Caledonia or Nicaragua or Niger or Nigeria or Northern Mariana Islands or Oman or Muscat or Pakistan or Palau or Palestine or Panama or Paraguay or Peru or Philippines or Philipines or Phillipines or Phillippines or Poland or Portugal or Puerto Rico or Romania or Rumania or Roumania or Russia or Russian or Rwanda or Ruanda or Saint Kitts or St Kitts or Nevis or Saint Lucia or St Lucia or Saint Vincent or St Vincent or Grenadines or Samoa or Samoan Islands or Navigator Island or Navigator Islands or Sao Tome or Saudi Arabia or Senegal or Serbia or Montenegro or Seychelles or Sierra Leone or Slovenia or Sri Lanka or Ceylon or Solomon Islands or Somalia or South Africa or Sudan or Suriname or Surinam or Swaziland or Syria or Tajikistan or Tadzhikistan or Tadjikistan or Tadzhik or Tanzania or Thailand or Togo or Togolese Republic or Tonga or Trinidad or Tobago or Tunisia or Turkey or Turkmenistan or Turkmen or Uganda or Ukraine or Uruguay or USSR or Soviet Union or Union of Soviet Socialist Republics or Uzbekistan or Uzbek or Vanuatu or New Hebrides or Venezuela or Vietnam or Viet Nam or West Bank or Yemen or Yugoslavia or Zambia or Zimbabwe or Rhodesia).hw,kf,ti,ab,cp. ((developing or less* developed or under developed or underdeveloped or middle income or low* income or underserved or under served or deprived or poor* or foreign) adj (countr* or nation? or population? or world or region*)).ti,ab. ((developing or less* developed or under developed or underdeveloped or middle income or low* income) adj (economy or economies)).ti,ab. (lmic or lmics or third world or lami countr*).ti,ab. transitional countr*.ti,ab.or/1‐8
	juvenile delinquency.sh.
	(juvenile adj1 delinquen*).mp.
	"Child Behavior Disorders".sh.
	(school adj1 violence).mp.
	(childhood adj1 externalizing adj1 behavio?r).mp.
	or/
	Social Behavior Disorders.sh.
	conduct disorder.sh.
	(conduct adj1 disorder*).mp.
	aggression.sh.
	aggress*.mp.
	(acting adj1 out).mp.
	(aggressive adj1 behavio?r).mp.
	(behavio?r* adj1 problem*).mp.
	(behavio?r* adj1 disorder*).mp.
	(conduct adj1 problem*).mp.
	(conduct adj1 disorder*).mp.
	(impulse adj1 control adj1 disorder*).mp.
	(antisocial adj1 behavio?r*).mp.
	(anti‐social adj1 behavio?r*).mp.
	(oppositional adj1 defiant adj1 disorder*).af.
	(disruptive adj1 behavio?r adj1 disorder*).af.
	violen*.mp.
	(violent adj1 crime*).mp.
	exp crime/
	crime.mp.
	crimes.mp.
	criminal*.mp.
	(criminal behavio?r*).mp.
	bully*.mp
	bullying.sh.
	gang.mp.
	gangs.mp.
	homicid*.mp.
	homicide.sh.
	(externalizing adj1 behavio?r adj1 problem*).mp.
	externalizing.mp.
	externalising.mp.
	externalized.mp.
	externalised.mp.
	externaliz*.mp.
	externalis*.mp.
	(externalizing adj1 behavio?r).mp.
	or/14‐
	11 or 12 or 13 or 14 or 15 or 16 or 17 or 18 or 19 or 20 or 21 or 22 or 23 or 24 or 25
	9 and 26
	exp child/
	"Child Health Services".sh.
	"Child Welfare".sh.
	"Child Behavior".sh.
	"Child Care".sh.
	“Child Development".sh.
	Infant.sh.
	baby.ti,ab.
	babies.ti,ab.
	toddler.ti,ab.
	toddlers.ti,ab.
	adolescen*.ti,ab.
	adolescent.ti,ab.
	adolescents.ti,ab.
	adolescence.ti,ab.
	child*.ti,ab.
	child.ti,ab.
	children*.ti,ab.
	childhood*.ti,ab.
	childhood.ti,ab.
	youth*.ti,ab.
	youth.ti,ab.
	youths.ti,ab.
	student*.ti,ab.
	student.ti,ab.
	students.ti,ab.
	teen*.ti,ab.
	teenager.ti,ab.
	teenagers.ti,ab.
	boy.ti,ab.
	boys.ti,ab.
	girl.ti,ab.
	girls.ti,ab.
	pupil.ti,ab.
	pupils.ti,ab.
	pupil*.ti,ab.
	youngster*.ti,ab.
	youngster.ti,ab.
	youngsters.ti,ab.
	juvenile*.ti,ab.
	juvenile.ti,ab.
	juveniles.ti,ab.
	Infant*.ti,ab.
	infant.ti,ab.
	infants.ti,ab.
	(young adj1 adult*).ti,ab.
	27 or 28 or 29 or 30 or 31 or 32 or 33 or 34 or 35 or 36 or 37 or 38 or 39 26 and 40
**EMBASE (Ovid)** 1974 to 2013 Using EMTREE	Exp developing country/ (Developing adj1 Countr*).hw,ti,ab,cp. (Africa or Central Africa or Latin America or Caribbean or West Indies or Eastern Europe or Soviet or South America or Arab or Middle East or Latin America or Central America).hw,ti,ab,cp. (Afghanistan or Albania or Algeria or Angola or Antigua or Barbuda or Argentina or Armenia or Armenian or Aruba or Azerbaijan or Bahrain or Bangladesh or Barbados or Benin or Byelarus or Byelorussian or Belarus or Belorussian or Belorussia or Belize or Bhutan or Bolivia or Bosnia or Herzegovina or Hercegovina or Botswana or Brasil or Brazil or Bulgaria or Burkina Faso or Burkina Fasso or Upper Volta or Burundi or Urundi or Cambodia or Khmer Republic or Kampuchea or Cameroon or Cameroons or Cameron or Camerons or Cape Verde or Central African Republic or Chad or Chile or China or Colombia or Comoros or Comoro Islands or Comores or Mayotte or Congo or Zaire or Costa Rica or Cote d'Ivoire or Ivory Coast or Croatia or Cuba or Cyprus or Czechoslovakia or Czech Republic or Slovakia or Slovak Republic or Djibouti or French Somaliland or Dominica or Dominican Republic or East Timor or East Timur or Timor Leste or Ecuador or Egypt or United Arab Republic or El Salvador or Eritrea or Estonia or Ethiopia or Fiji or Gabon or Gabonese Republic or Gambia or Gaza or Georgia Republic or Georgian Republic or Ghana or Gold Coast or Greece or Grenada or Guatemala or Guinea or Guam or Guiana or Guyana or Haiti or Honduras or Hungary or India or Maldives or Indonesia or Iran or Iraq or Isle of Man or Jamaica or Jordan or Kazakhstan or Kazakh or Kenya or Kiribati or Korea or Kosovo or Kyrgyzstan or Kirghizia or Kyrgyz Republic or Kirghiz or Kirgizstan or Lao PDR or Laos or Latvia or Lebanon or Lesotho or Basutoland or Liberia or Libya or Lithuania or Macedonia or Madagascar or Malagasy Republic or Malaysia or Malaya or Malay or Sabah or Sarawak or Malawi or Nyasaland or Mali or Malta or Marshall Islands or Mauritania or Mauritius or Agalega Islands or Mexico or Micronesia or Middle East or Moldova or Moldovia or Moldovian or Mongolia or Montenegro or Morocco or Ifni or Mozambique or Myanmar or Myanma or Burma or Namibia or Nepal or Netherlands Antilles or New Caledonia or Nicaragua or Niger or Nigeria or Northern Mariana Islands or Oman or Muscat or Pakistan or Palau or Palestine or Panama or Paraguay or Peru or Philippines or Philipines or Phillipines or Phillippines or Poland or Portugal or Puerto Rico or Romania or Rumania or Roumania or Russia or Russian or Rwanda or Ruanda or Saint Kitts or St Kitts or Nevis or Saint Lucia or St Lucia or Saint Vincent or St Vincent or Grenadines or Samoa or Samoan Islands or Navigator Island or Navigator Islands or Sao Tome or Saudi Arabia or Senegal or Serbia or Montenegro or Seychelles or Sierra Leone or Slovenia or Sri Lanka or Ceylon or Solomon Islands or Somalia or South Africa or Sudan or Suriname or Surinam or Swaziland or Syria or Tajikistan or Tadzhikistan or Tadjikistan or Tadzhik or Tanzania or Thailand or Togo or Togolese Republic or Tonga or Trinidad or Tobago or Tunisia or Turkey or Turkmenistan or Turkmen or Uganda or Ukraine or Uruguay or USSR or Soviet Union or Union of Soviet Socialist Republics or Uzbekistan or Uzbek or Vanuatu or New Hebrides or Venezuela or Vietnam or Viet Nam or West Bank or Yemen or Yugoslavia or Zambia or Zimbabwe or Rhodesia).hw,ti,ab,cp. ((developing or less* developed or under developed or underdeveloped or middle income or low* income or underserved or under served or deprived or poor* or foreign) adj1 (countr* or nation? or population? or world or region*)).ti,ab. ((developing or less* developed or under developed or underdeveloped or middle income or low* income) adj1 (economy or economies)).ti,ab. (low adj3 middle adj1 countr*).ti,ab.(lmic or lmics or third world or lami countr*).ti,ab. (transitional countr*).ti,ab. or/1‐8
	exp delinquency/(juvenile adj1 delinquen*).mp. (school adj1 violence).mp.
	**or/**
	(conduct adj1 problem*).mp.
	(conduct adj1 disorder*).mp.
	(behavio?r* adj1 problem*).mp.
	(behavio?r adj1 disorder*).mp.
	(oppositional adj1 defiant adj1 disorder*).af.
	(disruptive adj1 behavio?r adj1 disorder*).af.
	(impulse adj1 control adj1 disorder*).mp.
	(criminal adj1 behavio?r*).mp.
	(violent adj1 crime*).mp.
	homicid*.mp.
	homicide.mp.
	homicides.mp.
	conduct disorder/
	aggression.mp.
	aggressive.mp.
	aggress*.mp.
	violen*.mp.
	violent.mp.
	violence.mp.
	crime.mp.
	crimes.mp
	criminal*.mp.
	gang.mp.
	gangs.mp.
	bully*.mp.
	bully.mp.
	bullying.mp.
	(aggressive adj1 behavio?r).mp.
	(antisocial adj1 behavio?r).mp.
	(anti‐social adj1 behavio?r*).mp.
	exp aggression/
	homicide/
	gang/
	crime/
	criminal behavior/
	abnormal behavior/
	behavior disorder/
	disruptive behaviour/
	criminology/
	homicide/
	acting out/
	violence/
	workplace violence/
	impulse control disorder/
	oppositional defiant disorder/
	conduct disorder/
	(externalizing adj1 behavio?r adj1 problem*).mp.
	(externalizing adj1 behavio?r).mp.
	(externalising adj1 behavio?r).mp.
	externalizing.mp.
	externalising.mp.
	externalized.mp.
	externalised.mp.
	externaliz*.mp.
	externalis*.mp.
	11 or 12 or 13 or 14 or 15 or 16 or 17 or 18 or 19 or 20 or 21 or 22 or 23 or 24 or 25
	9 and 26
	exp child/
	adolescent.sh.
	Infant.sh.
	baby.ti,ab.
	babies.ti,ab.
	toddler.ti,ab.
	toddlers.ti,ab.
	adolescen*.ti,ab.
	adolescent.ti,ab.
	adolescents.ti,ab.
	adolescence.ti,ab.
	child*.ti,ab.
	child.ti,ab.
	children*.ti,ab.
	childhood*.ti,ab.
	childhood.ti,ab.
	youth*.ti,ab.
	youth.ti,ab.
	youths.ti,ab.
	student*.ti,ab.
	students.ti,ab.
	student.ti,ab.
	teen*.ti,ab.
	teenager.ti,ab.
	teenagers.ti,ab.
	boy.ti,ab.
	boys.ti,ab.
	girl.ti,ab.
	girls.ti,ab.
	pupil.ti,ab.
	pupils.ti,ab.
	pupil*.ti,ab.
	youngster*.ti,ab.
	youngster.ti,ab.
	youngsters.ti,ab.
	juvenile*.ti,ab.
	juvenile.ti,ab.
	juveniles.ti,ab.
	Infant*.ti,ab.
	infant.ti,ab.
	infants.ti,ab.
	(young adj1 adult*).ti,ab.
	57‐27 or 28 or 29 or 30 or 31 or 32 or 33 or 34 or 35 or 36 or 37 or 38 or 39
	26 and 40
**CINAHL (EBSCO)**	TI (“developing country” or “developing countries” or “developing nation” or “developing nations” or less* W1 “developed country” or less* W1 “developed countries” or less* W1 “developed nation” or less* W1 “developed nations” or “third world” or “under developed” or “middle income” or “low income” or “underserved country” or “underserved countries” or “underserved nation” or “underserved nations” or “under served country” or “under served countries” or “under served nation” or “under served nations” or “underserved population” or “underserved populations” or “under served population” or “under served populations” or “deprived country” or “deprived countries” or “deprived nation” or “deprived nations” or poor* W1 country or poor* W1 countries or poor* W1 nation* or poor* W1 population* or lmic or lmics) AB (“developing country” or “developing countries” or “developing nation” or “developing nations” or less* W1 “developed country” or less* W1 “developed countries” or less* W1 “developed nation” or less* W1 “developed nations” or “third world” or “under developed” or “middle income” or “low income” or “underserved country” or “underserved countries” or “underserved nation” or “underserved nations” or “under served country” or “under served countries” or “under served nation” or “under served nations” or “underserved population” or “underserved populations” or “under served population” or “under served populations” or “deprived country” or “deprived countries” or “deprived nation” or “deprived nations” or poor* W1 country or poor* W1 countries or poor* W1 nation* or poor* W1 population* or lmic or lmics) MW (Afghanistan or Bangladesh or Benin or “Burkina Faso” or Burundi or Cambodia or “Central African Republic” or Chad or Comoros or Congo or “Cote d'Ivoire” or Eritrea or Ethiopia or Gambia or Ghana or Guinea or Haiti or India or Kenya or Korea or Kyrgyz or Kyrgyzstan or Lao or Laos or Liberia or Madagascar or Malawi or Mali or Mauritania or Melanesia or Mongolia or Mozambique or Burma or Myanmar or Nepal or Niger or Nigeria or Pakistan or Rwanda or “Salomon Islands” or “Sao Tome” or Senegal or “Sierra Leone” or Somalia or Sudan or Tajikistan or Tanzania or Timor or Togo or Uganda or Uzbekistan or Vietnam or “Viet Nam” or Yemen or Zambia or Zimbabwe) TI (Afghanistan or Bangladesh or Benin or “Burkina Faso” or Burundi or Cambodia or “Central African Republic” or Chad or Comoros or Congo or “Cote d'Ivoire” or Eritrea or Ethiopia or Gambia or Ghana or Guinea or Haiti or India or Kenya or Korea or Kyrgyz or Kyrgyzstan or Lao or Laos or Liberia or Madagascar or Malawi or Mali or Mauritania or Melanesia or Mongolia or Mozambique or Burma or Myanmar or Nepal or Niger or Nigeria or Pakistan or Rwanda or “Salomon Islands” or “Sao Tome” or Senegal or “Sierra Leone” or Somalia or Sudan or Tajikistan or Tanzania or Timor or Togo or Uganda or Uzbekistan or Vietnam or “Viet Nam” or Yemen or Zambia or Zimbabwe) AB (Afghanistan or Bangladesh or Benin or “Burkina Faso” or Burundi or Cambodia or “Central African Republic” or Chad or Comoros or Congo or “Cote d'Ivoire” or Eritrea or Ethiopia or Gambia or Ghana or Guinea or Haiti or India or Kenya or Korea or Kyrgyz or Kyrgyzstan or Lao or Laos or Liberia or Madagascar or Malawi or Mali or Mauritania or Melanesia or Mongolia or Mozambique or Burma or Myanmar or Nepal or Niger or Nigeria or Pakistan or Rwanda or “Salomon Islands” or “Sao Tome” or Senegal or “Sierra Leone” or Somalia or Sudan or Tajikistan or Tanzania or Timor or Togo or Uganda or Uzbekistan or Vietnam or “Viet Nam” or Yemen or Zambia or Zimbabwe) MW (Albania or Algeria or Angola or Armenia or Azerbaijan or Belarus or Bhutan or Bolivia or Bosnia or Herzegovina or “Cape Verde” or Cameroon or China or Colombia or Congo or Cuba or Djibouti or “Dominican Republic” or Ecuador or Egypt or “El Salvador” or Fiji or Gaza or Georgia or Guam or Guatemala or Guyana or Honduras or “Indian Ocean Islands” or Indonesia or Iran or Iraq or Jamaica or Jordan or Kiribati or Lesotho or Macedonia or Maldives or “Marshall Islands” or Micronesia or “Middle East” or Moldova or Morocco or Namibia or Nicaragua or Palestin* or Paraguay or Peru or Philippines or Samoa or “Sri Lanka” or Suriname or Swaziland or Syria or “Syrian Arab Republic” or Thailand or Tonga or Tunisia or Turkmenistan or Ukraine or Vanuatu or “West Bank”) or TI (Albania or Algeria or Angola or Armenia or Azerbaijan or Belarus or Bhutan or Bolivia or Bosnia or Herzegovina or “Cape Verde” or Cameroon or China or Colombia or Congo or Cuba or Djibouti or “Dominican Republic” or Ecuador or Egypt or “El Salvador” or Fiji or Gaza or Georgia or Guam or Guatemala or Guyana or Honduras or “Indian Ocean Islands” or Indonesia or Iran or Iraq or Jamaica or Jordan or Kiribati or Lesotho or Macedonia or Maldives or “Marshall Islands” or Micronesia or “Middle East” or Moldova or Morocco or Namibia or Nicaragua or Palestin* or Paraguay or Peru or Philippines or Samoa or “Sri Lanka” or Suriname or Swaziland or Syria or “Syrian Arab Republic” or Thailand or Tonga or Tunisia or Turkmenistan or Ukraine or Vanuatu or “West Bank” Albania or Algeria or Angola or Armenia or Azerbaijan or Belarus or Bhutan or Bolivia or Bosnia or Herzegovina or “Cape Verde” or Cameroon or China or Colombia or Congo or Cuba or Djibouti or “Dominican Republic” or Ecuador or Egypt or “El Salvador” or Fiji or Gaza or Georgia or Guam or Guatemala or Guyana or Honduras or “Indian Ocean Islands” or Indonesia or Iran or Iraq or Jamaica or Jordan or Kiribati or Lesotho or Macedonia or Maldives or “Marshall Islands” or Micronesia or “Middle East” or Moldova or Morocco or Namibia or Nicaragua or Palestin* or Paraguay or Peru or Philippines or Samoa or “Sri Lanka” or Suriname or Swaziland or Syria or “Syrian Arab Republic” or Thailand or Tonga or Tunisia or Turkmenistan or Ukraine or Vanuatu or “West Bank”) AB (Albania or Algeria or Angola or Armenia or Azerbaijan or Belarus or Bhutan or Bolivia or Bosnia or Herzegovina or “Cape Verde” or Cameroon or China or Colombia or Congo or Cuba or Djibouti or “Dominican Republic” or Ecuador or Egypt or “El Salvador” or Fiji or Gaza or Georgia or Guam or Guatemala or Guyana or Honduras or “Indian Ocean Islands” or Indonesia or Iran or Iraq or Jamaica or Jordan or Kiribati or Lesotho or Macedonia or Maldives or “Marshall Islands” or Micronesia or “Middle East” or Moldova or Morocco or Namibia or Nicaragua or Palestin* or Paraguay or Peru or Philippines or Samoa or “Sri Lanka” or Suriname or Swaziland or Syria or “Syrian Arab Republic” or Thailand or Tonga or Tunisia or Turkmenistan or Ukraine or Vanuatu or “West Bank”) MW (“American Samoa” or Argentina or Belize or Botswana or Brazil or Bulgaria or Chile or Comoros or “Costa Rica” or Croatia or Dominica or Guinea or Gabon or Grenada or Grenadines or Hungary or Kazakhstan or Latvia or Lebanon or Libia or libyan or Libya or Lithuania or Malaysia or Mauritius or Mayotte or Mexico or Micronesia or Montenegro or Nevis or “Northern Mariana Islands” or Oman or Palau or Panama or Poland or Romania or Russia or “Russian Federation” or Samoa or “Saint Lucia” or “St Lucia” or “Saint Kitts” or “St Kitts” or “Saint Vincent” or “St Vincent” or Serbia or Seychelles or Slovakia or “Slovak Republic” or “South Africa” or Turkey or Uruguay or Venezuela or Yugoslavia) TI (“American Samoa” or Argentina or Belize or Botswana or Brazil or Bulgaria or Chile or Comoros or “Costa Rica” or Croatia or Dominica or Guinea or Gabon or Grenada or Grenadines or Hungary or Kazakhstan or Latvia or Lebanon or Libia or libyan or Libya or Lithuania or Malaysia or Mauritius or Mayotte or Mexico or Micronesia or Montenegro or Nevis or “Northern Mariana Islands” or Oman or Palau or Panama or Poland or Romania or Russia or “Russian Federation” or Samoa or “Saint Lucia” or “St Lucia” or “Saint Kitts” or “St Kitts” or “Saint Vincent” or “St Vincent” or Serbia or Seychelles or Slovakia or “Slovak Republic” or “South Africa” or Turkey or Uruguay or Venezuela or Yugoslavia) AB (“American Samoa” or Argentina or Belize or Botswana or Brazil or Bulgaria or Chile or Comoros or “Costa Rica” or Croatia or Dominica or Guinea or Gabon or Grenada or Grenadines or Hungary or Kazakhstan or Latvia or Lebanon or Libia or libyan or Libya or Lithuania or Malaysia or Mauritius or Mayotte or Mexico or Micronesia or Montenegro or Nevis or “Northern Mariana Islands” or Oman or Palau or Panama or Poland or Romania or Russia or “Russian Federation” or Samoa or “Saint Lucia” or “St Lucia” or “Saint Kitts” or “St Kitts” or “Saint Vincent” or “St Vincent” or Serbia or Seychelles or Slovakia or “Slovak Republic” or “South Africa” or Turkey or Uruguay or Venezuela or Yugoslavia)
	TI (Africa or Asia or “South America” or “Latin America” or “Central America”)
	AB (Africa or Asia or “South America” or “Latin America” or “Central America”)
	(MH “Asia+”)
	(MH “West Indies+”)
	(MH “South America+”)
	(MH “Latin America”)
	(MH “Central America+”)
	(MH “Africa+”)
	(MH “Developing Countries”)
	or/
	(MH "Juvenile Delinquency")
	AB (juvenile N1 delinquen*)
	AB (school N1 violence)
	(MH "Juvenile Offenders+")
	(MH "Child Behavior Disorders")
	or/
	(MH "Aggression")
	(MH "Social Behavior Disorders")
	(MH "Crime")
	(MH "Violence")
	(MH "Homicide")
	(MH "Assault and Battery")
	(MH "Aggression+")
	AB (conduct N1 problem*)
	AB (behavio#r N1 problem*)
	AB (antisocial N1 behavio#r)
	AB (disruptive N1 behavio#r)
	AB (conduct N1 disorder*)
	AB (behavio#r N1 disorder*)
	AB (aggressive N1 behavio#r)
	AB (aggression)
	AB (aggressive)
	AB (antisocial N1 behavio#r)
	AB (anti‐social N1 behavio#r)
	AB (gang)
	AB (gangs)
	AB (criminal N1 behavio#r)
	AB (violent N1 crime)
	AB (homicid*)
	AB (violence)
	AB (violent)
	AB (crime)
	AB (crimes)
	AB (criminal*)
	AB (bully)
	AB (bullying)
	AB (delinquent*)
	AB (delinquenc*)
	TX (oppositional N1 defiant N1 disorder*)
	TX (disruptive N1 behavio#r N1 disorder*)
	AB (externalizing N1 behavio#r N1 problem*)
	AB (externalizing)
	AB (externalising)
	AB (externalized)
	AB (externalised)
	AB (externaliz*)
	AB (externalis*)
	AB (externalizing N1 behavio#r)
	AB (externalising N1 behavio#r)
	or /…
	S21 AND S68
	(MH " Child+")
	(MH "Adolescence")
	AB (Adolescen*)
	AB (Adolescence)
	AB (Adolescent)
	AB (adolescents)
	AB (Child*)
	AB (child)
	AB (children)
	AB (childhood)
	AB (youth*)
	AB (youth)
	AB (youths)
	AB (student*)
	AB (Students)
	AB (Student)
	AB (teen*)
	AB (teenager)
	AB (teenagers)
	AB (boy*)
	AB (boy)
	AB (boys)
	AB (girl*)
	AB (girl)
	AB (girls)
	AB (pupil)
	AB (pupils)
	AB (pupil*)
	AB (youngster*)
	AB (youngster)
	AB (youngsters)
	AB (juvenile*)
	AB (juvenile)
	AB (juveniles)
	AB (young N1 adult*)
	AB (infant*)
	AB (infants)
	AB (infant)
	AB (baby*)
	AB (baby)
	AB (babies)
	AB (toddler)
	AB (toddler*)
	AB (toddlers)
	or/
	22 and 38
**Criminal Justice Abstracts (EBSCOHost)**	TI (“developing country” or “developing countries” or “developing nation” or “developing nations” or less* W1 “developed country” or less* W1 “developed countries” or less* W1 “developed nation” or less* W1 “developed nations” or “third world” or “under developed” or “middle income” or “low income” or “underserved country” or “underserved countries” or “underserved nation” or “underserved nations” or “under served country” or “under served countries” or “under served nation” or “under served nations” or “underserved population” or “underserved populations” or “under served population” or “under served populations” or “deprived country” or “deprived countries” or “deprived nation” or “deprived nations” or poor* W1 country or poor* W1 countries or poor* W1 nation* or poor* W1 population* or lmic or lmics) AB (“developing country” or “developing countries” or “developing nation” or “developing nations” or less* W1 “developed country” or less* W1 “developed countries” or less* W1 “developed nation” or less* W1 “developed nations” or “third world” or “under developed” or “middle income” or “low income” or “underserved country” or “underserved countries” or “underserved nation” or “underserved nations” or “under served country” or “under served countries” or “under served nation” or “under served nations” or “underserved population” or “underserved populations” or “under served population” or “under served populations” or “deprived country” or “deprived countries” or “deprived nation” or “deprived nations” or poor* W1 country or poor* W1 countries or poor* W1 nation* or poor* W1 population* or lmic or lmics) MW (Afghanistan or Bangladesh or Benin or “Burkina Faso” or Burundi or Cambodia or “Central African Republic” or Chad or Comoros or Congo or “Cote d'Ivoire” or Eritrea or Ethiopia or Gambia or Ghana or Guinea or Haiti or India or Kenya or Korea or Kyrgyz or Kyrgyzstan or Lao or Laos or Liberia or Madagascar or Malawi or Mali or Mauritania or Melanesia or Mongolia or Mozambique or Burma or Myanmar or Nepal or Niger or Nigeria or Pakistan or Rwanda or “Salomon Islands” or “Sao Tome” or Senegal or “Sierra Leone” or Somalia or Sudan or Tajikistan or Tanzania or Timor or Togo or Uganda or Uzbekistan or Vietnam or “Viet Nam” or Yemen or Zambia or Zimbabwe) TI (Afghanistan or Bangladesh or Benin or “Burkina Faso” or Burundi or Cambodia or “Central African Republic” or Chad or Comoros or Congo or “Cote d'Ivoire” or Eritrea or Ethiopia or Gambia or Ghana or Guinea or Haiti or India or Kenya or Korea or Kyrgyz or Kyrgyzstan or Lao or Laos or Liberia or Madagascar or Malawi or Mali or Mauritania or Melanesia or Mongolia or Mozambique or Burma or Myanmar or Nepal or Niger or Nigeria or Pakistan or Rwanda or “Salomon Islands” or “Sao Tome” or Senegal or “Sierra Leone” or Somalia or Sudan or Tajikistan or Tanzania or Timor or Togo or Uganda or Uzbekistan or Vietnam or “Viet Nam” or Yemen or Zambia or Zimbabwe) AB (Afghanistan or Bangladesh or Benin or “Burkina Faso” or Burundi or Cambodia or “Central African Republic” or Chad or Comoros or Congo or “Cote d'Ivoire” or Eritrea or Ethiopia or Gambia or Ghana or Guinea or Haiti or India or Kenya or Korea or Kyrgyz or Kyrgyzstan or Lao or Laos or Liberia or Madagascar or Malawi or Mali or Mauritania or Melanesia or Mongolia or Mozambique or Burma or Myanmar or Nepal or Niger or Nigeria or Pakistan or Rwanda or “Salomon Islands” or “Sao Tome” or Senegal or “Sierra Leone” or Somalia or Sudan or Tajikistan or Tanzania or Timor or Togo or Uganda or Uzbekistan or Vietnam or “Viet Nam” or Yemen or Zambia or Zimbabwe) MW (Albania or Algeria or Angola or Armenia or Azerbaijan or Belarus or Bhutan or Bolivia or Bosnia or Herzegovina or “Cape Verde” or Cameroon or China or Colombia or Congo or Cuba or Djibouti or “Dominican Republic” or Ecuador or Egypt or “El Salvador” or Fiji or Gaza or Georgia or Guam or Guatemala or Guyana or Honduras or “Indian Ocean Islands” or Indonesia or Iran or Iraq or Jamaica or Jordan or Kiribati or Lesotho or Macedonia or Maldives or “Marshall Islands” or Micronesia or “Middle East” or Moldova or Morocco or Namibia or Nicaragua or Palestin* or Paraguay or Peru or Philippines or Samoa or “Sri Lanka” or Suriname or Swaziland or Syria or “Syrian Arab Republic” or Thailand or Tonga or Tunisia or Turkmenistan or Ukraine or Vanuatu or “West Bank”) or TI (Albania or Algeria or Angola or Armenia or Azerbaijan or Belarus or Bhutan or Bolivia or Bosnia or Herzegovina or “Cape Verde” or Cameroon or China or Colombia or Congo or Cuba or Djibouti or “Dominican Republic” or Ecuador or Egypt or “El Salvador” or Fiji or Gaza or Georgia or Guam or Guatemala or Guyana or Honduras or “Indian Ocean Islands” or Indonesia or Iran or Iraq or Jamaica or Jordan or Kiribati or Lesotho or Macedonia or Maldives or “Marshall Islands” or Micronesia or “Middle East” or Moldova or Morocco or Namibia or Nicaragua or Palestin* or Paraguay or Peru or Philippines or Samoa or “Sri Lanka” or Suriname or Swaziland or Syria or “Syrian Arab Republic” or Thailand or Tonga or Tunisia or Turkmenistan or Ukraine or Vanuatu or “West Bank” Albania or Algeria or Angola or Armenia or Azerbaijan or Belarus or Bhutan or Bolivia or Bosnia or Herzegovina or “Cape Verde” or Cameroon or China or Colombia or Congo or Cuba or Djibouti or “Dominican Republic” or Ecuador or Egypt or “El Salvador” or Fiji or Gaza or Georgia or Guam or Guatemala or Guyana or Honduras or “Indian Ocean Islands” or Indonesia or Iran or Iraq or Jamaica or Jordan or Kiribati or Lesotho or Macedonia or Maldives or “Marshall Islands” or Micronesia or “Middle East” or Moldova or Morocco or Namibia or Nicaragua or Palestin* or Paraguay or Peru or Philippines or Samoa or “Sri Lanka” or Suriname or Swaziland or Syria or “Syrian Arab Republic” or Thailand or Tonga or Tunisia or Turkmenistan or Ukraine or Vanuatu or “West Bank”) AB (Albania or Algeria or Angola or Armenia or Azerbaijan or Belarus or Bhutan or Bolivia or Bosnia or Herzegovina or “Cape Verde” or Cameroon or China or Colombia or Congo or Cuba or Djibouti or “Dominican Republic” or Ecuador or Egypt or “El Salvador” or Fiji or Gaza or Georgia or Guam or Guatemala or Guyana or Honduras or “Indian Ocean Islands” or Indonesia or Iran or Iraq or Jamaica or Jordan or Kiribati or Lesotho or Macedonia or Maldives or “Marshall Islands” or Micronesia or “Middle East” or Moldova or Morocco or Namibia or Nicaragua or Palestin* or Paraguay or Peru or Philippines or Samoa or “Sri Lanka” or Suriname or Swaziland or Syria or “Syrian Arab Republic” or Thailand or Tonga or Tunisia or Turkmenistan or Ukraine or Vanuatu or “West Bank”) MW (“American Samoa” or Argentina or Belize or Botswana or Brazil or Bulgaria or Chile or Comoros or “Costa Rica” or Croatia or Dominica or Guinea or Gabon or Grenada or Grenadines or Hungary or Kazakhstan or Latvia or Lebanon or Libia or libyan or Libya or Lithuania or Malaysia or Mauritius or Mayotte or Mexico or Micronesia or Montenegro or Nevis or “Northern Mariana Islands” or Oman or Palau or Panama or Poland or Romania or Russia or “Russian Federation” or Samoa or “Saint Lucia” or “St Lucia” or “Saint Kitts” or “St Kitts” or “Saint Vincent” or “St Vincent” or Serbia or Seychelles or Slovakia or “Slovak Republic” or “South Africa” or Turkey or Uruguay or Venezuela or Yugoslavia) TI (“American Samoa” or Argentina or Belize or Botswana or Brazil or Bulgaria or Chile or Comoros or “Costa Rica” or Croatia or Dominica or Guinea or Gabon or Grenada or Grenadines or Hungary or Kazakhstan or Latvia or Lebanon or Libia or libyan or Libya or Lithuania or Malaysia or Mauritius or Mayotte or Mexico or Micronesia or Montenegro or Nevis or “Northern Mariana Islands” or Oman or Palau or Panama or Poland or Romania or Russia or “Russian Federation” or Samoa or “Saint Lucia” or “St Lucia” or “Saint Kitts” or “St Kitts” or “Saint Vincent” or “St Vincent” or Serbia or Seychelles or Slovakia or “Slovak Republic” or “South Africa” or Turkey or Uruguay or Venezuela or Yugoslavia) AB (“American Samoa” or Argentina or Belize or Botswana or Brazil or Bulgaria or Chile or Comoros or “Costa Rica” or Croatia or Dominica or Guinea or Gabon or Grenada or Grenadines or Hungary or Kazakhstan or Latvia or Lebanon or Libia or libyan or Libya or Lithuania or Malaysia or Mauritius or Mayotte or Mexico or Micronesia or Montenegro or Nevis or “Northern Mariana Islands” or Oman or Palau or Panama or Poland or Romania or Russia or “Russian Federation” or Samoa or “Saint Lucia” or “St Lucia” or “Saint Kitts” or “St Kitts” or “Saint Vincent” or “St Vincent” or Serbia or Seychelles or Slovakia or “Slovak Republic” or “South Africa” or Turkey or Uruguay or Venezuela or Yugoslavia)
	TI (Africa or Asia or “South America” or “Latin America” or “Central America”)
	AB (Africa or Asia or “South America” or “Latin America” or “Central America”)
	(MH “Asia+”)
	(MH “West Indies+”)
	(MH “South America+”)
	(MH “Latin America”)
	(MH “Central America+”)
	(MH “Africa+”)
	(MH “Developing Countries”)
	**or/**
	(MH "Juvenile Delinquency")
	AB (juvenile N1 delinquen*)
	AB (school N1 violence)
	(MH "Juvenile Offenders+")
	(MH "Child Behavior Disorders")
	**or/**
	(MH "Aggression")
	(MH "Social Behavior Disorders")
	(MH "Crime")
	(MH "Violence")
	(MH "Homicide")
	(MH "Assault and Battery")
	(MH "Aggression+")
	AB (conduct N1 problem*)
	AB (behavio#r N1 problem*)
	AB (disruptive N1 behavio#r)
	AB (conduct N1 disorder*)
	AB (behavio#r N1 disorder*)
	AB (aggressive N1 behavio#r)
	AB (aggression)
	AB (aggressive)
	AB (antisocial N1 behavio#r)
	AB (anti‐social N1 behavio#r)
	AB (gang)
	AB (gangs)
	AB (criminal N1 behavio#r)
	AB (violent N1 crime)
	AB (homicid*)
	AB (violence)
	AB (violent)
	AB (crime)
	AB (crimes)
	AB (criminal*)
	AB (bully)
	AB (bullying)
	AB (delinquent*)
	AB (delinquenc*)
	TX (oppositional N1 defiant N1 disorder*)
	TX (disruptive N1 behavio#r N1 disorder*)
	AB (externalizing N1 behavio#r N1 problem*)
	AB (externalizing)
	AB (externalising)
	AB (externalized)
	AB (externalised)
	AB (externaliz*)
	AB (externalis*)
	AB (externalizing N1 behavio#r)
	AB (externalising N1 behavio#r)
	or /…
	S21 AND S68
	(MH " Child+")
	(MH "Adolescence")
	AB (Adolescen*)
	AB (Adolescence)
	AB (Adolescent)
	AB (adolescents)
	AB (Child*)
	AB (child)
	AB (children)
	AB (childhood)
	AB (youth*)
	AB (youth)
	AB (youths)
	AB (student*)
	AB (Students)
	AB (Student)
	AB (teen*)
	AB (teenager)
	AB (teenagers)
	AB (boy*)
	AB (boy)
	AB (boys)
	AB (girl*)
	AB (girl)
	AB (girls)
	AB (pupil)
	AB (pupils)
	AB (pupil*)
	AB (youngster*)
	AB (youngster)
	AB (youngsters)
	AB (juvenile*)
	AB (juvenile)
	AB (juveniles)
	AB (young N1 adult*)
	AB (infant*)
	AB (infants)
	AB (infant)
	AB (baby*)
	AB (baby)
	AB (babies)
	AB (toddler)
	AB (toddler*)
	AB (toddlers)
	or/
	22 and 38
Russian Academy of Sciences Bibliographies (EBSCOHost)	Same as EconLit
**EconLit (EBSCOhost)**	TI (“developing country” or “developing countries” or “developing nation” or “developing nations” or less* W1 “developed country” or less* W1 “developed countries” or less* W1 “developed nation” or less* W1 “developed nations” or “third world” or “under developed” or “middle income” or “low income” or “underserved country” or “underserved countries” or “underserved nation” or “underserved nations” or “under served country” or “under served countries” or “under served nation” or “under served nations” or “underserved population” or “underserved populations” or “under served population” or “under served populations” or “deprived country” or “deprived countries” or “deprived nation” or “deprived nations” or poor* W1 country or poor* W1 countries or poor* W1 nation* or poor* W1 population* or lmic or lmics) AB (“developing country” or “developing countries” or “developing nation” or “developing nations” or less* W1 “developed country” or less* W1 “developed countries” or less* W1 “developed nation” or less* W1 “developed nations” or “third world” or “under developed” or “middle income” or “low income” or “underserved country” or “underserved countries” or “underserved nation” or “underserved nations” or “under served country” or “under served countries” or “under served nation” or “under served nations” or “underserved population” or “underserved populations” or “under served population” or “under served populations” or “deprived country” or “deprived countries” or “deprived nation” or “deprived nations” or poor* W1 country or poor* W1 countries or poor* W1 nation* or poor* W1 population* or lmic or lmics) MW (Afghanistan or Bangladesh or Benin or “Burkina Faso” or Burundi or Cambodia or “Central African Republic” or Chad or Comoros or Congo or “Cote d'Ivoire” or Eritrea or Ethiopia or Gambia or Ghana or Guinea or Haiti or India or Kenya or Korea or Kyrgyz or Kyrgyzstan or Lao or Laos or Liberia or Madagascar or Malawi or Mali or Mauritania or Melanesia or Mongolia or Mozambique or Burma or Myanmar or Nepal or Niger or Nigeria or Pakistan or Rwanda or “Salomon Islands” or “Sao Tome” or Senegal or “Sierra Leone” or Somalia or Sudan or Tajikistan or Tanzania or Timor or Togo or Uganda or Uzbekistan or Vietnam or “Viet Nam” or Yemen or Zambia or Zimbabwe) TI (Afghanistan or Bangladesh or Benin or “Burkina Faso” or Burundi or Cambodia or “Central African Republic” or Chad or Comoros or Congo or “Cote d'Ivoire” or Eritrea or Ethiopia or Gambia or Ghana or Guinea or Haiti or India or Kenya or Korea or Kyrgyz or Kyrgyzstan or Lao or Laos or Liberia or Madagascar or Malawi or Mali or Mauritania or Melanesia or Mongolia or Mozambique or Burma or Myanmar or Nepal or Niger or Nigeria or Pakistan or Rwanda or “Salomon Islands” or “Sao Tome” or Senegal or “Sierra Leone” or Somalia or Sudan or Tajikistan or Tanzania or Timor or Togo or Uganda or Uzbekistan or Vietnam or “Viet Nam” or Yemen or Zambia or Zimbabwe) AB (Afghanistan or Bangladesh or Benin or “Burkina Faso” or Burundi or Cambodia or “Central African Republic” or Chad or Comoros or Congo or “Cote d'Ivoire” or Eritrea or Ethiopia or Gambia or Ghana or Guinea or Haiti or India or Kenya or Korea or Kyrgyz or Kyrgyzstan or Lao or Laos or Liberia or Madagascar or Malawi or Mali or Mauritania or Melanesia or Mongolia or Mozambique or Burma or Myanmar or Nepal or Niger or Nigeria or Pakistan or Rwanda or “Salomon Islands” or “Sao Tome” or Senegal or “Sierra Leone” or Somalia or Sudan or Tajikistan or Tanzania or Timor or Togo or Uganda or Uzbekistan or Vietnam or “Viet Nam” or Yemen or Zambia or Zimbabwe) MW (Albania or Algeria or Angola or Armenia or Azerbaijan or Belarus or Bhutan or Bolivia or Bosnia or Herzegovina or “Cape Verde” or Cameroon or China or Colombia or Congo or Cuba or Djibouti or “Dominican Republic” or Ecuador or Egypt or “El Salvador” or Fiji or Gaza or Georgia or Guam or Guatemala or Guyana or Honduras or “Indian Ocean Islands” or Indonesia or Iran or Iraq or Jamaica or Jordan or Kiribati or Lesotho or Macedonia or Maldives or “Marshall Islands” or Micronesia or “Middle East” or Moldova or Morocco or Namibia or Nicaragua or Palestin* or Paraguay or Peru or Philippines or Samoa or “Sri Lanka” or Suriname or Swaziland or Syria or “Syrian Arab Republic” or Thailand or Tonga or Tunisia or Turkmenistan or Ukraine or Vanuatu or “West Bank”) or TI (Albania or Algeria or Angola or Armenia or Azerbaijan or Belarus or Bhutan or Bolivia or Bosnia or Herzegovina or “Cape Verde” or Cameroon or China or Colombia or Congo or Cuba or Djibouti or “Dominican Republic” or Ecuador or Egypt or “El Salvador” or Fiji or Gaza or Georgia or Guam or Guatemala or Guyana or Honduras or “Indian Ocean Islands” or Indonesia or Iran or Iraq or Jamaica or Jordan or Kiribati or Lesotho or Macedonia or Maldives or “Marshall Islands” or Micronesia or “Middle East” or Moldova or Morocco or Namibia or Nicaragua or Palestin* or Paraguay or Peru or Philippines or Samoa or “Sri Lanka” or Suriname or Swaziland or Syria or “Syrian Arab Republic” or Thailand or Tonga or Tunisia or Turkmenistan or Ukraine or Vanuatu or “West Bank” Albania or Algeria or Angola or Armenia or Azerbaijan or Belarus or Bhutan or Bolivia or Bosnia or Herzegovina or “Cape Verde” or Cameroon or China or Colombia or Congo or Cuba or Djibouti or “Dominican Republic” or Ecuador or Egypt or “El Salvador” or Fiji or Gaza or Georgia or Guam or Guatemala or Guyana or Honduras or “Indian Ocean Islands” or Indonesia or Iran or Iraq or Jamaica or Jordan or Kiribati or Lesotho or Macedonia or Maldives or “Marshall Islands” or Micronesia or “Middle East” or Moldova or Morocco or Namibia or Nicaragua or Palestin* or Paraguay or Peru or Philippines or Samoa or “Sri Lanka” or Suriname or Swaziland or Syria or “Syrian Arab Republic” or Thailand or Tonga or Tunisia or Turkmenistan or Ukraine or Vanuatu or “West Bank”) AB (Albania or Algeria or Angola or Armenia or Azerbaijan or Belarus or Bhutan or Bolivia or Bosnia or Herzegovina or “Cape Verde” or Cameroon or China or Colombia or Congo or Cuba or Djibouti or “Dominican Republic” or Ecuador or Egypt or “El Salvador” or Fiji or Gaza or Georgia or Guam or Guatemala or Guyana or Honduras or “Indian Ocean Islands” or Indonesia or Iran or Iraq or Jamaica or Jordan or Kiribati or Lesotho or Macedonia or Maldives or “Marshall Islands” or Micronesia or “Middle East” or Moldova or Morocco or Namibia or Nicaragua or Palestin* or Paraguay or Peru or Philippines or Samoa or “Sri Lanka” or Suriname or Swaziland or Syria or “Syrian Arab Republic” or Thailand or Tonga or Tunisia or Turkmenistan or Ukraine or Vanuatu or “West Bank”) MW (“American Samoa” or Argentina or Belize or Botswana or Brazil or Bulgaria or Chile or Comoros or “Costa Rica” or Croatia or Dominica or Guinea or Gabon or Grenada or Grenadines or Hungary or Kazakhstan or Latvia or Lebanon or Libia or libyan or Libya or Lithuania or Malaysia or Mauritius or Mayotte or Mexico or Micronesia or Montenegro or Nevis or “Northern Mariana Islands” or Oman or Palau or Panama or Poland or Romania or Russia or “Russian Federation” or Samoa or “Saint Lucia” or “St Lucia” or “Saint Kitts” or “St Kitts” or “Saint Vincent” or “St Vincent” or Serbia or Seychelles or Slovakia or “Slovak Republic” or “South Africa” or Turkey or Uruguay or Venezuela or Yugoslavia) TI (“American Samoa” or Argentina or Belize or Botswana or Brazil or Bulgaria or Chile or Comoros or “Costa Rica” or Croatia or Dominica or Guinea or Gabon or Grenada or Grenadines or Hungary or Kazakhstan or Latvia or Lebanon or Libia or libyan or Libya or Lithuania or Malaysia or Mauritius or Mayotte or Mexico or Micronesia or Montenegro or Nevis or “Northern Mariana Islands” or Oman or Palau or Panama or Poland or Romania or Russia or “Russian Federation” or Samoa or “Saint Lucia” or “St Lucia” or “Saint Kitts” or “St Kitts” or “Saint Vincent” or “St Vincent” or Serbia or Seychelles or Slovakia or “Slovak Republic” or “South Africa” or Turkey or Uruguay or Venezuela or Yugoslavia) AB (“American Samoa” or Argentina or Belize or Botswana or Brazil or Bulgaria or Chile or Comoros or “Costa Rica” or Croatia or Dominica or Guinea or Gabon or Grenada or Grenadines or Hungary or Kazakhstan or Latvia or Lebanon or Libia or libyan or Libya or Lithuania or Malaysia or Mauritius or Mayotte or Mexico or Micronesia or Montenegro or Nevis or “Northern Mariana Islands” or Oman or Palau or Panama or Poland or Romania or Russia or “Russian Federation” or Samoa or “Saint Lucia” or “St Lucia” or “Saint Kitts” or “St Kitts” or “Saint Vincent” or “St Vincent” or Serbia or Seychelles or Slovakia or “Slovak Republic” or “South Africa” or Turkey or Uruguay or Venezuela or Yugoslavia)
	TI (Africa or Asia or “South America” or “Latin America” or “Central America”)
	AB (Africa or Asia or “South America” or “Latin America” or “Central America”)
	(SU “Asia+”)
	(SU “West Indies+”)
	(SU “South America+”)
	(SU “Latin America”)
	(SU “Central America+”)
	(SU “Africa+”)
	(SU “Developing Countries”)
	**or/**
	(SU "Juvenile Delinquency")
	AB (juvenile N1 delinquen*)
	AB (school N1 violence)
	(SU "Juvenile Offenders+")
	(SU "Child Behavior Disorders")
	**or/**
	SU ("Crime")
	SU ("Aggression")
	SU ("Bullying")
	SU ("Violence")
	(SU "Violence")
	(SU "Homicide")
	AB (conduct N1 problem*)
	AB (behavio#r N1 problem*)
	AB (disruptive N1 behavio#r)
	AB (conduct N1 disorder*)
	AB (behavio#r N1 disorder*)
	AB (aggressive N1 behavio#r)
	AB (aggression)
	AB (aggressive)
	AB (antisocial N1 behavio#r)
	AB (anti‐social N1 behavio#r)
	AB (gang)
	AB (gangs)
	AB (criminal N1 behavio#r)
	AB (violent N1 crime)
	AB (homicid*)
	AB (violence)
	AB (violent)
	AB (crime)
	AB (crimes)
	AB (criminal*)
	AB (bully)
	AB (bullying)
	AB (delinquent*)
	AB (delinquenc*)
	TX (oppositional N1 defiant N1 disorder*)
	TX (disruptive N1 behavio#r N1 disorder*)
	AB (externalizing N1 behavio#r N1 problem*)
	AB (externalizing)
	AB (externalising)
	AB (externalized)
	AB (externalised)
	AB (externaliz*)
	AB (externalis*)
	AB (externalizing N1 behavio#r)
	AB (externalising N1 behavio#r)
	or /…
	S21 AND S68
	(SU " Child+")
	(SU "Adolescence")
	AB (Adolescen*)
	AB (Adolescence)
	AB (Adolescent)
	AB (adolescents)
	AB (Child*)
	AB (child)
	AB (children)
	AB (childhood)
	AB (youth*)
	AB (youth)
	AB (youths)
	AB (student*)
	AB (Students)
	AB (Student)
	AB (teen*)
	AB (teenager)
	AB (teenagers)
	AB (boy*)
	AB (boy)
	AB (boys)
	AB (girl*)
	AB (girl)
	AB (girls)
	AB (pupil)
	AB (pupils)
	AB (pupil*)
	AB (youngster*)
	AB (youngster)
	AB (youngsters)
	AB (juvenile*)
	AB (juvenile)
	AB (juveniles)
	AB (young N1 adult*)
	AB (infant*)
	AB (infants)
	AB (infant)
	AB (baby*)
	AB (baby)
	AB (babies)
	AB (toddler)
	AB (toddler*)
	AB (toddlers)
	or/
	22 and 38
**Sociological Abstracts+Social Services Abstracts (ProQuest)**	ab(Africa or Asia or "Latin America" or "South America" or Caribbean or "West Indies" or "Eastern Europe" or Soviet or Arab or "Middle East" or "Latin America" or "Central America") OR (ab(Afghanistan or Albania or Algeria or Angola or Antigua or Barbuda or Argentina or Armenia or Armenian or Aruba or Azerbaijan or Bahrain or Bangladesh or Barbados or Benin or Byelarus or Byelorussian or Belarus or Belorussian or Belorussia or Belize or Bhutan or Bolivia or Bosnia or Herzegovina or Hercegovina or Botswana or Brasil or Brazil or Bulgaria or Burkina Faso or Burkina Fasso or Upper Volta or Burundi or Urundi or Cambodia or Khmer Republic or Kampuchea or Cameroon or Cameroons or Cameron or Camerons or Cape Verde or Central African Republic or Chad or Chile or China or Colombia or Comoros or Comoro Islands or Comores or Mayotte or Congo or Zaire or Costa Rica or Cote d'Ivoire or Ivory Coast or Croatia or Cuba or Cyprus or Czechoslovakia or Czech Republic or Slovakia or Slovak Republic or Djibouti or French Somaliland or Dominica or Dominican Republic or East Timor or East Timur or Timor Leste or Ecuador or Egypt or United Arab Republic or El Salvador or Eritrea or Estonia or Ethiopia or Fiji or Gabon or Gabonese Republic or Gambia or Gaza or Georgia Republic or Georgian Republic or Ghana or Gold Coast or Greece or Grenada or Guatemala or Guinea or Guam or Guiana or Guyana or Haiti or Honduras or Hungary or India or Maldives or Indonesia or Iran or Iraq or Isle of Man or Jamaica or Jordan or Kazakhstan or Kazakh or Kenya or Kiribati or Korea or Kosovo or Kyrgyzstan or Kirghizia or Kyrgyz Republic or Kirghiz or Kirgizstan or Lao PDR or Laos or Latvia or Lebanon or Lesotho or Basutoland or Liberia or Libya or Lithuania or Macedonia or Madagascar or Malagasy Republic or Malaysia or Malaya or Malay or Sabah or Sarawak or Malawi or Nyasaland or Mali or Malta or Marshall Islands or Mauritania or Mauritius or Agalega Islands or Mexico or Micronesia or Middle East or Moldova or Moldovia or Moldovian or Mongolia or Montenegro or Morocco or Ifni or Mozambique or Myanmar or Myanma or Burma or Namibia or Nepal or Netherlands Antilles or New Caledonia or Nicaragua or Niger or Nigeria or Northern Mariana Islands or Oman or Muscat or Pakistan or Palau or Palestine or Panama or Paraguay or Peru or Philippines or Philipines or Phillipines or Phillippines or Poland or Portugal or Puerto Rico or Romania or Rumania or Roumania or Russia or Russian or Rwanda or Ruanda or Saint Kitts or St Kitts or Nevis or Saint Lucia or St Lucia or Saint Vincent or St Vincent or Grenadines or Samoa or Samoan Islands or Navigator Island or Navigator Islands or Sao Tome or Saudi Arabia or Senegal or Serbia or Montenegro or Seychelles or Sierra Leone or Slovenia or Sri Lanka or Ceylon or Solomon Islands or Somalia or South Africa or Sudan or Suriname or Surinam or Swaziland or Syria or Tajikistan or Tadzhikistan or Tadjikistan or Tadzhik or Tanzania or Thailand or Togo or Togolese Republic or Tonga or Trinidad or Tobago or Tunisia or Turkey or Turkmenistan or Turkmen or Uganda or Ukraine or Uruguay or USSR or Soviet Union or Union of Soviet Socialist Republics or Uzbekistan or Uzbek or Vanuatu or New Hebrides or Venezuela or Vietnam or Viet Nam or West Bank or Yemen or Yugoslavia or Zambia or Zimbabwe or Rhodesia)) OR (AB “Developing Countries”) OR (ab(developing NEAR/1 world)) OR (ab(poor* NEAR/1 nation*)) OR (ab(developing NEAR/1 countr*)) OR (ab(developing NEAR/1 region*)) OR (ab(third NEAR/1 world)) OR SU.EXACT.EXPLODE("Developing Countries")
	(SU.EXACT.EXPLODE("Crime")) OR (SU.EXACT.EXPLODE("Aggression")) OR (SU.EXACT.EXPLODE("Behavior Problems")) OR (SU.EXACT.EXPLODE("Violence")) OR (SU.EXACT.EXPLODE("Gangs")) OR (ab(gang*)) OR (ab(conduct NEAR/1 problem*)) OR (ab(behavio*r NEAR/1 problem*)) OR (ab(conduct NEAR/1 disorder*)) OR (ab(antisocial NEAR/1 behavio*r*)) OR (ab(oppositional NEAR/1 defiant NEAR/1 disorder*)) OR (AB "Aggression") OR (AB "Social Behavior Disorders") OR (AB "Crime") OR (AB "Violence") OR (AB "Homicide") OR (AB "Assault and Battery") OR (AB "Aggression") OR (AB(conduct NEAR/1 problem*)) OR (AB(behavio#r NEAR/1 problem*)) OR (AB(disruptive NEAR/1 behavio#r)) OR (AB(conduct NEAR/1 disorder*)) OR (AB(behavio#r NEAR/1 disorder*)) OR (AB(aggressive NEAR/1 behavio#r)) OR (AB(aggression) OR AB(aggressive)) OR (AB(antisocial NEAR/1 behavio#r)) OR (AB(anti‐social NEAR/1 behavio#r)) OR (AB(gang)) OR (AB(gangs)) OR (AB(criminal N1 behavio#r)) OR (AB(violent NEAR/1 crime)) OR (AB(homicid*)) OR (AB(violence)) OR (AB(violent)) OR (AB(crime)) OR (AB(crimes)) OR (AB(criminal*)) OR (AB(bully)) OR (AB(bullying)) OR TX (oppositional N1 defiant N1 disorder*) OR TX (disruptive N1 behavio#r N1 disorder*
	AB(delinquent*) OR AB(delinquenc*) OR AB(school NEAR/1 violence) OR AB(juvenile NEAR/1 delinquency) OR AB(juvenile NEAR/1 delinquent) OR AB(juvenile NEAR/1 delinquents) OR SU.exact("JUVENILE DELINQUENCY") OR SU.exact("DELINQUENCY") OR SU.exact("JUVENILE OFFENDERS")
	(SU.EXACT.EXPLODE("Adolescents")) OR (SU.EXACT.EXPLODE("Infants")) OR (SU.EXACT.EXPLODE("Children")) OR (AB "Adolescence") OR AB(Adolescen*) OR AB(Adolescence) OR AB(Adolescent) OR AB(adolescents) OR AB(Child*) OR AB(child) OR AB(children) OR AB(childhood) OR AB(youth*) OR AB(youth) OR AB(youths) OR AB(student*) OR AB(Students) OR AB(Student) OR AB(teen*) OR AB(teenager) OR AB(teenagers) OR AB(boy*) OR AB(boy) OR AB(boys) OR AB(girl*) OR AB(girl) OR AB(girls) OR AB(pupil) OR AB(pupils) OR AB(pupil*) OR AB(youngster*) OR AB(youngster) OR AB(youngsters) OR AB(juvenile*) OR AB(juvenile) OR AB(juveniles) OR AB(young NEAR/1 adult*) OR AB(infant*) OR AB(infants) OR AB(infant) OR AB(baby*) OR AB(baby) OR AB(babies) OR AB(toddler) OR AB(toddler*) OR AB(toddlers)
**Applied Social Sciences Index and Abstracts (ProQuest)**	(ab(Africa or Asia or "Latin America" or "South America" or Caribbean or "West Indies" or "Eastern Europe" or Soviet or Arab or "Middle East" or "Latin America" or "Central America")) OR (ab(Afghanistan or Albania or Algeria or Angola or Antigua or Barbuda or Argentina or Armenia or Armenian or Aruba or Azerbaijan or Bahrain or Bangladesh or Barbados or Benin or Byelarus or Byelorussian or Belarus or Belorussian or Belorussia or Belize or Bhutan or Bolivia or Bosnia or Herzegovina or Hercegovina or Botswana or Brasil or Brazil or Bulgaria or Burkina Faso or Burkina Fasso or Upper Volta or Burundi or Urundi or Cambodia or Khmer Republic or Kampuchea or Cameroon or Cameroons or Cameron or Camerons or Cape Verde or Central African Republic or Chad or Chile or China or Colombia or Comoros or Comoro Islands or Comores or Mayotte or Congo or Zaire or Costa Rica or Cote d'Ivoire or Ivory Coast or Croatia or Cuba or Cyprus or Czechoslovakia or Czech Republic or Slovakia or Slovak Republic or Djibouti or French Somaliland or Dominica or Dominican Republic or East Timor or East Timur or Timor Leste or Ecuador or Egypt or United Arab Republic or El Salvador or Eritrea or Estonia or Ethiopia or Fiji or Gabon or Gabonese Republic or Gambia or Gaza or Georgia Republic or Georgian Republic or Ghana or Gold Coast or Greece or Grenada or Guatemala or Guinea or Guam or Guiana or Guyana or Haiti or Honduras or Hungary or India or Maldives or Indonesia or Iran or Iraq or Isle of Man or Jamaica or Jordan or Kazakhstan or Kazakh or Kenya or Kiribati or Korea or Kosovo or Kyrgyzstan or Kirghizia or Kyrgyz Republic or Kirghiz or Kirgizstan or Lao PDR or Laos or Latvia or Lebanon or Lesotho or Basutoland or Liberia or Libya or Lithuania or Macedonia or Madagascar or Malagasy Republic or Malaysia or Malaya or Malay or Sabah or Sarawak or Malawi or Nyasaland or Mali or Malta or Marshall Islands or Mauritania or Mauritius or Agalega Islands or Mexico or Micronesia or Middle East or Moldova or Moldovia or Moldovian or Mongolia or Montenegro or Morocco or Ifni or Mozambique or Myanmar or Myanma or Burma or Namibia or Nepal or Netherlands Antilles or New Caledonia or Nicaragua or Niger or Nigeria or Northern Mariana Islands or Oman or Muscat or Pakistan or Palau or Palestine or Panama or Paraguay or Peru or Philippines or Philipines or Phillipines or Phillippines or Poland or Portugal or Puerto Rico or Romania or Rumania or Roumania or Russia or Russian or Rwanda or Ruanda or Saint Kitts or St Kitts or Nevis or Saint Lucia or St Lucia or Saint Vincent or St Vincent or Grenadines or Samoa or Samoan Islands or Navigator Island or Navigator Islands or Sao Tome or Saudi Arabia or Senegal or Serbia or Montenegro or Seychelles or Sierra Leone or Slovenia or Sri Lanka or Ceylon or Solomon Islands or Somalia or South Africa or Sudan or Suriname or Surinam or Swaziland or Syria or Tajikistan or Tadzhikistan or Tadjikistan or Tadzhik or Tanzania or Thailand or Togo or Togolese Republic or Tonga or Trinidad or Tobago or Tunisia or Turkey or Turkmenistan or Turkmen or Uganda or Ukraine or Uruguay or USSR or Soviet Union or Union of Soviet Socialist Republics or Uzbekistan or Uzbek or Vanuatu or New Hebrides or Venezuela or Vietnam or Viet Nam or West Bank or Yemen or Yugoslavia or Zambia or Zimbabwe or Rhodesia)) OR (AB “Developing Countries”) OR (ab(developing NEAR/1 world)) OR (ab(poor* NEAR/1 nation*)) OR (ab(developing NEAR/1 countr*)) OR (ab(developing NEAR/1 region*)) OR (ab(third NEAR/1 world)) OR (SU.EXACT.EXPLODE"Developing Countries")
	(SU.EXACT.EXPLODE("Crime")) OR (SU.EXACT.EXPLODE("Aggression")) OR (SU.EXACT("Bullying")) OR (SU.EXACT.EXPLODE("Violence")) OR (SU.EXACT ("Criminal behaviour")) OR (SU.EXACT ("Oppositional defiant disorder")) OR SU.exact("CONDUCT DISORDERS") OR (ab(gang*)) OR (ab(conduct NEAR/1 problem*)) OR (ab(behavio*r NEAR/1 problem*)) OR (ab(conduct NEAR/1 disorder*)) OR (ab(antisocial NEAR/1 behavio*r*)) OR (ab(oppositional NEAR/1 defiant NEAR/1 disorder*)) OR (AB "Aggression") OR (AB "Social Behavior Disorders") OR (AB "Crime") OR (AB "Violence") OR (AB "Homicide") OR (AB "Assault and Battery") OR (AB "Aggression") OR (AB(conduct NEAR/1 problem*)) OR (AB(behavio#r NEAR/1 problem*)) OR (AB(disruptive NEAR/1 behavio#r)) OR (AB(conduct NEAR/1 disorder*)) OR (AB(behavio#r NEAR/1 disorder*)) OR (AB(aggressive NEAR/1 behavio#r)) OR (AB(aggression) OR AB(aggressive)) OR (AB(antisocial NEAR/1 behavio#r)) OR (AB(anti‐social NEAR/1 behavio#r)) OR (AB(gang)) OR (AB(gangs)) OR (AB(criminal N1 behavio#r)) OR (AB(violent NEAR/1 crime)) OR (AB(homicid*)) OR (AB(violence)) OR (AB(violent)) OR (AB(crime)) OR (AB(crimes)) OR (AB(criminal*)) OR (AB(bully)) OR (AB(bullying)) OR TX (oppositional N1 defiant N1 disorder*) OR TX (disruptive N1 behavio#r N1 disorder*) AB(delinquent*) OR AB(delinquenc*) OR AB(school NEAR/1 violence) OR AB(juvenile NEAR/1 delinquency) OR AB(juvenile NEAR/1 delinquent) OR AB(juvenile NEAR/1 delinquents) OR SU.exact("JUVENILE DELINQUENCY") OR SU.exact("DELINQUENCY") OR SU.exact("JUVENILE OFFENDERS")
	(SU.EXACT.EXPLODE"Children") OR (SU.EXACT.EXPLODE("Adolescence")) OR (SU.EXACT.EXPLODE("Youth")) OR (AB "Adolescence") OR AB(Adolescen*) OR AB(Adolescence) OR AB(Adolescent) OR AB(adolescents) OR AB(Child*) OR AB(child) OR AB(children) OR AB(childhood) OR AB(youth*) OR AB(youth) OR AB(youths) OR AB(student*) OR AB(Students) OR AB(Student) OR AB(teen*) OR AB(teenager) OR AB(teenagers) OR AB(boy*) OR AB(boy) OR AB(boys) OR AB(girl*) OR AB(girl) OR AB(girls) OR AB(pupil) OR AB(pupils) OR AB(pupil*) OR AB(youngster*) OR AB(youngster) OR AB(youngsters) OR AB(juvenile*) OR AB(juvenile) OR AB(juveniles) OR AB(young NEAR/1 adult*) OR AB(infant*) OR AB(infants) OR AB(infant) OR AB(baby*) OR AB(baby) OR AB(babies) OR AB(toddler) OR AB(toddler*) OR AB(toddlers)
**International Bibliography of the Social Sciences (IBSS) (ProQuest)**	(ab(Africa or Asia or "Latin America" or "South America" or Caribbean or "West Indies" or "Eastern Europe" or Soviet or Arab or "Middle East" or "Latin America" or "Central America")) OR (ab(Afghanistan or Albania or Algeria or Angola or Antigua or Barbuda or Argentina or Armenia or Armenian or Aruba or Azerbaijan or Bahrain or Bangladesh or Barbados or Benin or Byelarus or Byelorussian or Belarus or Belorussian or Belorussia or Belize or Bhutan or Bolivia or Bosnia or Herzegovina or Hercegovina or Botswana or Brasil or Brazil or Bulgaria or Burkina Faso or Burkina Fasso or Upper Volta or Burundi or Urundi or Cambodia or Khmer Republic or Kampuchea or Cameroon or Cameroons or Cameron or Camerons or Cape Verde or Central African Republic or Chad or Chile or China or Colombia or Comoros or Comoro Islands or Comores or Mayotte or Congo or Zaire or Costa Rica or Cote d'Ivoire or Ivory Coast or Croatia or Cuba or Cyprus or Czechoslovakia or Czech Republic or Slovakia or Slovak Republic or Djibouti or French Somaliland or Dominica or Dominican Republic or East Timor or East Timur or Timor Leste or Ecuador or Egypt or United Arab Republic or El Salvador or Eritrea or Estonia or Ethiopia or Fiji or Gabon or Gabonese Republic or Gambia or Gaza or Georgia Republic or Georgian Republic or Ghana or Gold Coast or Greece or Grenada or Guatemala or Guinea or Guam or Guiana or Guyana or Haiti or Honduras or Hungary or India or Maldives or Indonesia or Iran or Iraq or Isle of Man or Jamaica or Jordan or Kazakhstan or Kazakh or Kenya or Kiribati or Korea or Kosovo or Kyrgyzstan or Kirghizia or Kyrgyz Republic or Kirghiz or Kirgizstan or Lao PDR or Laos or Latvia or Lebanon or Lesotho or Basutoland or Liberia or Libya or Lithuania or Macedonia or Madagascar or Malagasy Republic or Malaysia or Malaya or Malay or Sabah or Sarawak or Malawi or Nyasaland or Mali or Malta or Marshall Islands or Mauritania or Mauritius or Agalega Islands or Mexico or Micronesia or Middle East or Moldova or Moldovia or Moldovian or Mongolia or Montenegro or Morocco or Ifni or Mozambique or Myanmar or Myanma or Burma or Namibia or Nepal or Netherlands Antilles or New Caledonia or Nicaragua or Niger or Nigeria or Northern Mariana Islands or Oman or Muscat or Pakistan or Palau or Palestine or Panama or Paraguay or Peru or Philippines or Philipines or Phillipines or Phillippines or Poland or Portugal or Puerto Rico or Romania or Rumania or Roumania or Russia or Russian or Rwanda or Ruanda or Saint Kitts or St Kitts or Nevis or Saint Lucia or St Lucia or Saint Vincent or St Vincent or Grenadines or Samoa or Samoan Islands or Navigator Island or Navigator Islands or Sao Tome or Saudi Arabia or Senegal or Serbia or Montenegro or Seychelles or Sierra Leone or Slovenia or Sri Lanka or Ceylon or Solomon Islands or Somalia or South Africa or Sudan or Suriname or Surinam or Swaziland or Syria or Tajikistan or Tadzhikistan or Tadjikistan or Tadzhik or Tanzania or Thailand or Togo or Togolese Republic or Tonga or Trinidad or Tobago or Tunisia or Turkey or Turkmenistan or Turkmen or Uganda or Ukraine or Uruguay or USSR or Soviet Union or Union of Soviet Socialist Republics or Uzbekistan or Uzbek or Vanuatu or New Hebrides or Venezuela or Vietnam or Viet Nam or West Bank or Yemen or Yugoslavia or Zambia or Zimbabwe or Rhodesia)) OR (AB “Developing Countries”) OR (ab(developing NEAR/1 world)) OR (ab(poor* NEAR/1 nation*)) OR (ab(developing NEAR/1 countr*)) OR (ab(developing NEAR/1 region*)) OR (ab(third NEAR/1 world)) OR (SU.EXACT.EXPLODE("Developing Countries"))
	(SU.EXACT.EXPLODE("Crime")) OR (SU.EXACT.EXPLODE("Aggression")) OR (SU.EXACT.EXPLODE("Bullying")) OR (SU.EXACT ("Violence")) OR (SU.EXACT.EXPLODE("Gang")) OR (SU.EXACT.EXPLODE("Crime")) OR (SU.EXACT.EXPLODE("Aggression")) OR (SU.EXACT.EXPLODE("Bullying")) OR (SU.EXACT.EXPLODE("Violence")) OR (ab(gang*)) OR (ab(conduct NEAR/1 problem*)) OR (ab(behavio*r NEAR/1 problem*)) OR (ab(conduct NEAR/1 disorder*)) OR (ab(antisocial NEAR/1 behavio*r*)) OR (ab(oppositional NEAR/1 defiant NEAR/1 disorder*)) OR (AB "Aggression") OR (AB "Social Behavior Disorders") OR (AB "Crime") OR (AB "Violence") OR (AB "Homicide") OR (AB "Assault and Battery") OR (AB "Aggression") OR (AB(conduct NEAR/1 problem*)) OR (AB(behavio#r NEAR/1 problem*)) OR (AB(disruptive NEAR/1 behavio#r)) OR (AB(conduct NEAR/1 disorder*)) OR (AB(behavio#r NEAR/1 disorder*)) OR (AB(aggressive NEAR/1 behavio#r)) OR (AB(aggression) OR AB(aggressive)) OR (AB(antisocial NEAR/1 behavio#r)) OR (AB(anti‐social NEAR/1 behavio#r)) OR (AB(gang)) OR (AB(gangs)) OR (AB(criminal N1 behavio#r)) OR (AB(violent NEAR/1 crime)) OR (AB(homicid*)) OR (AB(violence)) OR (AB(violent)) OR (AB(crime)) OR (AB(crimes)) OR (AB(criminal*)) OR (AB(bully)) OR (AB(bullying))
	AB(delinquent*) OR AB(delinquenc*) OR TX (oppositional N1 defiant N1 disorder*) OR TX (disruptive N1 behavio#r N1 disorder*) OR AB(school NEAR/1 violence) OR AB(juvenile NEAR/1 delinquency) OR AB(juvenile NEAR/1 delinquent) OR AB(juvenile NEAR/1 delinquents)
	(SU.EXACT.EXPLODE("Children")) OR (SU.EXACT.EXPLODE("Adolescence")) OR (SU.EXACT.EXPLODE("Youth")) OR (AB "Adolescence") OR AB(Adolescen*) OR AB(Adolescence) OR AB(Adolescent) OR AB(adolescents) OR AB(Child*) OR AB(child) OR AB(children) OR AB(childhood) OR AB(youth*) OR AB(youth) OR AB(youths) OR AB(student*) OR AB(Students) OR AB(Student) OR AB(teen*) OR AB(teenager) OR AB(teenagers) OR AB(boy*) OR AB(boy) OR AB(boys) OR AB(girl*) OR AB(girl) OR AB(girls) OR AB(pupil) OR AB(pupils) OR AB(pupil*) OR AB(youngster*) OR AB(youngster) OR AB(youngsters) OR AB(juvenile*) OR AB(juvenile) OR AB(juveniles) OR AB(young NEAR/1 adult*) OR AB(infant*) OR AB(infants) OR AB(infant) OR AB(baby*) OR AB(baby) OR AB(babies) OR AB(toddler) OR AB(toddler*) OR AB(toddlers)
**ERIC (ProQuest)**	(ab(Africa or Asia or "Latin America" or "South America" or Caribbean or "West Indies" or "Eastern Europe" or Soviet or Arab or "Middle East" or "Latin America" or "Central America")) OR (ab(Afghanistan or Albania or Algeria or Angola or Antigua or Barbuda or Argentina or Armenia or Armenian or Aruba or Azerbaijan or Bahrain or Bangladesh or Barbados or Benin or Byelarus or Byelorussian or Belarus or Belorussian or Belorussia or Belize or Bhutan or Bolivia or Bosnia or Herzegovina or Hercegovina or Botswana or Brasil or Brazil or Bulgaria or Burkina Faso or Burkina Fasso or Upper Volta or Burundi or Urundi or Cambodia or Khmer Republic or Kampuchea or Cameroon or Cameroons or Cameron or Camerons or Cape Verde or Central African Republic or Chad or Chile or China or Colombia or Comoros or Comoro Islands or Comores or Mayotte or Congo or Zaire or Costa Rica or Cote d'Ivoire or Ivory Coast or Croatia or Cuba or Cyprus or Czechoslovakia or Czech Republic or Slovakia or Slovak Republic or Djibouti or French Somaliland or Dominica or Dominican Republic or East Timor or East Timur or Timor Leste or Ecuador or Egypt or United Arab Republic or El Salvador or Eritrea or Estonia or Ethiopia or Fiji or Gabon or Gabonese Republic or Gambia or Gaza or Georgia Republic or Georgian Republic or Ghana or Gold Coast or Greece or Grenada or Guatemala or Guinea or Guam or Guiana or Guyana or Haiti or Honduras or Hungary or India or Maldives or Indonesia or Iran or Iraq or Isle of Man or Jamaica or Jordan or Kazakhstan or Kazakh or Kenya or Kiribati or Korea or Kosovo or Kyrgyzstan or Kirghizia or Kyrgyz Republic or Kirghiz or Kirgizstan or Lao PDR or Laos or Latvia or Lebanon or Lesotho or Basutoland or Liberia or Libya or Lithuania or Macedonia or Madagascar or Malagasy Republic or Malaysia or Malaya or Malay or Sabah or Sarawak or Malawi or Nyasaland or Mali or Malta or Marshall Islands or Mauritania or Mauritius or Agalega Islands or Mexico or Micronesia or Middle East or Moldova or Moldovia or Moldovian or Mongolia or Montenegro or Morocco or Ifni or Mozambique or Myanmar or Myanma or Burma or Namibia or Nepal or Netherlands Antilles or New Caledonia or Nicaragua or Niger or Nigeria or Northern Mariana Islands or Oman or Muscat or Pakistan or Palau or Palestine or Panama or Paraguay or Peru or Philippines or Philipines or Phillipines or Phillippines or Poland or Portugal or Puerto Rico or Romania or Rumania or Roumania or Russia or Russian or Rwanda or Ruanda or Saint Kitts or St Kitts or Nevis or Saint Lucia or St Lucia or Saint Vincent or St Vincent or Grenadines or Samoa or Samoan Islands or Navigator Island or Navigator Islands or Sao Tome or Saudi Arabia or Senegal or Serbia or Montenegro or Seychelles or Sierra Leone or Slovenia or Sri Lanka or Ceylon or Solomon Islands or Somalia or South Africa or Sudan or Suriname or Surinam or Swaziland or Syria or Tajikistan or Tadzhikistan or Tadjikistan or Tadzhik or Tanzania or Thailand or Togo or Togolese Republic or Tonga or Trinidad or Tobago or Tunisia or Turkey or Turkmenistan or Turkmen or Uganda or Ukraine or Uruguay or USSR or Soviet Union or Union of Soviet Socialist Republics or Uzbekistan or Uzbek or Vanuatu or New Hebrides or Venezuela or Vietnam or Viet Nam or West Bank or Yemen or Yugoslavia or Zambia or Zimbabwe or Rhodesia)) OR (AB “Developing Countries”) OR (SU.EXACT.EXPLODE("Developing Countries")) OR (ab(developing NEAR/1 world)) OR (ab(poor* NEAR/1 nation*)) OR (ab(developing NEAR/1 countr*)) OR (ab(developing NEAR/1 region*)) OR (ab(third NEAR/1 world)**) OR** (SU.EXACT.EXPLODE("Foreign Countries")) OR (SU.EXACT.EXPLODE("Developing Nations"))
	(SU.EXACT.EXPLODE("Crime")) OR (SU.EXACT.EXPLODE("Aggression")) OR (SU.EXACT.EXPLODE("Bullying")) OR (SU.EXACT.EXPLODE("Violence")) OR (ab(gang*)) OR (ab(conduct NEAR/1 problem*)) OR (ab(behavio*r NEAR/1 problem*)) OR (ab(conduct NEAR/1 disorder*)) OR (ab(antisocial NEAR/1 behavio*r*)) OR (ab(oppositional NEAR/1 defiant NEAR/1 disorder*)) OR (AB "Aggression") OR (AB "Social Behavior Disorders") OR (AB "Crime") OR (AB "Violence") OR (AB "Homicide") OR (AB "Assault and Battery") OR (AB "Aggression") OR (AB(conduct NEAR/1 problem*)) OR (AB(behavio#r NEAR/1 problem*)) OR (AB(disruptive NEAR/1 behavio#r)) OR (AB(conduct NEAR/1 disorder*)) OR (AB(behavio#r NEAR/1 disorder*)) OR (AB(aggressive NEAR/1 behavio#r)) OR (AB(aggression) OR AB(aggressive)) OR (AB(antisocial NEAR/1 behavio#r)) OR (AB(anti‐social NEAR/1 behavio#r)) OR (AB(gang)) OR (AB(gangs)) OR (AB(criminal N1 behavio#r)) OR (AB(violent NEAR/1 crime)) OR (AB(homicid*)) OR (AB(violence)) OR (AB(violent)) OR (AB(crime)) OR (AB(crimes)) OR (AB(criminal*)) OR (AB(bully)) OR (AB(bullying)) AB(delinquent*) OR AB(delinquenc*) OR TX (oppositional N1 defiant N1 disorder*) OR TX (disruptive N1 behavio#r N1 disorder*) OR AB(school NEAR/1 violence) OR AB(juvenile NEAR/1 delinquency) OR AB(juvenile NEAR/1 delinquent) OR AB(juvenile NEAR/1 delinquents) (SU.EXACT.EXPLODE("Adolescents")) OR (SU.EXACT.EXPLODE("Early Adolescents")) OR (SU.EXACT.EXPLODE("Children")) OR (SU.EXACT.EXPLODE("Youth")) OR (SU.EXACT.EXPLODE("Late Adolescents")) OR (AB "Adolescence") OR AB(Adolescen*) OR AB(Adolescence) OR AB(Adolescent) OR AB(adolescents) OR AB(Child*) OR AB(child) OR AB(children) OR AB(childhood) OR AB(youth*) OR AB(youth) OR AB(youths) OR AB(student*) OR AB(Students) OR AB(Student) OR AB(teen*) OR AB(teenager) OR AB(teenagers) OR AB(boy*) OR AB(boy) OR AB(boys) OR AB(girl*) OR AB(girl) OR AB(girls) OR AB(pupil) OR AB(pupils) OR AB(pupil*) OR AB(youngster*) OR AB(youngster) OR AB(youngsters) OR AB(juvenile*) OR AB(juvenile) OR AB(juveniles) OR AB(young NEAR/1 adult*) OR AB(infant*) OR AB(infants) OR AB(infant) OR AB(baby*) OR AB(baby) OR AB(babies) OR AB(toddler) OR AB(toddler*) OR AB(toddlers)
National Criminal Justice Reference Service Abstracts Database	“Developing Countries”
**Web of Science**	Topic=(infants)
	Topic=(infant)
	Topic=(Infant*)
	Topic=(juveniles)
	Topic=(juvenile)
	Topic=(juvenile*)
	Topic=(youngsters)
	Topic=(youngster)
	Topic=(youngster*)
	Topic=(pupil*)
	Topic=(pupils)
	Topic=(pupil)
	Topic=(girls)
	Topic=(girl)
	Topic=(boys)
	Topic=(boy)
	Topic=(teenagers)
	Topic=(teenager)
	Topic=(teen*)
	Topic=(students)
	Topic=(student)
	Topic=(student*)
	Topic=(youths)
	Topic=(youth)
	Topic=(youth*)
	Topic=(childhood)
	Topic=(childhood*)
	Topic=(children*)
	Topic=(child)
	Topic=(child*)
	Topic=(adolescence)
	Topic=(adolescents)
	Topic=(adolescent)
	Topic=(adolescen*)
	Topic=(toddlers)
	Topic=(toddler)
	Topic=(babies)
	Topic=(baby)
	Topic=(young NEAR/1 adult*)
	Topic=(externalis*)
	Topic=(externaliz*)
	Topic=(externalised)
	Topic=(externalized)
	Topic=(externalising)
	Topic=(externalizing)
	Topic=(bully)
	Topic=(bullying)
	Topic=(bully*)
	Topic=(criminal NEAR/1 behavio$r*)
	Topic=(criminal*)
	Topic=(crimes)
	Topic=(crime)
	Topic=(violent NEAR/1 crime*)
	Topic=(aggressive NEAR/1 behavio$r*)
	Topic=(anti‐social)
	Topic=(antisocial)
	Topic=(aggressive)
	Topic=(aggress*)
	Topic=(aggression)
	Topic=(antisocial NEAR/1 behavio$r*)
	Topic=(disruptive NEAR/1 behavio$r NEAR/1 disorder*)
	Topic=(oppositional NEAR/1 defiant NEAR/1 disorder*)
	Topic=(behavio$r NEAR/1 disorder*)
	Topic=(behavio$r NEAR/1 problem*)
	Topic=(conduct NEAR/1 disorder*)
	Topic=(conduct NEAR/1 problem*)
	Topic=(gangs)
	Topic=(gang)
	Topic=(homicide*)
	Topic=(violen*)
	Topic=(violence)
	Topic=(violent)
	Topic=(school NEAR/1 violence)
	Topic=(juvenile NEAR/1 delinquent)
	Topic=(juvenile NEAR/1 delinquency)
	Topic=(deprived NEAR/1 (countr* OR nation*))
	Topic=((“less developed”) NEAR/1 (countr* OR nation*))
	Topic=((“under developed”) NEAR/1 (countr* OR nation*))
	Topic=((“low income”) NEAR/1 (economy or economies))
	Topic=((“under developed”) NEAR/1 (economy or economies))
	Topic=((“middle income”) NEAR/1 (economy or economies))
	Topic=((“under developed”) NEAR/1 (economy or economies))
	Topic=(“less developed” NEAR/1 (economy or economies))
	Topic=((“under developed”) NEAR/1 (economy or economies))
	Topic=(underdeveloped NEAR/1 (economy or economies))
	Topic=((poor) NEAR/1 (countr* OR nation*))
	Topic=((developing NEAR/1 nation*))
	Topic=((developing NEAR/1 region*))
	Topic=((developing NEAR/1 countr*))
	Topic=((developing NEAR/1 world))
	Topic=((developing) NEAR/1 (economy or economies))
	Topic=(third NEAR/1 world)
	Topic=(Afghanistan or Albania or Algeria or Angola or Antigua or Barbuda or Argentina or Armenia or Armenian or Aruba or Azerbaijan or Bahrain or Bangladesh or Barbados or Benin or Byelarus or Byelorussian or Belarus or Belorussian or Belorussia or Belize or Bhutan or Bolivia or Bosnia or Herzegovina or Hercegovina or Botswana or Brasil or Brazil or Bulgaria or Burkina Faso or Burkina Fasso or Upper Volta or Burundi or Urundi or Cambodia or Khmer Republic or Kampuchea or Cameroon or Cameroons or Cameron or Camerons or Cape Verde or Central African Republic or Chad or Chile or China or Colombia or Comoros or Comoro Islands or Comores or Mayotte or Congo or Zaire or Costa Rica or Cote d'Ivoire or Ivory Coast or Croatia or Cuba or Cyprus or Czechoslovakia or Czech Republic or Slovakia or Slovak Republic or Djibouti or French Somaliland or Dominica or Dominican Republic or East Timor or East Timur or Timor Leste or Ecuador or Egypt or United Arab Republic or El Salvador or Eritrea or Estonia or Ethiopia or Fiji or Gabon or Gabonese Republic or Gambia or Gaza or Georgia Republic or Georgian Republic or Ghana or Gold Coast or Greece or Grenada or Guatemala or Guinea or Guam or Guiana or Guyana or Haiti or Honduras or Hungary or India or Maldives or Indonesia or Iran or Iraq or Isle of Man or Jamaica or Jordan or Kazakhstan or Kazakh or Kenya or Kiribati or Korea or Kosovo or Kyrgyzstan or Kirghizia or Kyrgyz Republic or Kirghiz or Kirgizstan or Lao PDR or Laos or Latvia or Lebanon or Lesotho or Basutoland or Liberia or Libya or Lithuania or Macedonia or Madagascar or Malagasy Republic or Malaysia or Malaya or Malay or Sabah or Sarawak or Malawi or Nyasaland or Mali or Malta or Marshall Islands or Mauritania or Mauritius or Agalega Islands or Mexico or Micronesia or Middle East or Moldova or Moldovia or Moldovian or Mongolia or Montenegro or Morocco or Ifni or Mozambique or Myanmar or Myanma or Burma or Namibia or Nepal or Netherlands Antilles or New Caledonia or Nicaragua or Niger or Nigeria or Northern Mariana Islands or Oman or Muscat or Pakistan or Palau or Palestine or Panama or Paraguay or Peru or Philippines or Philipines or Phillipines or Phillippines or Poland or Portugal or Puerto Rico or Romania or Rumania or Roumania or Russia or Russian or Rwanda or Ruanda or Saint Kitts or St Kitts or Nevis or Saint Lucia or St Lucia or Saint Vincent or St Vincent or Grenadines or Samoa or Samoan Islands or Navigator Island or Navigator Islands or Sao Tome or Saudi Arabia or Senegal or Serbia or Montenegro or Seychelles or Sierra Leone or Slovenia or Sri Lanka or Ceylon or Solomon Islands or Somalia or South Africa or Sudan or Suriname or Surinam or Swaziland or Syria or Tajikistan or Tadzhikistan or Tadjikistan or Tadzhik or Tanzania or Thailand or Togo or Togolese Republic or Tonga or Trinidad or Tobago or Tunisia or Turkey or Turkmenistan or Turkmen or Uganda or Ukraine or Uruguay or USSR or Soviet Union or Union of Soviet Socialist Republics or Uzbekistan or Uzbek or Vanuatu or New Hebrides or Venezuela or Vietnam or Viet Nam or West Bank or Yemen or Yugoslavia or Zambia or Zimbabwe or Rhodesia) Topic=(Africa or "Latin America" or "South America" or Caribbean or "West Indies" or "Eastern Europe" or Soviet or Arab or "Middle East" or "Latin America" or "Central America")
**JOLIS** (IMF, World Bank and International Finance Corporation)	http://external.worldbankimflib.org/uhtbin/cgisirsi/?ps=Uvm3MkrFSe/JL/0/49 (aggression OR violence OR homicide OR gang OR bully OR crime OR “juvenile delinquency” OR “conduct problem” OR “conduct disorder” OR “behavior problem” OR “behavior disorder”) AND (adolescent OR child OR youth OR student OR teen OR boy OR girl OR pupil OR youngster OR juvenile OR infant)
**World Bank**	https://openknowledge.worldbank.org/discover?scope=%2F&query=%28aggression+OR+violence+OR+homicide+OR+gang+OR+bully+OR+crime+OR+%E2%80%9Cjuvenile+delinquency%E2%80%9D+OR+%E2%80%9Cconduct+problem%E2%80%9D+OR+%E2%80%9Cconduct+disorder%E2%80%9D+OR+%E2%80%9Cbehavior+problem%E2%80%9D+OR+%E2%80%9Cbehavior+disorder%E2%80%9D%29+AND+%28adolescent+OR+child+OR+youth+OR+student+OR+teen+OR+boy+OR+girl+OR+pupil+OR+youngster+OR+juvenile+OR+infant%29&submit=Go (aggression OR violence OR homicide OR gang OR bully OR crime OR “juvenile delinquency” OR “conduct problem” OR “conduct disorder” OR “behavior problem” OR “behavior disorder”) AND (adolescent OR child OR youth OR student OR teen OR boy OR girl OR pupil OR youngster OR juvenile OR infant)
**LILACS**	**RUN 1**
	child OR niño OR criança OR infant OR lactante OR lactente OR Adolescent OR Adolescente OR “Child Psychiatry” OR “Psiquiatría Infantil” OR “Psiquiatria Infantil” OR “Child Behavior” OR “Conducta Infantil” OR “Comportamento Infantil” OR “Adolescent Behavior” OR “Conducta del Adolescente” OR “Comportamento do Adolescente” OR Adolescent Development” OR “Desarrollo del Adolescente” OR “Desenvolvimento do Adolescente” OR “Adolescent Behavior” OR “Conducta del Adolescente” OR “Comportamento do Adolescente”
	**[Subject descriptor]**
	**AND**
	gang OR gangs OR pandilla OR quadrilha OR crimes OR criminal OR Crimen OR Crime OR (antisocial AND behavio$r) OR antisocial OR anti‐social OR “antisocial behavio$r” OR “anti‐social behavior” OR “comportamento anti‐social” OR “conducta anti‐social” OR violen$ OR Violencia OR Violência OR violence OR violent OR violen$ OR bully$ OR “Acoso Escolar” OR Bullying OR aggress$ OR aggression OR Agresión OR Agressão OR Homicidio OR Homicídio OR Acoso Escolar OR bullying OR domestic violence OR Violencia Doméstica OR Violência Doméstica OR conducta antisocial
	**[Words]**
	**RUN** 2
	child OR children OR adolescent OR Adolescente OR child$ OR adolescen$ OR youth$ OR student$ OR teen$ OR boy$ OR girl$ OR pupil$ OR youngster$ OR juvenile$ OR infant$ OR infan$ OR baby OR babies OR preschool OR preschool$ OR criança OR infant OR infants OR lactante OR lactente OR neonat$ OR baby OR babies OR kid OR kids OR toddler$ OR jóvenes OR niña OR niño OR criança OR newborn
	**[Words]**
	**AND**
	“Domestic Violence” OR “Violencia Doméstica” OR “Violência Doméstica” OR “Social Behavior Disorders” OR “Trastorno de la Conducta Social” OR “Transtornos do Comportamento Social” OR aggression OR Agresión OR Agressão OR Homicide OR Homicidio OR Homicídio OR bully OR “Acoso Escolar” OR Bullying OR “oppositional defiant disorder” OR “trastorno desafiante por oposición” OR “transtorno desafiador de oposição” OR “conduct disorder” OR “Trastorno del Comportamiento” OR “Transtorno da Conduta” OR “transtorno desafiador‐opositivo” OR “conducta antisocial” or “transtorno da conduta” OR “transtorno da personalidade anti‐social” OR “Transtornos do Comportamento”
	**[Subject descriptor]**
	**RUN** 3
	child OR niño OR criança OR infant OR lactante OR lactente OR Adolescent OR Adolescente OR “Child Psychiatry” OR “Psiquiatría Infantil” OR “Psiquiatria Infantil” OR “Child Behavior” OR “Conducta Infantil” OR “Comportamento Infantil” OR “Adolescent Behavior” OR “Conducta del Adolescente” OR “Comportamento do Adolescente” OR Adolescent Development” OR “Desarrollo del Adolescente” OR “Desenvolvimento do Adolescente” OR “Adolescent Behavior” OR “Conducta del Adolescente” OR “Comportamento do Adolescente” OR “Adolescent Psychiatry” OR “Psiquiatría del Adolescente” OR “Psiquiatria do Adolescente”
	**[Subject descriptor]**
	**AND**
	“Domestic Violence” OR “Violencia Doméstica” OR “Violência Doméstica” OR “Social Behavior Disorders” OR “Trastorno de la Conducta Social” OR “Transtornos do Comportamento Social” OR aggression OR Agresión OR Agressão OR Homicide OR Homicidio OR Homicídio OR bully OR “Acoso Escolar” OR Bullying OR “oppositional defiant disorder” OR “trastorno desafiante por oposición” OR “transtorno desafiador de oposição” OR “conduct disorder” OR “Trastorno del Comportamiento” OR “Transtorno da Conduta” OR “transtorno desafiador‐opositivo” OR “conducta antisocial” or “transtorno da conduta” OR “transtorno da personalidade anti‐social” OR “Transtornos do Comportamento”
	**[Subject descriptor]**
	**RUN 4**
	child OR children OR adolescent OR Adolescente OR child$ OR adolescen$ OR youth$ OR student$ OR teen$ OR boy$ OR girl$ OR pupil$ OR youngster$ OR juvenile$ OR infant$ OR infan$ OR baby OR babies OR preschool OR preschool$ OR criança OR infant OR infants OR lactante OR lactente OR neonat$ OR baby OR babies OR kid OR kids OR toddler$ OR jóvenes OR niña OR niño OR criança OR newborn
	**[Words]**
	**AND**
	gang OR gangs OR pandilla OR quadrilha OR crimes OR criminal OR Crimen OR Crime OR antisocial OR anti‐social OR “antisocial behavio$r” OR “anti‐social behavior” OR “comportamento anti‐social” OR “conducta anti‐social” OR “conducta antisocial” OR violen$ OR Violencia OR Violência OR violence OR violent OR bully$ OR “Acoso Escolar” OR Bullying OR aggress$ OR aggression OR Agresión OR Agressão OR Homicidio OR Homicídio OR Acoso Escolar OR “domestic violence” OR “Violencia Doméstica” OR “Violência Doméstica”
	**[Words]**
	**NOT**
	liposarcoma
	**RUN 5**
	“Child Behavior Disorders” OR “delinquencia” OR “delinquencia femenina” OR “delinquencia juvenil” or delincuencial or delincuenciales or delincuente or delincuentes OR “juvenile delinquency” OR delincuen$ OR “Delincuencia Juvenil” OR “Delinquência Juvenil” OR “Transtornos do Comportamento Infantil” OR Delinquencia or Delinquen$ or “Trastornos de la Conducta Infantil” or Transtornos do “Comportamento Infantil”
	**[Words]**
SciELO	www.scielo.br
	http://www.scielo.br/cgi‐bin/wxis.exe/iah/
	**RUN 1**
	child OR niño OR criança OR infant OR lactante OR lactente OR Adolescent OR Adolescente OR child OR children OR adolescent OR child$ OR adolescen$ OR youth$ OR student$ OR teen$ OR boy$ OR girl$ OR pupil$ OR youngster$ OR juvenile$ OR infant$ OR infan$ OR baby OR babies OR preschool OR preschool$ OR criança OR infant OR infants OR lactante OR lactente OR neonat$ OR baby OR babies OR kid OR kids OR toddler$ OR jóvenes OR niña OR niño OR criança OR newborn
	[All indexes]
	AND
	“Acoso Escolar” OR “Violência Doméstica” OR Transtornos do Comportamento OR “Transtornos do Comportamento Social” OR Agressão OR Homicídio OR Bullying OR “transtorno desafiador‐opositivo” OR “Transtorno da Conduta” OR “conducta antisocial” or “transtorno da conduta” OR “transtorno da personalidade anti‐social”
	[Subject descriptor]
	**RUN 2**
	child OR niño OR criança OR infant OR lactante OR lactente OR Adolescent OR Adolescente OR child OR children OR adolescent OR child$ OR adolescen$ OR youth$ OR student$ OR teen$ OR boy$ OR girl$ OR pupil$ OR youngster$ OR juvenile$ OR infant$ OR infan$ OR baby OR babies OR preschool OR preschool$ OR criança OR infant OR infants OR lactante OR lactente OR neonat$ OR baby OR babies OR kid OR kids OR toddler$ OR jóvenes OR niña OR niño OR criança OR newborn
	[All indexes]
	AND
	gang OR gangs OR pandilla OR quadrilha OR crimes OR criminal OR crimen OR crime OR “comportamento anti‐social” OR “conducta anti‐social” OR violence OR violen$ OR Violencia OR Violência OR violent OR bully$ OR aggress$ OR aggression OR Agresión OR Agressão OR Homicidio OR Homicídio OR Acoso Escolar OR bullying OR domestic violence OR Violencia Doméstica OR Violência Doméstica OR conducta antisocial OR “Transtorno da Conduta” OR “transtorno desafiador de oposição” OR “transtorno da personalidade anti‐social”OR “Transtornos do Comportamento”
	[All indexes]
	**RUN 3**
	“delinquencia” OR “delinquencia femenina” OR “delinquencia juvenil” or delincuencial or delincuenciales or delincuente or delincuentes OR “Transtornos do Comportamento Infantil” [Subject descriptor]
	**RUN 4**
	Delinquencia or Delinquen$ or Transtornos do Comportamento Infantil [All indexes]

## Appendix B: Document coding protocol

**Reference information**

1. Document ID
2. Study author/s
3. Study title
4. Publication year
5. Full APA‐style reference
6. Reference type:
  a. Book
  b. Journal article (peer reviewed)
  c. Dissertation or thesis
  d. Government report
  e. Police report
  f. Technical report
  g. Conference paper
  h. Other (specify)_____________________
7. Coder's name; date coded

**Study details (complete for each study reported)**

8. Country of intervention _________________________
9. Document language ___________________________
10. Date of research
  a. Start: ____________
  b. Finish: ____________
11. Source of funding for study
  a. Government
  b. Foreign government
  c. Local university/research body
  d. Foreign university/research body
  e. Other _________________
12. Bodies involved (tick all applicable)
  a. Police/ Justice system
  b. Health Service
  c. Other government departments
  d. University/research agency
  e. Other ____________________
13. Evaluated by ____________________________
14. Conflict context?
  a. Yes
  b. No
15. Ethical issues?
  a. Yes (describe) ________________________________
  b. No

**Methodology**

16. Type of study:
  a. Longitudinal
  b. Cross‐sectional
  c. Case control
17. Comparison group present?
  a. Matched
    i. Statistical post‐hoc
    ii. Propensity matching
    iii. Case control
  b. Unmatched
18. Unit of analysis _______________
19. Measure of gang involvement:
  a. Gang membership
  b. Gang affiliation
  c. Involvement in gang‐related crime
  d. Ex‐gang member
  e. Other_________________________
20. Source of gang involvement measure:
  a. Obtained from official data (government/police)
  b. Self‐reported
  c. Peer‐reported
  d. Family‐reported
  e. Practitioner‐reported
  f. Other _________________________
21. Term/s used by author to describe gang:
  a. Gang
  b. Pandilla
  c. Maras
  d. Street children
  e. Other___________________
22. Author definition of gang:
  a. Eurogang definition
  b. Not specified
  c. Other ___________
23. Sample size
  a. Total sample size ____________________________
  b. Sample size of comparison group______________________
  c. Sample size of gang‐involved group ______________________
24. Was attrition a problem?
  a. Yes (describe) ____________________
  b. No
  c. Not applicable
25. Initial response rate_____________________
26. Sample age _____________________
27. Sample gender
  a. Male
  b. Female
  c. Mixed
28. Sample socio‐economic status
  a. Low
  b. Average
  c. High
  d. Mixed
  e. Other_____________________

**Risk of bias**

29. Study population description. Does the document describe the source population in replicable detail?
  a. Yes
  b. No
  c. Unclear
30. Study population criteria: Does the document list all inclusion and exclusion criteria for participation?
  a. Yes
  b. No
  c. Unclear
31. Prospective study: Was the study prospective (ie the sample was selected prior to the onset of gang membership)?
  1. Yes
  2. No
  3. Unclear
32. Outcome descriptor: Were the gang membership criteria described in replicable detail?
  a. Yes
  b. No
  c. Unclear
33. Predictor description: Were all predictors described in replicable detail?
  a. Yes
  b. No
  c. Unclear
34. Predictor validity: Were all measures of the predictors based on a validated measure?
  a. Yes
  b. No
  c. Unclear
35. Predictor timing: Were all predictors either measured before the onset of gang membership or measured retrospectively to a time prior to gang membership?
  a. Yes
  b. No
  c. Unclear
36. Selective predictor reporting: was the study free from predictor reporting bias?
  a. Yes
  b. No
  c. Unclear
37. Selective analysis reporting: was the study free from analysis reporting bias?
  a. Yes
  b. No
  c. Unclear

**Predictors (complete for each predictor reported)**

38. Predictor ________________________________
39. Conceptual definition of predictor ________________________
40. Operation definition ______________________________
41. Where was the predictor variable obtained?
  a. Official data (government/police)
  b. Self‐reported
  c. Peer‐reported
  d. Family‐reported
  e. Practitioner‐reported (including school)
  f. Other _________________________
42. Measured retrospectively?
  1) Yes
  2) No
  3) Unclear
43. Time‐invariant predictor? (if the study design is not longitudinal and the factor is not time‐invariant, the predictor will be classified as a correlate)
  a. Yes
  b. No
  c. Unclear
44. Age group associated with predictor
  a. Under 6 years
  b. 6‐11 years
  c. 12‐14 years
  d. Over 14 years
  e. Other age categorisation___________________
45. Predictor domain
  a. Individual
  b. Peer
  c. Family
  d. School
  e. Community
  f. Other _____________________
46. Raw difference shows predictor is more likely to occur in:
  a. Gang‐involved group
  b. Comparison group
  c. Neither (exactly equal)
  d. Cannot tell (or statistically insignificant report only)
47. Did a test of statistical significance indicate statistically significant differences between the comparison and gang‐involved groups?
  a. Yes
  b. No
  c. Can't tell
  d. N/A (no testing completed)
48. Was a standardized effect size reported?
  a. Yes
  b. No

*If Yes:*

49. Effect size measure______________
50. Effect size___________________
51. Standard error of effect size________________
52. Effect size reported on page number_________________

*If No:*

53. Are data available to calculate effect size?
  d. Yes
  e. No
54. Type of data effect size can be calculated from:
  a. Means and standard deviations
  b. Frequencies or proportions (dichotomous)
  c. Frequencies or proportions (polychotomous)
  d. Unadjusted correlation coefficient
  e. Multiple regression coefficients (unstandardized)
  f. Multiple regression coefficients (standardized)
  g. *t*‐value or *F*‐value
  h. Chi‐square (df=1)
  i. Other (specify) _________

*Means and Standard Deviations*

55. Gang‐involved group mean. _____
56. Comparison group mean. _____
57. Gang‐involved group standard deviation. _____
58. Comparison group standard deviation. _____

*Proportions or frequencies*

59. *n* of gang‐involved group with the predictor. _____
60. *n* of comparison group with the predictor. _____
61. Proportion of gang‐involved group with the predictor. _____
62. Proportion of comparison group with the predictor. _____

*Regression coefficients and correlations*

63. Unadjusted correlation coefficient___________
64. Standardized regression coefficient______
65. Unstandardized regression coefficient______
66. Standard deviation of predictor _______
67. Control variables _________________________________

*Significance Tests*

68. *t*‐value _____
69. *F*‐value _____
70. Chi‐square value (*df*=1) _____

*Calculated Effect Size*

71. Effect size ______
72. Standard error of effect size _____

**Authors’ conclusion**

73. What did the authors conclude about the relationship?
  a. Predictor increases gang membership
  b. Predictor reduces gang membership
  c. No effect of predictor on gang membership
  d. Unclear/no conclusion stated by authors

## References

[cl2014001032-bib-0001] Abramovay, M., Jacob Waiselfisz, J., Coelho Andrade, C. and das Gracas Rua, M. (1999) Gangs, crews, buddies and rappers: Youth violence and citizenship around the outskirts of Brasilia. Brazil: UNESCO Brazil.

[cl2014001032-bib-0002] Celbis, O., Karaoglu, L., Egri, M., and Özdemir, B. (2012). Violence among high school students in Malatya: A prevalence study. Turkish Journal of Medical Science, 42(2), 343–50.

[cl2014001032-bib-0003] Katz, C. M., and Fox, A. M. (2010). Risk and protective factors associated with gang‐involved youth in Trinidad and Tobago. Revista Panamericana de Salud Pública, 27(3), 187–202. 20414508 10.1590/s1020-49892010000300006

[cl2014001032-bib-0004] Ohene, S. A. (2005). The clustering of risk behaviors among Caribbean youth. Maternal and Child Health Journal, 9(1), 91–100. 15880978 10.1007/s10995-005-2452-6

[cl2014001032-bib-0005] Olate, R., Salas‐Wright, C., and Vaughn, M. G. (2012). Predictors of violence and delinquency among high risk youth and youth gang members in San Salvador, El Salvador. International Social Work, 55(3), 383–401.

[cl2014001032-bib-0006] Olate, R., Salas‐Wright, C., and Vaughn, M.G. (2011). A cross‐national comparison of externalizing behaviors among high‐risk youth and youth gang members in Metropolitan Boston, Massachusetts, and San Salvador, El Salvador. Victims and Offenders: An International Journal of Evidence‐based Research, Policy, and Practice, 6 (4), pp. 356–369. DOI: 10.1080/15564886.2011.607396.

[cl2014001032-bib-0007] Pyrooz, D. C., and Decker, S. H. (2013). Delinquent behavior, violence, and gang involvement in China. Journal of Quantitative Criminology, 29:251–272.

[cl2014001032-bib-0008] Webb VJ, Ren L, Zhao J, He N, Marshall IH (2011) A comparative study of youth gangs in China and the United States: definition, offending and victimization. Int Crim Justice Rev 21:225–242

[cl2014001032-bib-0009] Moravcova, E. (2012). Methodological aspects of gang membership: The case of the Czech Republic. Acta Universitatis Carolinae Philosophica et Historica, s. 69–83. ISSN 0567‐8293.

[cl2014001032-bib-00010] Rubio, M. (2007). de La Pandilla a la Mara: Pobreza, Educacion, Mujeres y Violencia Juvenil. Universidad Externado de Colombia. ISBN‐10: 9587102177

[cl2014001032-bib-00011] Rubio, M. (2003). Maras y delincuencia juvenil en Centroamerica. Retrieved from http://ww.centroamericajoven.org/sites/default/files/Maras%20y%20delincuencia%20juvenil%20en%20Centroamrica.pdf

[cl2014001032-bib-00012] Adams, T. M. (2012). Chronic Violence and its Reproduction: Perverse Trends in Social Relations, Citizenship, and Democracy in Latin America. Citizen Security and Organized Crime. Washington: Woodrow Wilson Centre.

[cl2014001032-bib-00013] Baird, A. (2012). The Violent Gang and the Construction of Masculinity Amongst Socially Excluded Young Men. Safer Communities: A Journal of Practice, Opinion, Policy and Research, 11(4).

[cl2014001032-bib-00014] Battin, S. R., Hill, K. G., Abbott, R. D., Catalano, R. F., and Hawkins, J. D. (1998). The contribution of gang membership to delinquency beyond delinquent friends. Criminology, 36(1), 93–116.

[cl2014001032-bib-00015] Blum, R. W., Halcon, L., Beuhring, T., Pate, E., Campell‐Forrester, S. and Venema, A. (2003). Adolescent health in the Caribbean: risk and protective factors. American Journal of Public Health, 93(3), 456–460. 12604495 10.2105/ajph.93.3.456 PMC1447763

[cl2014001032-bib-00016] Borenstein, M. (2009). Effect sizes for continuous data. In The handbook of research synthesis. 2nd edition. Cooper, H., Hedges, L.V., and Valentine, J.C. (Eds) (pp. 221–235). Russell Sage Foundation: New York, New York.

[cl2014001032-bib-00017] Cruz, J. M. (2007). Factors associated with juvenile gangs in Central America. In J. M. Cruz (Ed.) Street Gangs in Central America (pp. 13–65). San Salvador: UCA Editores.

[cl2014001032-bib-00018] Dahlberg, L. L. (1998). Youth violence in the United States: Major trends, risk factors, and prevention approaches. American Journal of Preventive Medicine, 14(4), 259–272. doi: 10.1016/s0749‐3797(98)00009‐9 9635070 10.1016/s0749-3797(98)00009-9

[cl2014001032-bib-00019] Davies, P., and MacPherson, K. (2011). Why is crime in South Africa so violent?: A rapid evidence assessment. Retrieved from http://www.pan.org.za/node/8682

[cl2014001032-bib-00020] Decker, S.H., Melde, C., and Pyrooz, D.C. (2013) What Do We Know About Gangs and Gang Members and Where Do We Go From Here?, Justice Quarterly, 30:3, 369–402, DOI: 10.1080/07418825.2012.732101

[cl2014001032-bib-00021] Decker, S.H., and Pyrooz, D.C. (2010) Gang violence across the world. In Small arms survey 2010

[cl2014001032-bib-00022] de Vibe, M., Bjorndal, A., Tipton, E., Hammerstrom, K., and Kowalski, K. (2012). Mindfulness based stress reduction (MBSR) for improving health, quality of life, and social functioning in adults. The Campbell Collaboration Library of Systematic Reviews, 8 (3). http://campbellcollaboration.org/lib/project/117/

[cl2014001032-bib-00023] de Vibe, M., Bjorndal, A., Tipton, E., Hammerstrom, K., and Kowalski, K. (2012). Mindfulness based stress reduction (MBSR) for improving health, quality of life, and social functioning in adults. The Campbell Collaboration Library of Systematic Reviews, 8 (3). http://campbellcollaboration.org/lib/project/117/

[cl2014001032-bib-00024] EPPI‐Centre (2009). Reducing gang related crime: A systematic review of ‘comprehensive interventions.’ Retrieved from http://eppi.ioe.ac.uk/cms/Default.aspx?tabid=2444&language=en‐US

[cl2014001032-bib-00025] Esbensen, F‐A., Winfree, L. T., He, N., and Taylor, T. J. (2001). Youth gangs and definitional issues: When is a gang a gang, and why does it matter? Crime and Delinquency, 47(1), 105–130. doi: 10.1177/0011128701047001005

[cl2014001032-bib-00026] Farrington, D.P., and Loeber, R. (2000). Epidemiology of juvenile violence. Child and Adolescent Psychiatric Clinics of North America, 94(4), 733–748. 11005003

[cl2014001032-bib-00027] Fisher, H. Montgomery, P. and Gardner, F. (2008a). Cognitive‐behavioural interventions for preventing youth gang involvement for children and young people. Campbell Systematic Reviews, 7. doi: 10.4073/csr.2008.7 10.1002/14651858.CD007008.pub2 PMC12526719 18425976

[cl2014001032-bib-00028] Fisher, H. Montgomery, P. and Gardner, F (2008b). Opportunities provision for preventing youth gang involvement for Children and Young People (7‐16). Campbell Systematic Reviews, 8. doi: 10.4073/csr.2008.8 10.1002/14651858.CD007002.pub2 PMC6464132 18425975

[cl2014001032-bib-00029] Gatti, U., Haymoz, S., and Schadee, H. M. A. (2011). Deviant Youth Groups in 30 Countries: Results From the Second International Self‐Report Delinquency Study. International Criminal Justice Review, 21(3), 208–224. doi: 10.1177/1057567711418500

[cl2014001032-bib-00030] Haviland, A., Rosenbaum, P. R., Nagin, D. S., and Tremblay, R. E. (2008). Combining group‐based trajectory modeling and propensity score matching for causal inferences in nonexperimental longitudinal data. Developmental Psychology, 44(2), 422–436. 18331133 10.1037/0012-1649.44.2.422

[cl2014001032-bib-00031] Hawkins, J.D., Herrenkohl, T. I., Farrington, D.P., Brewer, D., Catalano, R.F., Harachi, T.W., and Cothern, L. (2000). Predictors of youth violence. Office of Juvenile Justice and Delinquency Prevention Juvenile Justice Bulletin, April 2000.

[cl2014001032-bib-00032] Herrenkohl, T. I., Maguin, E., Hill, K. G., Hawkins, J. D., Abbott, R. D., and Catalano, R. F. (2000). Developmental risk factors for youth violence. The Journal of Adolescent Health, 26(3), 176–186. 10706165 10.1016/s1054-139x(99)00065-8

[cl2014001032-bib-00033] Higginson, A., Benier, K., Shenderovich, Y., Bedford, L., Mazerolle, L., and Murray, J. (2015). Preventive interventions for reducing youth gang violence in low‐ and middle‐income countries: A systematic review, Campbell Systematic Reviews, Retrieved from http://campbellcollaboration.org/lib/project/297/ 10.4073/csr.2018.11 PMC8427992 37131383

[cl2014001032-bib-00034] Higginson, A., Mazerolle, L., Benier, K., and Bedford, L. (2013). Title registration for a systematic review: Predictors of youth gang membership in low‐ and middle‐income countries: A systematic review, Campbell Systematic Reviews, Retrieved from: https://www.campbellcollaboration.org/library/predictors‐of‐youth‐gang‐membership‐low‐and‐middle‐income‐countries.html 10.4073/csr.2018.11 PMC8427992 37131383

[cl2014001032-bib-00035] Higginson, A., Benier, K., Shenderovich, Y., Bedford, L., Mazerolle, L., and Murray, J. (2014). Protocol: Predictors of youth gang membership in low‐ and middle‐income countries: A systematic review, Campbell Systematic Reviews, Retrieved from: https://www.campbellcollaboration.org/library/predictors‐of‐youth‐gang‐membership‐low‐and‐middle‐income‐countries.html 10.4073/csr.2018.11 PMC8427992 37131383

[cl2014001032-bib-00036] Howell, J. C. (1997). Youth gangs. U.S. Department of Justice ‐ OJJDP fact sheet. Retrieved from https://www.ncjrs.gov/pdffiles/fs‐9772.pdf

[cl2014001032-bib-00037] Howell, J. C. (1998). Youth gangs: An overview. Washington, DC: Office of Juvenile Programs, US Department of Justice.

[cl2014001032-bib-00038] Howell, J. C. (2012). Gangs in America's communities. Washington, DC: Sage.

[cl2014001032-bib-00039] Howell, J.C. and Egley, A. (2005). Moving Risk Factors into Developmental Theories of Gang Membership. Youth Violence and Juvenile Justice, 3(4), 334–354.

[cl2014001032-bib-00040] Howell, J. C., Egley, A., and O'Donnell, C. (n.d.). National Gang Center ‐ Frequently asked questions about gangs. Retrieved from http://www.nationalgangcenter.gov/About/FAQ

[cl2014001032-bib-00041] Huff, C. R. (1993). Gangs in the United States. In A. P. Goldstein and C. R. Huff (Eds.), The gang intervention handbook. Champaign, IL: Research Press.

[cl2014001032-bib-00042] Katz, C. M., and Fox, A. M. (2010). Risk and protective factors associated with gang involved youth in Trinidad and Tobago. Pan‐American Journal of Public Health, 27(3), 187–202 20414508 10.1590/s1020-49892010000300006

[cl2014001032-bib-00043] Klein, M. (2002). Street gangs: A cross‐national perspective. In R. Huff (Ed.), Gangs in America (3rd ed.). Thousand Oaks, CA: Sage.

[cl2014001032-bib-00044] Klein, M. and Maxson, C. (2006). Street gang patterns and policies. New York: Oxford University Press.

[cl2014001032-bib-00045] Lipsey, M.W., and Derzon, J.H. (1999). Predictors of violent or serious delinquency in adolescence and early adulthood. In Loeber, R., and Farrington, D.P. (Eds) Serious and Violent Juvenile Offenders: Risk Factors and Successful Interventions, Thousand Oaks, CA: Sage.

[cl2014001032-bib-00046] Moser, C., and Holland, J. (1997). Urban poverty and violence in Jamaica. Washington, DC: World Bank. doi: 10.1596/978‐0‐8213‐3870‐4

[cl2014001032-bib-00047] Muggah, R., and Aguirre, K. (2013). Assessing and responding to youth violence in Latin America: Surveying the evidence. Retrieved from www.worldwewant2015.org/file/302730/download/328436

[cl2014001032-bib-00048] Murray, J., Farrington, D. P., and Eisner, M. P. (2009). Drawing conclusions about causes from systematic reviews of risk factors: The Cambridge Quality Checklists. Journal of Experimental Criminology, 5(1), 1–23. doi:10.1007/s11292‐008‐9066‐0

[cl2014001032-bib-00049] Murray, J., Shenderovich, Y., Eisner, M., Ttofi, M., Mikton, C., Gardner, F., Parker, R. (2013) Risk Factors for Child and Adolescent Conduct Problems and Youth Crime and Violence in Low‐ and Middle‐Income Countries: Two‐Part Systematic Review. Retrieved from http://www.crim.cam.ac.uk/research/vrc/projects/lmic_risk_factor_review_protocol_2013.pdf

[cl2014001032-bib-00050] O'Brien, K., Daffern, M., Chu, C M., and Thomas, S.D.M. (2013). Youth gang affiliation, violence, and criminal activities: A review of motivational, risk, and protective factors. Aggression and Violent Behavior, 18, 4, 417–425. doi: 10.1016/j.avb.2013.05.001

[cl2014001032-bib-00051] Olate, R., Salas‐Wright, C. and Vaughn M. (2011) A cross national comparison of externalizing behaviours among high risk youth and youth gang members in metropolitan Boston, Massachusetts, and San Salvador, El Salvador, Victims and Offenders, 6, 356–369.

[cl2014001032-bib-00052] Olate, R., Salas‐Wright, C., and Vaughn, M. G. (2012). Predictors of violence and delinquency among high risk youth and youth gang members in San Salvador, El Salvador. International Social Work, 55(3), 383–401. doi: 10.1177/0020872812437227

[cl2014001032-bib-00053] Organization of American States (OAS) . (2007). Definition and classification of gangs: Executive summary. Washington, DC: Department of Public Security.

[cl2014001032-bib-00054] Raine A., Brennan, P., Mednick, B., and Mednick, S. A. (1996). High rates of violence, crime, academic problems and behavioral problems in males with boht early neuromotor deficits and unstable family environments. Archives of General Psychiatry, 53(6), 544–549. doi:10.1001/archpsyc.1996.01830060090012. 8639038 10.1001/archpsyc.1996.01830060090012

[cl2014001032-bib-00055] Rodgers, D. (1999). Youth gangs and violence in Latin America and the Caribbean: A literature survey. Sustainable Development Working Paper No. 4. Washington, DC: World Bank.

[cl2014001032-bib-00056] Rothstein, H. R., Sutton, A. J., and Borenstein, M. (2005). Publication bias in meta‐analysis: prevention, assessment and adjustments. Hoboken, NJ: Wiley.

[cl2014001032-bib-00057] Sanders, B., Schneiderman, J. U., Loken, A., Lankenau, S. E., and Bloom, J. J. (2009). Gang youth as a vulnerable population for nursing intervention. Public Health Nursing, 26(4), 346–352. doi: 10.1111/j.1525‐1446.2009.00789.x 19573213 10.1111/j.1525-1446.2009.00789.x

[cl2014001032-bib-00058] Seelke, C. R. (2013). Gangs in Central America. Washington, DC: Congressional Research Service.

[cl2014001032-bib-00059] Shenderovich, Y., Eisner, M., Mikton, C., Gardner, F., Liu, J., and Murray, J. (2016). Methods for conducting systematic reviews of risk factors in low‐ and middle‐income countries. BMC Medical Research Methodology, 16:32, DOI 10.1186/s12874‐016‐0134‐2 26979282 10.1186/s12874-016-0134-2 PMC4791911

[cl2014001032-bib-00060] Small Arms Survey . (2010). Gangs groups and guns. Geneva: The Graduate Institute. Retrieved from http://www.smallarmssurvey.org/?id=286

[cl2014001032-bib-00061] Thale, G., and Falkenburger, E. (2006). Youth gangs in Central America: Issues in human rights, effective policing and prevention. Washington DC: Washington Office on Latin America.

[cl2014001032-bib-00062] Thornberry, T.P. (1999). Membership in youth gangs and involvement in serious and violent offending. In Loeber, R., and Farrington, D.P. (Eds) Serious and violent juvenile offenders: risk factors and successful interventions. Thousand Oaks, CA: Sage.

[cl2014001032-bib-00063] Thornberry, T. P., Krohn, M. D., Lizotte, A. J., and Chard‐Wierschem, D. (1993). The role of juvenile gangs in facilitating delinquent behavior. Journal of Research in Crime and Delinquency, 30(1), 55–87. doi: 10.1177/0022427893030001005

[cl2014001032-bib-00064] Tobin, K. (2008). Gangs: An individual and group perspective Upper Saddle River, NJ: Prentice Hall.

[cl2014001032-bib-00065] UNODC . (2007). Crime and Development in Central America: Caught in the Crossfire Vienna: United Nations.

[cl2014001032-bib-00066] Weerman, F. M., Maxson, C. L., Esbensen, F‐A., Aldridge, J., Medina, J., and van Gemert, F. (2009). Eurogang Program Manual: Background, development, and use of the Eurogang instruments in multi‐site, multi‐method comparative research. Retrieved from http://www.umsl.edu/ccj/eurogang/EurogangManual.pdf

[cl2014001032-bib-00067] White, R. (2002). Understanding youth gangs. Trends and Issues in Crime and Criminal Justice, No. 237 Canberra, Australia: Australian Institute of Criminology.

[cl2014001032-bib-00068] World Bank . (2013). Country and lending groups. Retrieved from http://data.worldbank.org/about/country‐classifications/country‐and‐lending‐groups

